# Diversity and Impacts of Mining on the Non-Volant Small Mammal Communities of Two Vegetation Types in the Brazilian Amazon

**DOI:** 10.1371/journal.pone.0167266

**Published:** 2016-11-28

**Authors:** Natália Carneiro Ardente, Átilla Colombo Ferreguetti, Donald Gettinger, Pricila Leal, Ana Cristina Mendes-Oliveira, Fernanda Martins-Hatano, Helena Godoy Bergallo

**Affiliations:** 1 Universidade do Estado do Rio de Janeiro, Instituto de Biologia Roberto Alcântara Gomes, Laboratório de Ecologia de Mamíferos, Rio de Janeiro, Rio de Janeiro, Brazil; 2 Harold W. Manter Laboratory of Parasitology, University of Nebraska-Lincoln, Lincoln, Nebraska, United States of America; 3 Universidade Federal Rural da Amazônia Biologia, Departamento de Biologia Animal, Belém, Pará, Brazil; 4 Universidade Federal do Pará, Instituto de Ciências Biológicas, Laboratório de Ecologia e Zoologia de Vertebrados, Campus Básico—Rua Augusto Corrêa, Belém—Pará —Brasil; University of Missouri Kansas City, UNITED STATES

## Abstract

The Carajás National Forest contains some of the largest iron ore deposits in the world. The majority of the minerals are found below a plant community known as Savana Metalófila, or “Canga”, which represents only 3% of the landscape within the Carajás National Forest (CNF). The aim of our study was to understand the diversity of community of non-volant small mammals in the two predominant vegetation types: Ombrophilous Forest and Canga, and to examine how mining impacts these communities. Sampling was conducted from January 2010 to August 2011 in 11 sampling sites divided by the total area of Canga and 12 sampling sites in the forest, totalizing 23 sites. Of these, 12 sites (Canga and Forest) were considered impacted areas located close to the mine (<< 900 meters) and 11 sites (Canga and Forest), serving as controls, which were at least 7,000 meters from the mine. We recorded 28 species, 11 from the Order Didelphimorphia and 17 from the Order Rodentia. The two forest types shared 68.42% of the species found in the CNF. A gradient analysis (Non-metric multidimensional scaling) revealed that the first axis clearly separated the non-flying small mammal communities by vegetation type. Occupancy models showed that the detectability of species was affected by the distance from the mining activities. Of all the small mammals analyzed, 10 species were positively affected by the distance from mining in areas impacted (e.g. more likely to be detected farther from mining areas) and detectability was lower in impacted areas. However, three species were negatively affected by the distance from mining, with higher detectability in the impacted areas, and seven species showed no effect of their proximity to mining operations. To date, there are no studies in Brazil about the impact of mining on mammals or other vertebrates. This study reveals that the effect of mining may go beyond the forest destruction caused by the opening of the mining pits, but also may negatively affect sensitive wildlife species.

## Introduction

The Amazonian Biome, with its wide variety of habitats, has the highest diversity of non-volant small mammals (Orders Didelphimorphia and Rodentia) in the world, with 83 endemic species of marsupials and rodents. Over the last 20 years, 92 species of mammals have been described worldwide, and 74% are exclusive to Brazil, mostly from the Brazilian Amazon [1]. Currently, with the development of cytogenetic and molecular techniques, and renewed efforts to survey mammals in previously unexplored areas, one can predict that still more new species will be discovered throughout the Amazon [1].

With the discovery and development of natural resources in the last 50 years, deforestation has intensified in Amazonia. The establishment of Carajás Project has brought mining activities to the region [2] and the Serra dos Carajás, in eastern Amazonia, Brazil, holds the largest iron ore deposits in the world [2]. The iron ore extracted from the Serra dos Carajás (i.e. the Carajás National Forest, “CNF”) is found under a vegetation type known as Savana Metalófila or “Canga”, which covers about 3% of the landscape, and the rest of the CNF is dominated by Ombrophilous forests [3].

There are several inevitable impacts of mining activity, such as generated waste, vegetation suppression to expand the extraction area and/or by the opening of access roads, accidents in the transportation of minerals and toxic byproducts, and the disposal of tailings and wastes [4,5]. In the CNF, most of the impacts are related to removal of vegetation, since the extractions are achieved with surface operations, or “open pit mines”. The mining process radically modifies the microhabitat complexity [6], leading not only to habitat loss but the addition of new structures, such as drainage ditch networks [7]. These drainage ditches make topographic gaps across the surfaces of deforested land, which are widely used by non-volant small mammals [6,8,9].

The impact of mining on small mammals is poorly understood. Currently only a few studies have been conducted (mainly in the United States), including research on the small mammal communities in areas previously mined in Pennsylvania [8], Ohio [10], Colorado [9], the northern plains of the US [11], areas recovered from mining in southern West Virginia [12], southeastern Virginia [13], and eastern Kentucky [6].

Our study attempts to verify the community structure of non-volant small mammals in the two primary vegetation types of the CNF (e.g. Ombrophilous Forest and Canga), and to assess the impact of mining on each species through occupancy and detectability models using the distance from the mine as representative of the impact. Based on this objective we developed the following hypotheses: 1) the diversity of the small mammal community is different between the two sampled vegetation types, due to differences in the complexity of each vegetation type, and species richness will be higher in Ombrophilous Forest areas; and 2) capture rates (i.e. species detectability) are lower in areas closest to the mine, especially in the Ombrophilous Forest because this vegetation type presents more complex microhabitats and therefore will be more affected.

## Materials and Methods

### Study area

The Carajás National Forest (CNF) encompasses 411,948.87 hectares and is located in southeastern Pará, Brazil (05°52’ - 06°33`S and 49°53–50°45`W), where there is the mining activity of Vale company.

**Fig 1:**
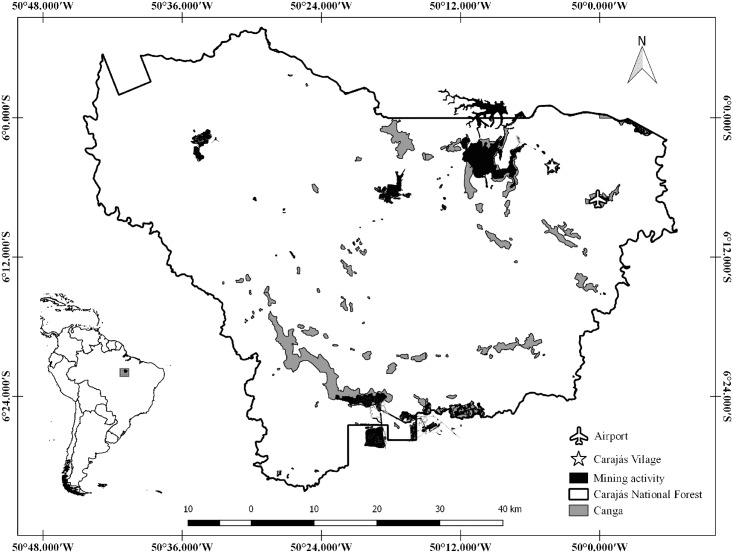
The map of the location of our study area, Carajás National Forest, Para, Brazil, highlighting the presence of Canga areas and the activity mining areas of Vale company.

The CNF is a reserve created in 1998 for sustainable use to ensure the preservation of renewable resources and ecological processes [14]. The elevation of CNF ranging from 600 to 800 m, 96.3% of the area is composed of Ombrophilous Forest and 2.3% by natural clearings with Savana Metalófila or Canga, which is a vegetation that grows on complex geological formations, known as "canga hematítica" [15,16]. The canga hematítica is a rocky layer that covers the deposit of iron ore (Fig 1).

In general, the tree canopy in the CNF is about 30 m high, with emergent trees that reach 50 m. The understory consists of regenerating tree seedlings, palms, shrubs, and lianas. The Canga is composed of well-defined open areas, surrounded by forest [16]. The plant species present in this formation are typical of forests or savannahs and are rare in the area. Natural grasslands occur where the terrain is semi-flat or concave with rocky outcrop, which are highly impermeable, and water accumulates in the rainy season, allowing plant species with short life cycles to develop [16]. The xerophytic vegetation includes species that are adapted to extremely adverse environments, occurring in every Canga area, mainly in the rugged areas. In this formation, the surface of the ground is covered by a continuous grassy mat [16].

### Sampling design

We conducted this study with field permit: IBAMA license 30-B/2008 MAB / FAUNA, 02018.001735/2006-91 process. Sampling was conducted from January 2010 to August 2011 in 11 sampling sites divided by the total area of Canga and 12 sampling sites in the forest, totaling 23 sites. Of these, 12 sites (Canga and forest) were considered impacted areas located close to the mine (up to 1,200 meters) and 11 sites (Canga and forest), serving as controls, which were at least 7,000 meters from the mine. We sampled four times at each site, twice in each wet and dry season. In each site we installed 60 trap locations, separated by 10–20 m. Each point had three traps that were installed in three categories: ground, understory (0.5 to 2 m height) and canopy (from 5 m height), totaling 20 traps in each stratum. We used live-traps (i.e. Sherman and Tomahawk types), which were open for six consecutive nights, totaling 2,160 trap/nights per sampling time. Each trap contained only a single bait type (chunks of banana, chunks of bacon or peanut butter and sardine mixed), which were alternated over our sample points. We also used pitfalls traps in the 2010 to 2011 period in the areas of forests. It was not possible to install this method in the Canga areas due to the rocky soil. We used 180 60-liter buckets, buried in the ground for the pitfall traps. In each area, six transects of 15 buckets were made, totaling 90 open buckets per area. In each pitfall transect, the buckets were separated from each other by 10 m, connected by a 1 m wall of plastic tarp. The bottoms of all buckets were drilled at the base and had Styrofoam plates placed in the bottom to prevent captured animals’ death by drowning during periods of rain. The pitfall traps remained open for ten consecutive nights, totaling a sampling effort of 900 buckets/site/sample period.

All the types of traps (pitfall and live-traps) were checked at on the following morning. The animals were marked with numbered metal ear tag (the number was specific for each individual) and after this, they were released at the same point of capture.

### Data analysis

The diversity of small mammals, based on data of abundance for species, was compared between the vegetation types studied through an analysis of variance (ANOVA).

We checked for spatial autocorrelation among the records of mammalian species for each site using Mantel tests [17]. For the Mantel tests, we calculated a spatial distance matrix using universal transverse Mercator-14 coordinates (in meters) of each site. The Euclidean distance metric was used to construct distance matrices for space. The Bray-Curtis distance metric was used to construct distance matrix of the records of small mammals per site. The significance of Mantel correlations was evaluated by a permutation test with 9,999 permutations. The analyses were performed in R version 2.15.0 [18] with the package vegan version 2.0–4 for Mantel tests [17].

We used non-metric multidimensional scaling (NMDS; [19]) with diversity and number of individuals of each species of small mammals to compare the similarity between sites in the two vegetation types. We used the Bray-Curtis distance metric based on its high performance in detecting ecological gradients in simulations [20]. NMDS is a distance-based ordination technique that graphically arranges sample units according to their rank order of ecological distance; hence, two points located close together on an ordination plot represent two trapping transects with similar mammalian diversity and abundance. We used the first two axes of the NMDS to evaluate, using ANOVA, if the vegetation type was the variable responsible for the observed similarity. Analyses were performed using Systat software 11.

To determine if there was an impact of mining on small mammal communities, we found the second axis of the NMDS informative to evaluate the similarity in diversity and in a number of individuals in each type of vegetation. We used ANOVA to assess whether there was an effect of the study area (control or impacted) with the second axis NMDS for each vegetation type separately (forest and Canga). We also conducted a simple regression with the distance from the impact (log base 10 transformed) and the second axis of NMDS, to evaluate whether the distance from the mine influenced the diversity and abundance of small mammals. We used a simple linear regression to assess whether species abundance varied with distance from impact site using each transect as a sample unit. We performed analyses using Systat software 11. We calculated richness (S) and diversity index of Shanon-Weiner (H) for each site and year in Diversity of Species Program (DivEs) version 3.0.

To determine if mining affected each species of small mammals individually, we used the distance to the mine as a variable indicator of impact at our 12 sampling sites divided by the total area of Canga and forest. Six sites (Canga and forest) were considered impacted areas located close to the mine (up to 900 meters) with six sites (Canga and forest), serving as controls, which were at least 7,000 meters from the mine. Based on the Mackenzie [21] approach, we constructed a reliable detection history of each small mammal species with each campaign considered as one occasion, which gave us a total of seven occasions. We estimated site occupancy (Ψ) and detection probability (*p*) for the species, with three possible outcomes: (1) the site was occupied and the species was detected (Ψ x *p*); (2) the species was present but not detected (Ψ x [1– *p*]), and (3) the species was not present and therefore was not detected (1– Ψ). The probability was the parameter projected by a maximum likelihood estimation of the proportion of sites occupied (Ψ) during the sample period. We verified that occupancy was closed (i.e., did not change) for the small mammal species using the single-season model. This exercise indicated that the occupancy status for each species was constant throughout the study, allowing us to use closed occupancy models [21]. We constructed a set of candidate models for each species, which were selected by *a priori* hypotheses based on three different approaches: (1) considering occupancy probability and detectability as constant across all sites, (2) considering the variation in occupancy as a function of the distance to the mine, and (3) considering both the variation in occupancy and detectability as a function of the distance of the mine. We estimated detection probabilities by sampling each site on multiple occasions. The occupancy modeling was run in the PRESENCE 9.3 software [22] with 2,000 bootstraps to assess the adjustment fit (p) and the over-dispersion parameter (ĉ). In our assessment of occupancy closure and the factors that influenced occupancy and detection, we ranked all models according to Akaike’s information criterion, or AIC [23]. All models with a ΔAIC value of less than 2 were considered to have equal support. We choose the best model from the entire set of models by using the Akaike weight (w). The Akaike weight provides a straightforward interpretation as of the probabilities of each model’s being the best model in an AIC sense (i.e., by calculating a weighted average of the beta of each covariate and using the AIC weight as the weighting variable).

## Results

We recorded the presence of 28 species, 11 from the Order Didelphimorphia and 17 from the Order Rodentia, with 1,392 captured individuals and 134 recaptures (S1 File). The species that had a higher number of recaptures were *Monodelphis glirina* (68 recaptures), *Marmosa murina* (16 recaptures), *Necromys lasiurus* (12 recaptures), *Oecomys* spp. (nine recaptures) and *Oxymycterus amazonicus* (eight recaptures) (S1 File). The records of the mammalian species were not autocorrelated with respect to space (Mantel's *r* = 0.12, p = 0.14).

The species *Nectomys rattus* and *Necromys lasiurus* were recorded only in the Canga areas and 13 species: *Glironia venusta*, *Metachirus nudicaudatus*, *Monodelphis* “sp. D”, *Monodelphis* aff. *kunsi*, *Philander opossum*, *Hylaeamys megacephalus*, *Neacomys* aff. *paracou*, *Neusticomys ferreirai*, *Oecomys* cf. *bicolor*, *Oecomys* cf. *paricola*, *Oligoryzomys microtis*, *Echimys chrysurus*, *Makalata didelphoides* and *Mesomys stimulax* were recorded only in forest areas (Table 1). Based on the data of relative abundance, the most abundant species in the forest vegetation type were *Oecomys* spp. (223 individuals), representing 32.22% of the species found in the forest. The least abundant species in the forest were *Caluromys philander*, *Glironia venusta*, *Neusticomys ferreirai*, and *Makalata didelphoides* with a single individual caught per species. These rarer species represented 0.14% of captured Forest species (Table 1). The most abundant species in the Canga vegetation type was *Monodelphis glirina*, with 377 individuals, representing 53.93% of the recorded Canga species. The least abundant species in the Canga were *Didelphis marsupialis*, *Euryoryzomys emmonsae* and *Oecomys concolor* with a single individual caught per species and representing 0.14% of captured Canga species (Table 1).

**Table 1:** Number of individuals and relative abundance of non-volant small mammal species recorded at Carajás National Forest in Canga and Forest areas. Legend: RA—Relative abundance (calculated for each vegetation type); NR—No recorded species.

**Order Didelphimorphia**				
**Family Didelphidae**	**Forest**	**RA (%)**	**Canga**	**RA (%)**
*Caluromys philander* (Linnaeus 1758)	01	0.14	02	0.29
*Glironia venusta* Thomas 1912	01	0.14	NR	-
*Didelphis marsupialis* Linnaeus 1758	07	1.01	01	0.14
*Marmosa murina* (Linnaeus 1758)	22	3.18	71	10.16
*Marmosa demerarae* (Thomas 1905)	20	2.89	08	1.14
*Marmosops pinheiroi* Pine 1981	59	8.53	05	0.71
*Metachirus nudicaudatus* (Geoffroy 1803)	06	0.87	NR	-
*Monodelphis glirina* Wagner, 1842	53	7.66	377	53.93
*Monodelphis* “sp. D” (Pine and Handley 2007)	80	11.56	NR	-
*Monodelphis* aff. *kunsi* Pine 1975	02	0.29	NR	-
*Philander opossum* (Linnaeus 1758)	03	0.43	NR	-
**Order Rodentia**				
**Family Cricetidae**	**Forest**	**RA (%)**	**Canga**	**RA (%)**
*Akodon* cf. *cursor* (Winge 1887)	09	1.30	41	5.86
*Euryoryzomys emmonsae* (Musser et al.1998)	91	13.15	01	0.14
*Hylaeamys megacephalus* (Fischer 1814)	06	0.87	NR	-
*Neacomys* aff. *paracou* Voss et al. 2001	37	5.35	NR	-
*Necromys lasiurus* (Lund 1841)	NR	-	81	11.59
*Nectomys rattus* Pelzeln 1883	NR	-	02	0.29
*Neusticomys ferreirai* Percequillo et al. 2005	01	0.14	NR	-
*Oecomys bicolor* Tomes 1860	117	16.90	NR	-
*Oecomys* cf. *paricola* Thomas 1904	02	0.29	NR	-
*Oecomys* cf. *concolor* Wagner 1845	103	14.88	01	0.14
*Oligoryzomys microtis* Allen 1916	03	0.43	NR	-
*Oxymycterus amazonicus* Hershkovitz 1994	24	3.47	92	13.16
*Rhipidomys emiliae* Allen 1916	12	1.73	06	0.86
**Family Echimyidae**	**Forest**	**RA (%)**	**Canga**	**RA (%)**
*Echimys chrysurus* (Zimmermann 1780)	02	0.29	NR	-
*Makalata didelphoides* Desmarest 1817	01	0.14	NR	-
*Mesomys stimulax* Thomas 1911	02	0.29	NR	-
*Proechimys roberti* Thomas 1903	30	4.33	11	1.57
**Total (Number of individuals)**	**692**		**699**	

The richness in the Canga areas ranged from five to nine species, considering both control and impacted areas. On the other hand, the richness of forest areas ranged from 10 to 16 species (Table 2). The diversity index of Shannon-Weiner (H) was lower in the Canga sites (values between 0.32 and 0.76) than in the forest sites (values between 0.71 and 1.03) (Table 2).

**Table 2:** Richness (S) and diversity index of Shannon-Weiner (H) of non-volant small mammals in the sampled sites in the Carajás National Forest.

Area	Sites	H	S
Control Canga	A	0.5058	8
	B	0.4134	6
	C	0.3219	6
	D	0.3508	5
	E	0.5613	8
Impacted Canga	A	0.6294	9
	B	0.6964	8
	C	0.7633	9
	D	0.6769	7
	E	0.57	8
	F	0.6269	9
Control Forest	A	0.8691	10
	B	0.9008	14
	C	0.8081	12
	D	0.7622	12
	E	0.7083	10
	F	0.8314	11
Impacted Forest	A	0.9707	13
	B	1.03	16
	C	0.9041	11
	D	0.9855	13
	E	1	14
	F	0.9306	13

**Fig 2:**
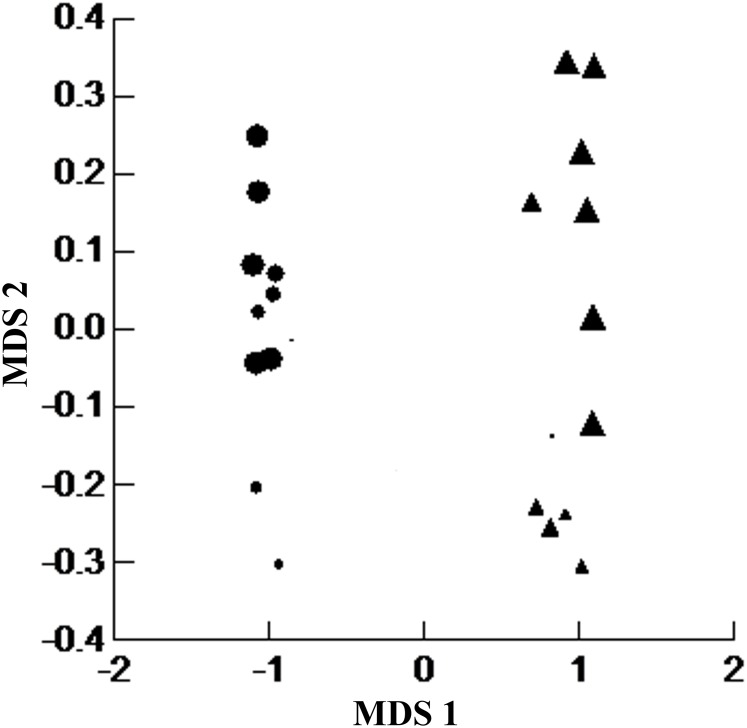
Multidimensional Scaling (NMDS) of the diversity and number of individuals of non-volant small mammals in the Carajás National Forest. The symbols separate the vegetation types (Canga-circles, and forest-triangle) with the size of the symbols indicating the distance from the impact.

The two vegetation types shared 68.42% of the species. The graph generated by the NMDS (stress = 0.044) showed that the first axis clearly separated communities of small mammals by the vegetation type (r^2^ = 0.993, F = 15.50; p<0.0001; Fig 2).

**Fig 3:**
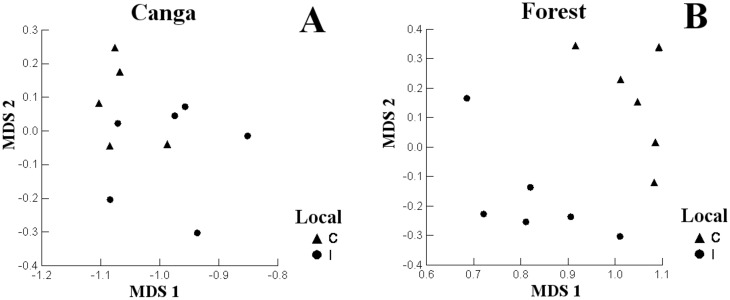
Multidimensional Scaling (NMDS) of the diversity and number of individuals of each species of non-volant small mammals in the areas. (A) Canga closest to the impact and Canga further to the impact; (B) forest closest to the impact and forest further to the impact in the Carajás National Forest. Legend: C = area more near to the impact—triangle; I = area closest to the impact—circles.

The second axis of NMDS was related to Canga, the small mammal communities did not separate based on mining effects (F_1,9_ = 3.866, p = 0.081) (Fig 3A). But in the forest differed significantly between the impacted and control area (F_1,10_ = 10.131, p = 0.010) (Fig 3B).

**Fig 4:**
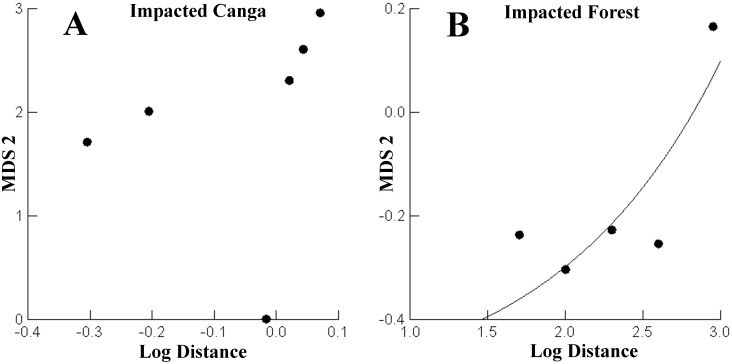
Comparison of the second axis of the NMDS distance with the distance to the mine (impact) in the impacted forest in the Carajás National Forest. Considering each site as an independent sample.

We did not find a significant effect of distance from the mine on small mammal communities for control areas (about 7,000 m from the mine), the second axis of the NMDS did not vary significantly with the distance from the mine, both for the Canga (F_1,4_ = 3.521, R^2^ = 0.540, p = 0.157), and the forest (F_1,4_ = 1.630, R^2^ = 0.290, p = 0.251). For the impacted areas, we also did not find a significant effect of distance from the mine on small mammal communities to Canga (F_1,4_ = 0.249, R^2^ = 0.059, p = 0.644) and the impacted forest (F_1,4_ = 0227; R^2^ = 0.054; p = 0.658). However, we observed that for impacted forest area the relationship between the second axis of the NMDS and the distance to the mine was a non-linear function (MDS2 = -0559 and + 0041 * (0925 * log_distance); R^2^ = 0.576; Fig 4).

**Fig 5:**
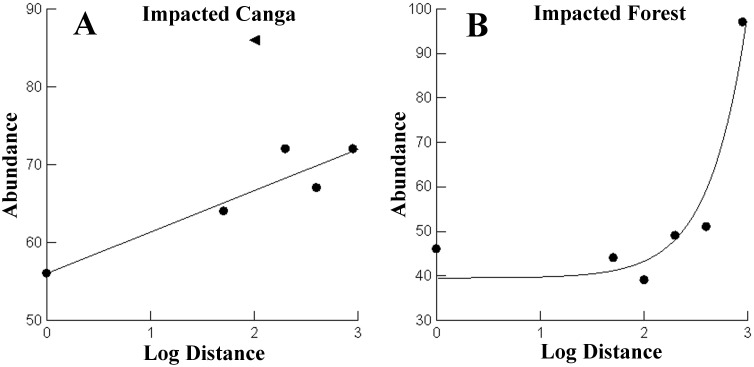
Comparison of the number of individuals of non-volant small mammals with the distance from the mine in the Carajás National Forest, Pará, Brazil. Considering each site as an independent sample. (A) impacted Canga and (B) impacted forest. The function line in A is not considering the transect C marked with a triangle in the graph (see text for explanations).

The number of captured individuals did not vary significantly with respect to distance from the mine to the impacted area of the Canga (F_1,4_ = 2.030, R^2^ = 0.337, p = 0.227). However, when we removed transect C of the analysis, the effect of the distance was significant (F_1,3_ = 19.659; R^2^ = 0.868; p = 0.021) (Fig 5A). The C transect had a positive effect on the number of captured individuals. The number of captured individuals did not differ linearly with distance from the mine to the impacted forest (F_1,4_ = 1.415, R^2^ = 0.261, p = 0.300). However, the nonlinear function explained much of the variation in the number of individuals captured (= Abundance 39 504 + 0015 * E (2.763 * log_dist); R^2^ = 0.929) (Fig 5B).

### Occupancy models

**Fig 6:**
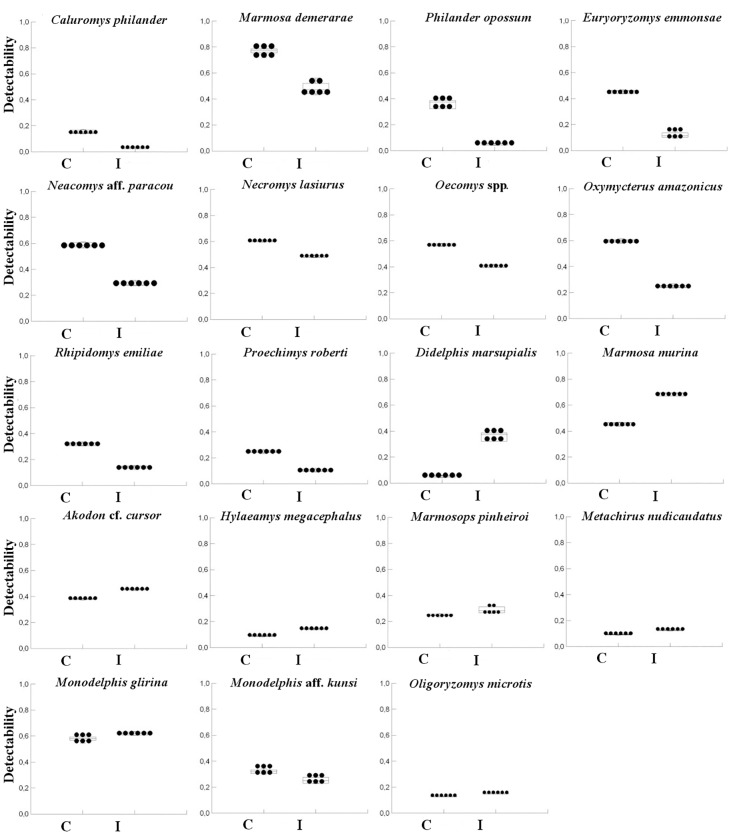
Detectability of species of small mammals on control and impacted areas in the Carajás National Forest, Pará, Brazil. Each point represents one sampled site.

Only detectability was affected by the impact of mining (i.e. distance from the mine). From all species analyzed, 10 species (*C*. *philander*, *M*. *demerarae*, *P*. *opossum*, *E*. *emmonsae*, *N*. aff. *paracou*, *N*. *lasiurus*, *O*. cf. *concolor*, *O*. *amazonicus*, *R*. *emiliae* e *P*. *roberti*) were positively affected by the distance from the mine, with a higher detectability in control areas (i.e. distant about 7,000 m of the mine). However, three species (*D*. *marsupialis*, *M*. *murina*, and *A*. cf. *cursor*) were negatively affected by the distance from the mine, with higher detectability in the impacted areas (i.e. areas near the mine). The remaining species analyzed did not have their detectability affected by the distance from the mine (Fig 6, Table 3).

**Table 3:** Occupancy best models for the non-volant small mammal species in the Carajás National Forest, Pará, Brazil. Covariate: distance to the mine (dist_mine). Ψ = occupancy and *p* = detectability.

Model	AIC	ΔAIC	AICwqt	n° parameters	ĉ
*Caluromys philander*					
Ψ(.); p(dist_mine)	56.32	0	0.87	3	1.06
Ψ(dist_mine);p(dist_mine)	61.56	5.24	0.1	4	1.09
*Marmosa demerarae*					
Ψ(.); p(dist_mine)	62.34	0	0.85	3	1.06
Ψ(dist_mine);p(dist_mine)	67.23	4.89	0.11	4	1.09
*Philander opossum*					
Ψ(.); p(dist_mine)	49.67	0	0.88	3	1.06
Ψ(dist_mine);p(dist_mine)	54.34	4.67	0.09	4	1.09
*Euryoryzomys emmonsae*					
Ψ(.); p(dist_mine)	43.76	0	0.84	3	1.06
Ψ(dist_mine);p(dist_mine)	49.23	5.47	0.12	4	1.09
*Neacomys* aff. *paracou*					
Ψ(.); p(dist_mine)	61.32	0	0.89	3	1.06
Ψ(dist_mine);p(dist_mine)	68.43	7.11	0.08	4	1.09
*Necromys lasiurus*					
Ψ(.); p(dist_mine)	38.78	0	0.91	3	1.06
Ψ(dist_mine);p(dist_mine)	45.23	6.45	0.07	4	1.09
*Oecomys* cf. *concolor*					
Ψ(.); p(dist_mine)	41.34	0	0.82	3	1.06
Ψ(dist_mine);p(dist_mine)	46.54	5.2	0.14	4	1.09
*Oxymycterus amazonicus*					
Ψ(.); p(dist_mine)	58.43	0	0.85	3	1.06
Ψ(dist_mine);p(dist_mine)	62.34	3.91	0.1	4	1.09
*Rhipidomys emiliae*					
Ψ(.); p(dist_mine)	43.21	0	0.81	3	1.06
Ψ(dist_mine);p(dist_mine)	46.54	3.33	0.14	4	1.09
*Proechimys roberti*					
Ψ(.); p(dist_mine)	56.32	0	0.87	3	1.06
Ψ(dist_mine);p(dist_mine)	61.56	5.24	0.1	4	1.09
*Dipelphis marsupialis*					
Ψ(.); p(dist_mine)	69.08	0	0.79	3	1.06
Ψ(dist_mine);p(dist_mine)	73.45	4.37	0.15	4	1.09
*Marmosa murina*					
Ψ(.); p(dist_mine)	41.34	0	0.82	3	1.06
Ψ(dist_mine);p(dist_mine)	46.54	5.2	0.14	4	1.09
*Akodon* cf. *cursor*					
Ψ(.); p(dist_mine)	37.65	0	0.77	3	1.06
Ψ(.);p(.)	40.32	2.67	0.13	2	1.09
*Hylaemys megacephalus*					
Ψ(.); p(.)	33.45	0	0.83	2	1.06
Ψ(.);p(dist_mine)	36.78	3.33	0.15	3	1.09
*Marmosops pinheiroi*					
Ψ(.); p(.)	56.32	0	0.87	2	1.06
Ψ(dist_mine);p(dist_mine)	61.56	3.33	0.1	4	1.09
*Metachirus nudicaudatus*					
Ψ(.); p(.)	43.76	0	0.84	2	1.06
Ψ(dist_mine);p(dist_mine)	49.23	5.47	0.12	4	1.09
*Monodelphis glirina*					
Ψ(.); p(.)	68.45	0	0.89	2	1.06
Ψ(dist_mine);p(.)	74.56	6.11	0.09	3	1.09
*Monodelphis* aff. *kunsi*					
Ψ(.); p(.)	41.34	0	0.9	2	1.06
Ψ(dist_mine);p(.)	47.54	6.2	0.08	3	1.09
*Oligoryzomys microtis*					
Ψ(.); p(.)	38.42	0	0.72	2	1.06
Ψ(dist_mine);p(dist_mine)	41.78	3.36	0.18	4	1.09

## Discussion

We found higher richness and diversity in forest areas. This could be due to differences in the vegetal complexity between the two vegetation types. In fact, Silva [24] and Da Silva et al. [16] found that there are lower plant diversity and a less structural complexity in the Canga than the forest in the Carajás region, Pará, Brazil. Besides that, Canga areas are characterized by a lower availability of resources and soil moisture [16,24]. These results confirm our first hypothesis showing that the diversity of the small mammal community was different between the two vegetation types and species richness was higher in forest areas. Others authors found similar results to our study, where the diversity and abundance of small mammals may be regulated by heterogeneity and diversity habitat type in a locality [25,26].

We recorded species of small mammals exclusively in forest areas, in Canga areas or preferentially in one vegetation type. We found generalist species in the Canga areas [e.g. *Necromys lasiurus*, *Oxymycterus amazonicus* and *Monodelphis glirina*) while other species were more restricted in their use of habitat, so they were recorded more or only in the forest. Our Canga result was similar to the results found for small mammals by Alho et al. [27], Ribeiro and Marinho-Filho [28] and Santos-Filho et al. [29] in the Cerrado biome, where more generalist species occurred only or were more abundant in open habitats. Also, generalist species often display high abundances due to their capacity to spread and persist [30–32]. This high number of generalist individuals may also explain why we found the lowest richness in the Canga, where interspecific competition may be higher, reducing the overall number of species and regulating the community to a more localized level [33–35].

Only in the forest areas, the diversity and abundance of small mammals increased with increasing distance to the mine. These results confirmed our second hypothesis that the negative effect of mining would occur especially in the forest areas, due to a higher vegetal complexity and, therefore more affected because it suffered edge effect [36–38]. Our data contradicts the results found by Malcolm [39], Ochoa [40] and Lambert et al. [41] to small mammals in the Amazonia, where the species had a higher abundance in the disturbed areas. But, these authors studied edge effects on fragmented landscapes, while our study is the first testing the mining impact on small mammal community in the active mine in the Brazilian Amazon. We suggest that there was the escape and accumulation of species from the edge of the mine to interior areas, probably due to vegetation removal, water and air pollution, and noise [42] in areas closest to the mine, that can directly affect the local communities.

In the impacted Canga, one of the sites had a higher abundance of small mammals. Without the effect of this site, we found the abundance higher the further the distance to the mine. The site with high abundance had unique microhabitat features, for example, the presence of water courses and gallery forest within the dry vegetation of Canga (personal obs.). Even in the Canga, where there is not an edge effect, the small mammals also appeared to be moving away from the closest sites of the mine to interior areas. This fact can have occurred probably because of impacts, as we cited above, which according Reis [42] may be irreversible.

The species detectability analysis showed that most species were affected by the distance from the mine, i.e., the farther away from the mining activities the higher the detectability. *Euryoryzomys emmonsae*, *Oecomys* spp., *Marmosa demerarae* and *Rhipidomys emiliae* were listed as being forest-interior species [41,43–47] and for Malcolm [44] as being a transitional forest species, similar our results, which were more detected in control areas. Besides that Figueiredo and Fernandez [48] showed that *Oecomys* spp. may disappear from impacted areas by fire, as well as our records of these species only in forests and more in further areas of mining impact. Alho [45], Mares et al. [49] and Lacher and Alho [50] confirmed that *Rhipidomys* spp. and *Neacomys* spp. in Cerrado are known for using gallery forest and avoid open habitats, the similar our results in Canga. Some generalist/opportunist species were more detected in the control areas: *Philander opossum* and *Proechimys roberti*. These species may forage in one habitat type while nesting in others (e.g., Malcolm [39]). It is important to highlight that *Glironia venusta* is an arboreal marsupial known as rare or vulnerable [51–52]. Our unique voucher specimen was the 11^th^ of the world and the first record to CNF [53], captured in the edge of the mine (0 m distance) where the vegetation was already removed by mining activity.

On the other hand, some species were negatively affected by the distance to the mine (in particular *D*. *marsupialis* and *M*. *murina*). The generalist/opportunist species are more detected in closest areas of the mine (e.g., *Didelphis marsupialis*, *Marmosa murina*, *Akodon* cf. *cursor*). On other studies, these species also were negatively affected by other impact types (e.g. fire, fragmentation) [41,46–47]. Lambert et al. [41] obtained evidence of increased generalist resource abundance in disturbed areas, but these authors also highlighted that the relationship of small mammals to resources may not be a direct one [41]. There are others factors that can favor the generalist/opportunist species: decrease the number of predators [54] and a decrease of interspecific competition [55–56]. However, we did not test these factors to understand the response of small mammals to disturbances of mining.

In summary, this study was the first analyzing the mining impact on non-volant small mammal species in the Brazilian Amazon. It is important to highlight the conservation of the CNF, where there is a unique phytophysionomic characteristic in the world (i.e. Canga). Besides that, in the CNF we recorded species of small mammals exclusively or preferentially in one vegetation type. The majority of these recorded species showed to be more sensitive to the impact mining that is a strong activity in the CNF. So, it is necessary to be made a long-term monitoring trying to mitigate the irreversible impacts of mining. The future studies should be not only evaluating impacts on ecological parameters of populations but also with other focuses, for example, analyzing the contamination on organisms by liquid and solid wastes produced by mining.

## Supporting Information

S1 File Collected data of small mammal community in the Carajás National Forest from January 2010 to August 2011. Legend: Canga 1 = Control Canga; Canga 2 = Impacted Canga; Floresta 1 = Control Canga; Floresta 2 = Impacted Forest. SP = Initials of each captured species. 0 = It is not a recapture and 1 = It is a recapture.
(DOCX)
