# Supplementary material for: Diversity and Impacts of Mining on the Non-Volant Small Mammal Communities of Two Vegetation Types in the Brazilian Amazon
Source: PLoS One. 2016 Nov 28;11(11):e0167266. doi: 10.1371/journal.pone.0167266 (PMC5125694; doi:10.1371/journal.pone.0167266)
Supplement: S1 File — Legend: Canga 1 = Control Canga; Canga 2 = Impacted Canga; Floresta 1 = Control Canga; Floresta 2 = Impacted Forest. SP = Initials of each captured species. 0 = It is not a recapture and 1 = It is a recapture. (DOCX) [file pone.0167266.s001.docx]

**S1 Table**

| AREA | SITE | POINT | CAPTURE DATE | CAMPAIGN | GENUS | SPECIES | SP | EAR TAG | SEX | RECAPTURE |
| --- | --- | --- | --- | --- | --- | --- | --- | --- | --- | --- |
| CANGA 2 | B | 40 | 16/jan/10 | 1 | *Akodon* | *aff. cursor* | AKCU | 345 | F | 0 |
| CANGA 2 | C | 38 | 16/jan/10 | 1 | *Akodon* | *aff. cursor* | AKCU | . | M | 0 |
| CANGA 2 | B | 30 | 17/jan/10 | 1 | *Akodon* | *aff. cursor* | AKCU | 382 | M | 0 |
| CANGA 2 | C | 36 | 17/jan/10 | 1 | *Akodon* | *aff. cursor* | AKCU | 384 | F | 0 |
| CANGA 2 | C | 22 | 17/jan/10 | 1 | *Akodon* | *aff. cursor* | AKCU | 386 | M | 0 |
| CANGA 2 | E | 17 | 17/jan/10 | 1 | *Akodon* | *aff. cursor* | AKCU | 392 | M | 0 |
| CANGA 2 | C | 22 | 18/jan/10 | 1 | *Akodon* | *aff. cursor* | AKCU | 276 | M | 1 |
| CANGA 2 | C | 44 | 18/jan/10 | 1 | *Akodon* | *aff. cursor* | AKCU | . | F | 0 |
| CANGA 2 | C | 38 | 18/jan/10 | 1 | *Akodon* | *aff. cursor* | AKCU | 374 | M | 0 |
| CANGA 2 | A | 7 | 19/jan/10 | 1 | *Akodon* | *aff. cursor* | AKCU | 394 | M | 1 |
| CANGA 2 | A | 19 | 19/jan/10 | 1 | *Akodon* | *aff. cursor* | AKCU | 402 | F | 0 |
| CANGA 2 | B | 22 | 19/jan/10 | 1 | *Akodon* | *aff. cursor* | AKCU | . | M | 0 |
| CANGA 2 | B | 18 | 19/jan/10 | 1 | *Akodon* | *aff. cursor* | AKCU | 401 | F | 0 |
| CANGA 2 | C | 54 | 19/jan/10 | 1 | *Akodon* | *aff. cursor* | AKCU | 400 | F | 0 |
| CANGA 2 | C | 38 | 19/jan/10 | 1 | *Akodon* | *aff. cursor* | AKCU | 374 | F | 1 |
| CANGA 2 | C | 34 | 19/jan/10 | 1 | *Akodon* | *aff. cursor* | AKCU | . | F | 0 |
| CANGA 2 | C | 22 | 19/jan/10 | 1 | *Akodon* | *aff. cursor* | AKCU | 371 | M | 0 |
| CANGA 2 | C | 16 | 19/jan/10 | 1 | *Akodon* | *aff. cursor* | AKCU | 397 | M | 0 |
| CANGA 2 | C | 12 | 19/jan/10 | 1 | *Akodon* | *aff. cursor* | AKCU | 369 | F | 0 |
| CANGA 2 | E | 17 | 19/jan/10 | 1 | *Akodon* | *aff. cursor* | AKCU | . | M | 0 |
| CANGA 2 | G | 41 | 20/jan/10 | 1 | *Akodon* | *aff. cursor* | AKCU | 411 | F | 0 |
| CANGA 2 | B | 16 | 21/jan/10 | 1 | *Akodon* | *aff. cursor* | AKCU | 418 | M | . |
| CANGA 2 | B | 36 | 21/jan/10 | 1 | *Akodon* | *aff. cursor* | AKCU | . | F | 0 |
| CANGA 2 | B | 52 | 21/jan/10 | 1 | *Akodon* | *aff. cursor* | AKCU | 425 | M | 0 |
| CANGA 2 | B | 26 | 21/jan/10 | 1 | *Akodon* | *aff. cursor* | AKCU | 423 | F | 0 |
| CANGA 2 | D | 49 | 21/jan/10 | 1 | *Akodon* | *aff. cursor* | AKCU | . | F | 0 |
| CANGA 2 | E | 10 | 21/jan/10 | 1 | *Akodon* | *aff. cursor* | AKCU | 449 | F | 0 |
| CANGA 1 | C | 36 | 25/jan/10 | 1 | *Akodon* | *aff. cursor* | AKCU | . | F | 0 |
| CANGA 1 | A | 14 | 27/jan/10 | 1 | *Akodon* | *aff. cursor* | AKCU | 572 | F | 0 |
| FLORESTA 2 | B | 14 | 7-mar-10 | 1 | *Akodon* | *aff. cursor* | AKCU | . | M | 0 |
| CANGA 2 | B | 33 | 18/jan/10 | 1 | *Caluromys* | *philander* | CAPH | . | M | 0 |
| CANGA 1 | E | 1 | 23-jan-10 | 1 | *Didelphis* | *marsupialis* | DIMA | . | F | 0 |
| FLORESTA 2 | C | 49 | 02/feb/2010 | 1 | *Didelphis* | *marsupialis* | DIMA | 4826 | M | 0 |
| FLORESTA 2 | E | 5 | 8-mar-10 | 1 | *Didelphis* | *marsupialis* | DIMA | 4855 | M | 0 |
| FLORESTA 2 | A | 28 | 02/feb/2010 | 1 | *Euryoryzomys* | *emmonsae* | EUEM | . |  | 0 |
| FLORESTA 1 | D | 9 | 25/feb/2010 | 1 | *Euryoryzomys* | *emmonsae* | EUEM | . | . | 0 |
| FLORESTA 1 | B | 2 | 1-mar-10 | 1 | *Euryoryzomys* | *emmonsae* | EUEM | 606 | F | 0 |
| FLORESTA 1 | B | 2 | 2-mar-10 | 1 | *Euryoryzomys* | *emmonsae* | EUEM | 608 | F | 0 |
| FLORESTA 1 | B | 3 | 3-mar-10 | 1 | *Euryoryzomys* | *emmonsae* | EUEM | . |  | 0 |
| FLORESTA 1 | B | 3 | 4-mar-10 | 1 | *Euryoryzomys* | *emmonsae* | EUEM | 612 | M | 0 |
| FLORESTA 1 | B | 3 | 5-mar-10 | 1 | *Euryoryzomys* | *emmonsae* | EUEM | . | F | 0 |
| FLORESTA 1 | A | 13 | 6-mar-10 | 1 | *Euryoryzomys* | *emmonsae* | EUEM | 620 | M | 0 |
| FLORESTA 1 | A | 13 | 7-mar-10 | 1 | *Euryoryzomys* | *emmonsae* | EUEM | 646 | M | 0 |
| FLORESTA 2 | B | 9 | 6-mar-10 | 1 | *Euryoryzomys* | *emmonsae* | EUEM | . | F | 0 |
| FLORESTA 2 | G | 11 | 6-mar-10 | 1 | *Euryoryzomys* | *emmonsae* | EUEM | 660 | M | 0 |
| FLORESTA 2 | C | 7 | 8-mar-10 | 1 | *Euryoryzomys* | *emmonsae* | EUEM | 682 | F | 0 |
| FLORESTA 2 | B | 6 | 8-mar-10 | 1 | *Euryoryzomys* | *emmonsae* | EUEM | 684 | F | 0 |
| FLORESTA 2 | E | 11 | 8-mar-10 | 1 | *Euryoryzomys* | *emmonsae* | EUEM | 686 | M | 0 |
| FLORESTA 2 | A | 4 | 10-mar-10 | 1 | *Euryoryzomys* | *emmonsae* | EUEM | 702 | F | 0 |
| FLORESTA 2 | G | 15 | 11-mar-10 | 1 | *Euryoryzomys* | *emmonsae* | EUEM | 712 | M | 0 |
| FLORESTA 2 | G | 1 | 11-mar-10 | 1 | *Euryoryzomys* | *emmonsae* | EUEM | 716 | F | 0 |
| FLORESTA 2 | A | 2 | 11-mar-10 | 1 | *Euryoryzomys* | *emmonsae* | EUEM | 718 | F | 0 |
| FLORESTA 2 | G | 11 | 11-mar-10 | 1 | *Euryoryzomys* | *emmonsae* | EUEM | 720 | F | 0 |
| FLORESTA 2 | E | 1 | 11-mar-10 | 1 | *Euryoryzomys* | *emmonsae* | EUEM | 724 | M | 0 |
| FLORESTA 2 | E | 6 | 14-mar-10 | 1 | *Euryoryzomys* | *emmonsae* | EUEM | 734 | M | 0 |
| FLORESTA 1 | B | 6 | 28-fev-10 | 1 | *Hylaeamys* | *megacephalus* | HYME | . | F | 0 |
| CANGA 2 | A | 22 | 16-jan-10 | 1 | *Marmosa* | *murina* | MAMU | 342 | M | 0 |
| CANGA 2 | A | 30 | 16-jan-10 | 1 | *Marmosa* | *murina* | MAMU | 343 | F | 0 |
| CANGA 2 | A | 46 | 16-jan-10 | 1 | *Marmosa* | *murina* | MAMU | 345 | F | 0 |
| CANGA 2 | B | 25 | 16-jan-10 | 1 | *Marmosa* | *murina* | MAMU | 341 | F | 0 |
| CANGA 2 | B | 49 | 16-jan-10 | 1 | *Marmosa* | *murina* | MAMU | 268 | F | 1 |
| CANGA 2 | C | 29 | 16-jan-10 | 1 | *Marmosa* | *murina* | MAMU | 186 | F | 1 |
| CANGA 2 | C | 26 | 16-jan-10 | 1 | *Marmosa* | *murina* | MAMU | 346 | F | 0 |
| CANGA 2 | C | 30 | 16-jan-10 | 1 | *Marmosa* | *murina* | MAMU | 155 | F | 1 |
| CANGA 2 | D | 20 | 16-jan-10 | 1 | *Marmosa* | *murina* | MAMU | 355 | F | 0 |
| CANGA 2 | E | 39 | 16-jan-10 | 1 | *Marmosa* | *murina* | MAMU | 353 | M | 0 |
| CANGA 2 | E | 11 | 16-jan-10 | 1 | *Marmosa* | *murina* | MAMU | 327 | M | 0 |
| CANGA 2 | A | 16 | 17-jan-10 | 1 | *Marmosa* | *murina* | MAMU | . | F | 0 |
| CANGA 2 | A | 33 | 17-jan-10 | 1 | *Marmosa* | *murina* | MAMU | 380 | F | 0 |
| CANGA 2 | D | 47 | 17-jan-10 | 1 | *Marmosa* | *murina* | MAMU | 376 | M | 0 |
| CANGA 2 | B | 16 | 17-jan-10 | 1 | *Marmosa* | *murina* | MAMU | . | F | 0 |
| CANGA 2 | B | 55 | 17-jan-10 | 1 | *Marmosa* | *murina* | MAMU | 381 | M | 0 |
| CANGA 2 | B | 47 | 17-jan-10 | 1 | *Marmosa* | *murina* | MAMU | 268 | M | 1 |
| CANGA 2 | E | 30 | 17-jan-10 | 1 | *Marmosa* | *murina* | MAMU | 390 | F | 0 |
| CANGA 2 | E | 1 | 17-jan-10 | 1 | *Marmosa* | *murina* | MAMU | 391 | M | 0 |
| CANGA 2 | C | 32 | 17-jan-10 | 1 | *Marmosa* | *murina* | MAMU | 186 | F | 1 |
| CANGA 2 | C | 16 | 17-jan-10 | 1 | *Marmosa* | *murina* | MAMU | . | M | 0 |
| CANGA 2 | B | 18 | 18-jan-10 | 1 | *Marmosa* | *murina* | MAMU | 362 | F | 0 |
| CANGA 2 | B | 55 | 18-jan-10 | 1 | *Marmosa* | *murina* | MAMU | . |  | 1 |
| CANGA 2 | B | 19 | 18-jan-10 | 1 | *Marmosa* | *murina* | MAMU | 341 | F | 1 |
| CANGA 2 | C | 23 | 18-jan-10 | 1 | *Marmosa* | *murina* | MAMU | . | F | 0 |
| CANGA 2 | D | 25 | 18-jan-10 | 1 | *Marmosa* | *murina* | MAMU | 361 | F | 0 |
| CANGA 2 | D | 18 | 18-jan-10 | 1 | *Marmosa* | *murina* | MAMU | 368 | F | 0 |
| CANGA 2 | G | 48 | 18-jan-10 | 1 | *Marmosa* | *murina* | MAMU | 364 | M | 0 |
| CANGA 2 | A | 30 | 19-jan-10 | 1 | *Marmosa* | *murina* | MAMU | 343 | F | 1 |
| CANGA 2 | B | 13 | 19-jan-10 | 1 | *Marmosa* | *murina* | MAMU | 399 | M | 0 |
| CANGA 2 | B | 2 | 19-jan-10 | 1 | *Marmosa* | *murina* | MAMU | 403 | M | 0 |
| CANGA 2 | G | 34 | 19-jan-10 | 1 | *Marmosa* | *murina* | MAMU | . | M | 0 |
| CANGA 2 | A | 42 | 20-jan-10 | 1 | *Marmosa* | *murina* | MAMU | 406 | F | 0 |
| CANGA 2 | A | 45 | 20-jan-10 | 1 | *Marmosa* | *murina* | MAMU | 268 | F | 1 |
| CANGA 2 | B | 23 | 21-jan-10 | 1 | *Marmosa* | *murina* | MAMU | 422 | F | 0 |
| CANGA 2 | B | 19 | 21-jan-10 | 1 | *Marmosa* | *murina* | MAMU | 426 | F | 0 |
| CANGA 2 | B | 37 | 21-jan-10 | 1 | *Marmosa* | *murina* | MAMU | 155 | F | 1 |
| CANGA 2 | D | 28 | 21-jan-10 | 1 | *Marmosa* | *murina* | MAMU | 345 | F | 0 |
| CANGA 2 | E | 35 | 21-jan-10 | 1 | *Marmosa* | *murina* | MAMU | 429 | F | 0 |
| CANGA 1 | A | 35 | 27-jan-10 | 1 | *Marmosa* | *murina* | MAMU | 532 | F | 0 |
| FLORESTA 2 | G | 4 | 31-jan-10 | 1 | *Marmosa* | *murina* | MAMU | . | M | 0 |
| FLORESTA 2 | B | 25 | 01-feb-2010 | 1 | *Marmosa* | *murina* | MAMU | 580 | F | 0 |
| FLORESTA 1 | A | 11 | 07-feb-2010 | 1 | *Marmosa* | *murina* | MAMU | . |  | 0 |
| FLORESTA 1 | C | 9 | 1-mar-10 | 1 | *Marmosa* | *murina* | MAMU | . | F | 0 |
| FLORESTA 2 | E | 9 | 10-mar-10 | 1 | *Marmosa* | *murina* | MAMU | 700 | . | 0 |
| FLORESTA 2 | E | 6 | 14-mar-10 | 1 | *Marmosa* | *murina* | MAMU | 744 | M | 0 |
| FLORESTA 1 | B | 10 | 26-fev-10 | 1 | *Marmosops* | *pinheiroi* | MAPI | . | F | 0 |
| FLORESTA 1 | A | 6 | 26-fev-10 | 1 | *Marmosops* | *pinheiroi* | MAPI | . | F | 0 |
| FLORESTA 1 | D | 4 | 27-fev-10 | 1 | *Marmosops* | *pinheiroi* | MAPI | 592 | F | 0 |
| FLORESTA 1 | E | 7 | 28-fev-10 | 1 | *Marmosops* | *pinheiroi* | MAPI | . | M | 0 |
| FLORESTA 1 | D | 12 | 28-fev-10 | 1 | *Marmosops* | *pinheiroi* | MAPI | . | F | 0 |
| FLORESTA 1 | G | 5 | 1-mar-10 | 1 | *Marmosops* | *pinheiroi* | MAPI | . | M | 0 |
| FLORESTA 1 | E | 15 | 4-mar-10 | 1 | *Marmosops* | *pinheiroi* | MAPI | 650 | M | 0 |
| FLORESTA 2 | B | 1 | 6-mar-10 | 1 | *Marmosops* | *pinheiroi* | MAPI | 652 | F | 0 |
| FLORESTA 2 | B | 15 | 7-mar-10 | 1 | *Marmosops* | *pinheiroi* | MAPI | . |  | 0 |
| FLORESTA 2 | C | 3 | 8-mar-10 | 1 | *Marmosops* | *pinheiroi* | MAPI | 678 | M | 0 |
| FLORESTA 2 | C | 4 | 8-mar-10 | 1 | *Marmosops* | *pinheiroi* | MAPI | 680 | M | 0 |
| FLORESTA 2 | E | 15 | 8-mar-10 | 1 | *Marmosops* | *pinheiroi* | MAPI | 688 | M | 0 |
| FLORESTA 2 | A | 9 | 9-mar-10 | 1 | *Marmosops* | *pinheiroi* | MAPI | 696 | F | 0 |
| FLORESTA 2 | A | 15 | 11-mar-10 | 1 | *Marmosops* | *pinheiroi* | MAPI | 726 | F | 0 |
| FLORESTA 2 | A | 1 | 2-fev-10 | 1 | *Metachirus* | *nudicaudatus* | MENU | . | F | 0 |
| FLORESTA 1 | D | 13 | 28-fev-10 | 1 | *Metachirus* | *nudicaudatus* | MENU | . | F | 0 |
| CANGA 2 | D | 49 | 18-jan-10 | 1 | *Marmosa* | *demerarae* | MADE | 360 | F | 0 |
| CANGA 2 | A | 50 | 19-jan-10 | 1 | *Marmosa* | *demerarae* | MADE | . |  | 0 |
| CANGA 2 | C | 23 | 19-jan-10 | 1 | *Marmosa* | *demerarae* | MADE | 408 | M | 0 |
| CANGA 2 | E | 31 | 19-jan-10 | 1 | *Marmosa* | *demerarae* | MADE | 395 | M | 0 |
| FLORESTA 2 | G | 23 | 31-jan-10 | 1 | *Marmosa* | *demerarae* | MADE | 574 | F | 0 |
| FLORESTA 2 | A | 4 | 01/feb/2010 | 1 | *Marmosa* | *demerarae* | MADE | . | F | 0 |
| FLORESTA 2 | E | 35 | 02/feb/2010 | 1 | *Marmosa* | *demerarae* | MADE | 578 | M | 0 |
| FLORESTA 2 | E | 41 | 02/feb/2010 | 1 | *Marmosa* | *demerarae* | MADE | 576 | M | 0 |
| FLORESTA 1 | C | 26 | 06/feb/2010 | 1 | *Marmosa* | *demerarae* | MADE | . | F | 0 |
| FLORESTA 1 | E | 41 | 09/feb/2010 | 1 | *Marmosa* | *demerarae* | MADE | 584 | M | 0 |
| CANGA 2 | A | 16 | 16/jan/10 | 1 | *Monodelphis* | *glirina* | MOGL | . | F | 0 |
| CANGA 2 | B | 45 | 16/jan/10 | 1 | *Monodelphis* | *glirina* | MOGL | 347 | F | 0 |
| CANGA 2 | C | 48 | 16/jan/10 | 1 | *Monodelphis* | *glirina* | MOGL | 301 | F | 1 |
| CANGA 2 | C | 22 | 16/jan/10 | 1 | *Monodelphis* | *glirina* | MOGL | 349 | M | 0 |
| CANGA 2 | D | 8 | 16/jan/10 | 1 | *Monodelphis* | *glirina* | MOGL | 356 | F | 0 |
| CANGA 2 | D | 6 | 16/jan/10 | 1 | *Monodelphis* | *glirina* | MOGL | 282 | F | 1 |
| CANGA 2 | G | 29 | 16/jan/10 | 1 | *Monodelphis* | *glirina* | MOGL | 253 | F | 1 |
| CANGA 2 | E | 5 | 16/jan/10 | 1 | *Monodelphis* | *glirina* | MOGL | 233 | F | 1 |
| CANGA 2 | G | 51 | 16/jan/10 | 1 | *Monodelphis* | *glirina* | MOGL | 357 | M | 0 |
| CANGA 2 | G | 23 | 16/jan/10 | 1 | *Monodelphis* | *glirina* | MOGL | 358 | F | 0 |
| CANGA 2 | G | 57 | 16/jan/10 | 1 | *Monodelphis* | *glirina* | MOGL | 359 | M | 0 |
| CANGA 2 | E | 3 | 16/jan/10 | 1 | *Monodelphis* | *glirina* | MOGL | 352 | F | 0 |
| CANGA 2 | G | 15 | 16/jan/10 | 1 | *Monodelphis* | *glirina* | MOGL | . | M | 0 |
| CANGA 2 | E | 4 | 16/jan/10 | 1 | *Monodelphis* | *glirina* | MOGL | . |  | 0 |
| CANGA 2 | A | 19 | 17/jan/10 | 1 | *Monodelphis* | *glirina* | MOGL | . | F | 0 |
| CANGA 2 | A | 6 | 17/jan/10 | 1 | *Monodelphis* | *glirina* | MOGL | . |  | 0 |
| CANGA 2 | B | 34 | 17/jan/10 | 1 | *Monodelphis* | *glirina* | MOGL | . | M | 0 |
| CANGA 2 | C | 34 | 17/jan/10 | 1 | *Monodelphis* | *glirina* | MOGL | 383 | F | 0 |
| CANGA 2 | G | 45 | 17/jan/10 | 1 | *Monodelphis* | *glirina* | MOGL | 385 | M | 0 |
| CANGA 2 | E | 44 | 17/jan/10 | 1 | *Monodelphis* | *glirina* | MOGL | 388 | F | 0 |
| CANGA 2 | E | 10 | 17/jan/10 | 1 | *Monodelphis* | *glirina* | MOGL | 389 | M | 0 |
| CANGA 2 | A | 35 | 18/jan/10 | 1 | *Monodelphis* | *glirina* | MOGL | 367 | F | 0 |
| CANGA 2 | B | 47 | 18/jan/10 | 1 | *Monodelphis* | *glirina* | MOGL | . | M | 0 |
| CANGA 2 | E | 8 | 18/jan/10 | 1 | *Monodelphis* | *glirina* | MOGL | 373 | M | 0 |
| CANGA 2 | D | 31 | 18/jan/10 | 1 | *Monodelphis* | *glirina* | MOGL | 366 | M | 0 |
| CANGA 2 | G | 33 | 18/jan/10 | 1 | *Monodelphis* | *glirina* | MOGL | . | F | 0 |
| CANGA 2 | D | 6 | 19/jan/10 | 1 | *Monodelphis* | *glirina* | MOGL | 484 | F | 0 |
| CANGA 2 | D | 13 | 19/jan/10 | 1 | *Monodelphis* | *glirina* | MOGL | 396 | M | 0 |
| CANGA 2 | D | 31 | 19/jan/10 | 1 | *Monodelphis* | *glirina* | MOGL | 405 | F | 0 |
| CANGA 2 | B | 5 | 19/jan/10 | 1 | *Monodelphis* | *glirina* | MOGL | 398 | F | 0 |
| CANGA 2 | G | 27 | 19/jan/10 | 1 | *Monodelphis* | *glirina* | MOGL | 253 | F | 1 |
| CANGA 2 | E | 6 | 19/jan/10 | 1 | *Monodelphis* | *glirina* | MOGL | . | M | 0 |
| CANGA 2 | A | 5 | 20/jan/10 | 1 | *Monodelphis* | *glirina* | MOGL | 275 | M | 1 |
| CANGA 2 | A | 29 | 20/jan/10 | 1 | *Monodelphis* | *glirina* | MOGL | 413 | M | 0 |
| CANGA 2 | C | 6 | 20/jan/10 | 1 | *Monodelphis* | *glirina* | MOGL | 414 | F | 0 |
| CANGA 2 | D | 19 | 20/jan/10 | 1 | *Monodelphis* | *glirina* | MOGL | 412 | M | 0 |
| CANGA 2 | E | 14 | 20/jan/10 | 1 | *Monodelphis* | *glirina* | MOGL | 409 | F | 0 |
| CANGA 2 | A | 15 | 21/jan/10 | 1 | *Monodelphis* | *glirina* | MOGL | 416 | F | 0 |
| CANGA 2 | A | 19 | 21/jan/10 | 1 | *Monodelphis* | *glirina* | MOGL | 375 | M | 0 |
| CANGA 2 | C | 54 | 21/jan/10 | 1 | *Monodelphis* | *glirina* | MOGL | 421 | F | 0 |
| CANGA 2 | D | 6 | 21/jan/10 | 1 | *Monodelphis* | *glirina* | MOGL | 436 | M | 0 |
| CANGA 2 | D | 2 | 21/jan/10 | 1 | *Monodelphis* | *glirina* | MOGL | . | F | 0 |
| CANGA 2 | D | 40 | 21/jan/10 | 1 | *Monodelphis* | *glirina* | MOGL | 432 | F | 0 |
| CANGA 2 | G | 29 | 21/jan/10 | 1 | *Monodelphis* | *glirina* | MOGL | 424 | M | 0 |
| CANGA 2 | G | 24 | 21/jan/10 | 1 | *Monodelphis* | *glirina* | MOGL | 427 | F | 0 |
| CANGA 2 | G | 13 | 21/jan/10 | 1 | *Monodelphis* | *glirina* | MOGL | 419 | M | 0 |
| CANGA 2 | G | 57 | 21/jan/10 | 1 | *Monodelphis* | *glirina* | MOGL | . | M | 0 |
| CANGA 2 | E | 27 | 21/jan/10 | 1 | *Monodelphis* | *glirina* | MOGL | . | M | 0 |
| CANGA 1 | A | 46 | 23/jan/10 | 1 | *Monodelphis* | *glirina* | MOGL | 496 | M | 0 |
| CANGA 1 | A | 28 | 23/jan/10 | 1 | *Monodelphis* | *glirina* | MOGL | 25 | F | 1 |
| CANGA 1 | A | 14 | 23/jan/10 | 1 | *Monodelphis* | *glirina* | MOGL | 437 | M | 0 |
| CANGA 1 | A | 20 | 23/jan/10 | 1 | *Monodelphis* | *glirina* | MOGL | 123 | F | 1 |
| CANGA 1 | A | 22 | 23/jan/10 | 1 | *Monodelphis* | *glirina* | MOGL | 479 | M | 0 |
| CANGA 1 | A | 15 | 23/jan/10 | 1 | *Monodelphis* | *glirina* | MOGL | 438 | F | 0 |
| CANGA 1 | A | 39 | 23/jan/10 | 1 | *Monodelphis* | *glirina* | MOGL | 482 | M | 0 |
| CANGA 1 | A | 0 | 23/jan/10 | 1 | *Monodelphis* | *glirina* | MOGL | 47 | F | 1 |
| CANGA 1 | B | 29 | 23/jan/10 | 1 | *Monodelphis* | *glirina* | MOGL | 480 | F | 0 |
| CANGA 1 | B | 20 | 23/jan/10 | 1 | *Monodelphis* | *glirina* | MOGL | . |  | 0 |
| CANGA 1 | B | 26 | 23/jan/10 | 1 | *Monodelphis* | *glirina* | MOGL | 46 | F | 1 |
| CANGA 1 | B | 4 | 23/jan/10 | 1 | *Monodelphis* | *glirina* | MOGL | 30 | F | 1 |
| CANGA 1 | B | 6 | 23/jan/10 | 1 | *Monodelphis* | *glirina* | MOGL | . | F | 0 |
| CANGA 1 | B | 12 | 23/jan/10 | 1 | *Monodelphis* | *glirina* | MOGL | 439 | M | 0 |
| CANGA 1 | C | 13 | 23/jan/10 | 1 | *Monodelphis* | *glirina* | MOGL | 91 | F | 1 |
| CANGA 1 | C | 16 | 23/jan/10 | 1 | *Monodelphis* | *glirina* | MOGL | 447 | M | 1 |
| CANGA 1 | E | 24 | 23/jan/10 | 1 | *Monodelphis* | *glirina* | MOGL | 445 | F | 0 |
| CANGA 1 | E | 21 | 23/jan/10 | 1 | *Monodelphis* | *glirina* | MOGL | 454 | M | 0 |
| CANGA 1 | E | 16 | 23/jan/10 | 1 | *Monodelphis* | *glirina* | MOGL | 511 | M | 1 |
| CANGA 1 | E | 18 | 23/jan/10 | 1 | *Monodelphis* | *glirina* | MOGL | 477 | M | 0 |
| CANGA 1 | D | 45 | 23/jan/10 | 1 | *Monodelphis* | *glirina* | MOGL | 452 | M | 0 |
| CANGA 1 | C | 22 | 24/jan/10 | 1 | *Monodelphis* | *glirina* | MOGL | 448 |  | 0 |
| CANGA 1 | B | 1 | 24/jan/10 | 1 | *Monodelphis* | *glirina* | MOGL | . |  | 0 |
| CANGA 1 | A | 26 | 24/jan/10 | 1 | *Monodelphis* | *glirina* | MOGL | 442 | M | 0 |
| CANGA 1 | A | 42 | 24/jan/10 | 1 | *Monodelphis* | *glirina* | MOGL | . | M | . |
| CANGA 1 | A | 28 | 24/jan/10 | 1 | *Monodelphis* | *glirina* | MOGL | 20 | F | 1 |
| CANGA 1 | A | 18 | 24/jan/10 | 1 | *Monodelphis* | *glirina* | MOGL | 446 | M | 0 |
| CANGA 1 | D | 39 | 24/jan/10 | 1 | *Monodelphis* | *glirina* | MOGL | 441 | F | 0 |
| CANGA 1 | E | 6 | 24/jan/10 | 1 | *Monodelphis* | *glirina* | MOGL | 443 | F | 0 |
| CANGA 1 | C | 4 | 25/jan/10 | 1 | *Monodelphis* | *glirina* | MOGL | 488 | M | 0 |
| CANGA 1 | C | 16 | 25/jan/10 | 1 | *Monodelphis* | *glirina* | MOGL | 490 | F | 0 |
| CANGA 1 | C | 26 | 25/jan/10 | 1 | *Monodelphis* | *glirina* | MOGL | 492 | M | 0 |
| CANGA 1 | E | 6 | 25/jan/10 | 1 | *Monodelphis* | *glirina* | MOGL | 186 | M | 1 |
| CANGA 1 | E | 16 | 25/jan/10 | 1 | *Monodelphis* | *glirina* | MOGL | 494 | F | 0 |
| CANGA 1 | E | 18 | 25/jan/10 | 1 | *Monodelphis* | *glirina* | MOGL | 496 | M | 0 |
| CANGA 1 | A | 40 | 26/jan/10 | 1 | *Monodelphis* | *glirina* | MOGL | 482 | M | 1 |
| CANGA 1 | B | 32 | 26/jan/10 | 1 | *Monodelphis* | *glirina* | MOGL | 498 | M | 0 |
| CANGA 1 | B | 28 | 26/jan/10 | 1 | *Monodelphis* | *glirina* | MOGL | 25 | F | 1 |
| CANGA 1 | C | 36 | 26/jan/10 | 1 | *Monodelphis* | *glirina* | MOGL | 444 | M | 1 |
| CANGA 1 | C | 12 | 27/jan/10 | 1 | *Monodelphis* | *glirina* | MOGL | 526 | F | 0 |
| CANGA 1 | C | 18 | 27/jan/10 | 1 | *Monodelphis* | *glirina* | MOGL | 528 | M | 0 |
| CANGA 1 | A | 12 | 27/jan/10 | 1 | *Monodelphis* | *glirina* | MOGL | 530 | M | 0 |
| CANGA 1 | A | 26 | 27/jan/10 | 1 | *Monodelphis* | *glirina* | MOGL | 534 | F | 0 |
| CANGA 1 | A | 40 | 27/jan/10 | 1 | *Monodelphis* | *glirina* | MOGL | 536 | F | 0 |
| CANGA 1 | B | 8 | 27/jan/10 | 1 | *Monodelphis* | *glirina* | MOGL | 538 | M | 0 |
| CANGA 1 | B | 12 | 27/jan/10 | 1 | *Monodelphis* | *glirina* | MOGL | 540 | M | 0 |
| CANGA 1 | B | 24 | 27/jan/10 | 1 | *Monodelphis* | *glirina* | MOGL | 544 | F | 0 |
| CANGA 1 | B | 18 | 27/jan/10 | 1 | *Monodelphis* | *glirina* | MOGL | 546 | F | 0 |
| CANGA 1 | B | 4 | 27/jan/10 | 1 | *Monodelphis* | *glirina* | MOGL | 550 | F | 0 |
| CANGA 1 | B | 32 | 27/jan/10 | 1 | *Monodelphis* | *glirina* | MOGL | 548 | F | 0 |
| CANGA 1 | B | 30 | 28/jan/10 | 1 | *Monodelphis* | *glirina* | MOGL | 562 | M | 0 |
| CANGA 1 | B | 18 | 28/jan/10 | 1 | *Monodelphis* | *glirina* | MOGL | 552 | M | 0 |
| CANGA 1 | B | 12 | 28/jan/10 | 1 | *Monodelphis* | *glirina* | MOGL | 554 | M | 0 |
| CANGA 1 | A | 14 | 28/jan/10 | 1 | *Monodelphis* | *glirina* | MOGL | 556 | F | 0 |
| CANGA 1 | A | 28 | 28/jan/10 | 1 | *Monodelphis* | *glirina* | MOGL | 558 | M | 0 |
| CANGA 1 | D | 13 | 28/jan/10 | 1 | *Monodelphis* | *glirina* | MOGL | 560 | M | 0 |
| CANGA 1 | D | 10 | 28/jan/10 | 1 | *Monodelphis* | *glirina* | MOGL | 568 | M | 0 |
| CANGA 1 | D | 26 | 28/jan/10 | 1 | *Monodelphis* | *glirina* | MOGL | 564 | F | 0 |
| FLORESTA 2 | G | 19 | 30/jan/10 | 1 | *Monodelphis* | *glirina* | MOGL | 570 | M | 0 |
| FLORESTA 2 | A | 6 | 30/jan/10 | 1 | *Monodelphis* | *glirina* | MOGL | . |  | 0 |
| FLORESTA 2 | D | 31 | 31/jan/10 | 1 | *Monodelphis* | *glirina* | MOGL | 566 | F | 0 |
| FLORESTA 2 | E | 15 | 02/feb/2010 | 1 | *Monodelphis* | *glirina* | MOGL | . | M | 0 |
| FLORESTA 1 | E | 25 | 08/feb/2010 | 1 | *Monodelphis* | *glirina* | MOGL | . |  | 0 |
| FLORESTA 1 | G | 1 | 27/feb/2010 | 1 | *Monodelphis* | *glirina* | MOGL | 594 | M | 0 |
| FLORESTA 1 | E | 15 | 28/feb/2010 | 1 | *Monodelphis* | *glirina* | MOGL | . |  | 0 |
| FLORESTA 1 | E | 13 | 1-mar-10 | 1 | *Monodelphis* | *glirina* | MOGL | 628 | M | 0 |
| FLORESTA 1 | E | 5 | 1-mar-10 | 1 | *Monodelphis* | *glirina* | MOGL | 632 | M | 0 |
| FLORESTA 1 | E | 12 | 1-mar-10 | 1 | *Monodelphis* | *glirina* | MOGL | 634 | F | 0 |
| FLORESTA 1 | E | 12 | 1-mar-10 | 1 | *Monodelphis* | *glirina* | MOGL | 636 | M | 0 |
| FLORESTA 1 | B | 14 | 3-mar-10 | 1 | *Monodelphis* | *glirina* | MOGL | 644 | F | 0 |
| FLORESTA 2 | G | 3 | 6-mar-10 | 1 | *Monodelphis* | *glirina* | MOGL | 654 | M | 0 |
| FLORESTA 2 | C | 10 | 6-mar-10 | 1 | *Monodelphis* | *glirina* | MOGL | 656 | F | 0 |
| FLORESTA 2 | C | 3 | 7-mar-10 | 1 | *Monodelphis* | *glirina* | MOGL | 664 | M | 0 |
| FLORESTA 2 | C | 10 | 7-mar-10 | 1 | *Monodelphis* | *glirina* | MOGL | 668 | M | 0 |
| FLORESTA 2 | G | 14 | 7-mar-10 | 1 | *Monodelphis* | *glirina* | MOGL | 670 | F | 0 |
| FLORESTA 2 | B | 1 | 7-mar-10 | 1 | *Monodelphis* | *glirina* | MOGL | . |  | 0 |
| FLORESTA 2 | B | 2 | 8-mar-10 | 1 | *Monodelphis* | *glirina* | MOGL | 672 | M | 0 |
| FLORESTA 2 | A | 2 | 8-mar-10 | 1 | *Monodelphis* | *glirina* | MOGL | 674 | F | 0 |
| FLORESTA 2 | A | 14 | 8-mar-10 | 1 | *Monodelphis* | *glirina* | MOGL | 676 | M | 0 |
| FLORESTA 2 | G | 6 | 8-mar-10 | 1 | *Monodelphis* | *glirina* | MOGL | 654 | M | 1 |
| FLORESTA 2 | D | 5 | 9-mar-10 | 1 | *Monodelphis* | *glirina* | MOGL | 690 | M | 0 |
| FLORESTA 2 | G | 13 | 9-mar-10 | 1 | *Monodelphis* | *glirina* | MOGL | 692 | M | 0 |
| FLORESTA 2 | C | 8 | 9-mar-10 | 1 | *Monodelphis* | *glirina* | MOGL | 694 | F | 0 |
| FLORESTA 2 | C | 9 | 9-mar-10 | 1 | *Monodelphis* | *glirina* | MOGL | 698 | M | 0 |
| FLORESTA 2 | C | 14 | 10-mar-10 | 1 | *Monodelphis* | *glirina* | MOGL | 664 | . | 0 |
| FLORESTA 2 | G | 3 | 10-mar-10 | 1 | *Monodelphis* | *glirina* | MOGL | 654 | M | 0 |
| FLORESTA 2 | B | 3 | 10-mar-10 | 1 | *Monodelphis* | *glirina* | MOGL | 708 | M | 0 |
| FLORESTA 2 | D | 2 | 11-mar-10 | 1 | *Monodelphis* | *glirina* | MOGL | 710 | M | 0 |
| FLORESTA 2 | A | 2 | 13-mar-10 | 1 | *Monodelphis* | *glirina* | MOGL | 732 | M | 0 |
| FLORESTA 2 | E | 14 | 14-mar-10 | 1 | *Monodelphis* | *glirina* | MOGL | 738 | M | 0 |
| FLORESTA 2 | E | 6 | 14-mar-10 | 1 | *Monodelphis* | *glirina* | MOGL | 742 | M | 0 |
| FLORESTA 1 | G | 8 | 25-fev-10 | 1 | *Monodelphis* | *aff. kunsi* | MOKU | . | M | 0 |
| FLORESTA 2 | C | 40 | 01/feb/2010 | 1 | *Monodelphis* | *sp. D* | MOsp. | . |  | 0 |
| FLORESTA 2 | B | 34 | 04/feb/2010 | 1 | *Monodelphis* | *sp. D* | MOsp. | . | M | 0 |
| FLORESTA 1 | E | 16 | 09/feb/2010 | 1 | *Monodelphis* | *sp. D* | MOsp. | . | M | 0 |
| FLORESTA 1 | E | 31 | 09/feb/2010 | 1 | *Monodelphis* | *sp. D* | MOsp. | . | F | 0 |
| FLORESTA 1 | C | 49 | 10/feb/2010 | 1 | *Monodelphis* | *sp. D* | MOsp. | 586 | F | 0 |
| FLORESTA 1 | E | 12 | 25/feb/2010 | 1 | *Monodelphis* | *sp. D* | MOsp. | . |  | . |
| FLORESTA 1 | B | 1 | 27/feb/2010 | 1 | *Monodelphis* | *sp. D* | MOsp. | 598 | M | 0 |
| FLORESTA 1 | E | 4 | 27/feb/2010 | 1 | *Monodelphis* | *sp. D* | MOsp. | . | M | 0 |
| FLORESTA 1 | E | 11 | 27/feb/2010 | 1 | *Monodelphis* | *sp. D* | MOsp. | 600 | M | 0 |
| FLORESTA 1 | A | 6 | 28/feb/2011 | 1 | *Monodelphis* | *sp. D* | MOsp. | 638 | M | 0 |
| FLORESTA 1 | E | 12 | 2-mar-10 | 1 | *Monodelphis* | *sp. D* | MOsp. | 642 | M | 0 |
| FLORESTA 2 | E | 15 | 6-mar-10 | 1 | *Monodelphis* | *sp. D* | MOsp. | 658 | . | 0 |
| FLORESTA 2 | A | 1 | 7-mar-10 | 1 | *Monodelphis* | *sp. D* | MOsp. | 662 | M | 0 |
| FLORESTA 2 | D | 4 | 7-mar-10 | 1 | *Monodelphis* | *sp. D* | MOsp. | 666 | M | 0 |
| FLORESTA 2 | A | 14 | 10-mar-10 | 1 | *Monodelphis* | *sp. D* | MOsp. | 706 | M | 0 |
| FLORESTA 2 | D | 5 | 12-mar-10 | 1 | *Monodelphis* | *sp. D* | MOsp. | 730 | M | 0 |
| FLORESTA 2 | C | 13 | 14-mar-10 | 1 | *Monodelphis* | *sp. D* | MOsp. | 662 | M | 1 |
| FLORESTA 2 | E | 14 | 14-mar-10 | 1 | *Monodelphis* | *sp. D* | MOsp. | 666 | M | 1 |
| FLORESTA 2 | C | 6 | 14-mar-10 | 1 | *Monodelphis* | *sp. D* | MOsp. | 664 | M | 1 |
| FLORESTA 1 | D | 12 | 26-fev-10 | 1 | *Neacomys* | *cf. paracou* | NEPA | . | F | 0 |
| CANGA 2 | C | 5 | 16-jan-10 | 1 | *Necromys* | *lasiurus* | NELA | . | F | 0 |
| CANGA 2 | E | 13 | 16-jan-10 | 1 | *Necromys* | *lasiurus* | NELA | 354 | M | 0 |
| CANGA 2 | G | 41 | 16-jan-10 | 1 | *Necromys* | *lasiurus* | NELA | . | M | 0 |
| CANGA 2 | A | 3 | 18-jan-10 | 1 | *Necromys* | *lasiurus* | NELA | 363 | M | 0 |
| CANGA 2 | B | 5 | 18-jan-10 | 1 | *Necromys* | *lasiurus* | NELA | . | F | 0 |
| CANGA 2 | B | 28 | 18-jan-10 | 1 | *Necromys* | *lasiurus* | NELA | 365 | F | 0 |
| CANGA 2 | B | 6 | 18-jan-10 | 1 | *Necromys* | *lasiurus* | NELA | 372 | M | 0 |
| CANGA 2 | C | 48 | 18-jan-10 | 1 | *Necromys* | *lasiurus* | NELA | . | F | 0 |
| CANGA 2 | C | 19 | 18-jan-10 | 1 | *Necromys* | *lasiurus* | NELA | . |  | 0 |
| CANGA 2 | C | 11 | 19-jan-10 | 1 | *Necromys* | *lasiurus* | NELA | 404 | F | 0 |
| CANGA 2 | D | 30 | 18-jan-10 | 1 | *Necromys* | *lasiurus* | NELA | 370 | M | 0 |
| CANGA 2 | C | 22 | 20-jan-10 | 1 | *Necromys* | *lasiurus* | NELA | 410 | M | 0 |
| CANGA 2 | D | 15 | 20-jan-10 | 1 | *Necromys* | *lasiurus* | NELA | . | M | 0 |
| CANGA 2 | E | 17 | 20-jan-10 | 1 | *Necromys* | *lasiurus* | NELA | . | M | 0 |
| CANGA 2 | A | 31 | 21-jan-10 | 1 | *Necromys* | *lasiurus* | NELA | 498 | M | 0 |
| CANGA 2 | B | 18 | 21-jan-10 | 1 | *Necromys* | *lasiurus* | NELA | 420 | M | 0 |
| CANGA 2 | D | 30 | 21-jan-10 | 1 | *Necromys* | *lasiurus* | NELA | 433 | F | 0 |
| CANGA 2 | D | 46 | 21-jan-10 | 1 | *Necromys* | *lasiurus* | NELA | 434 | M | 0 |
| CANGA 2 | D | 48 | 21-jan-10 | 1 | *Necromys* | *lasiurus* | NELA | 431 | F | 0 |
| CANGA 1 | A | 13 | 23-jan-10 | 1 | *Necromys* | *lasiurus* | NELA | 450 | F | 0 |
| CANGA 2 | C | 36 | 20-jan-10 | 1 | *Nectomys* | *rattus* | NERA | . | F | 0 |
| CANGA 2 | D | 7 | 20-jan-10 | 1 | *Nectomys* | *rattus* | NERA | . | M | 0 |
| FLORESTA 2 | A | 6 | 11-mar-10 | 1 | *Neusticomys* | *ferreirai* | NEFE | . | F | . |
| FLORESTA 1 | A | 41 | 09/feb/2010 | 1 | *Oecomys* | sp. | OECO | . | M | 0 |
| FLORESTA 1 | E | 9 | 09/feb/2010 | 1 | *Oecomys* | sp. | OECO | . | F | 0 |
| FLORESTA 1 | C | 12 | 25/feb/2010 | 1 | *Oecomys* | sp. | OECO | 0 | F | 0 |
| FLORESTA 1 | D | 10 | 26/feb/2010 | 1 | *Oecomys* | sp. | OECO | . |  | . |
| FLORESTA 1 | D | 9 | 28/feb/2010 | 1 | *Oecomys* | sp. | OECO | . | F | 0 |
| FLORESTA 1 | C | 1 | 28/feb/2010 | 1 | *Oecomys* | sp. | OECO | . |  | 0 |
| FLORESTA 1 | D | 13 | 28/feb/2010 | 1 | *Oecomys* | sp. | OECO | 602 |  | 0 |
| FLORESTA 1 | C | 10 | 1-mar-10 | 1 | *Oecomys* | sp. | OECO | 618 | M | 0 |
| FLORESTA 1 | C | 2 | 1-mar-10 | 1 | *Oecomys* | sp. | OECO | 622 | F | 0 |
| FLORESTA 1 | B | 6 | 3-mar-10 | 1 | *Oecomys* | sp. | OECO | 618 | M | 1 |
| FLORESTA 1 | B | 2 | 2-mar-10 | 1 | *Oecomys* | sp. | OECO | . | F | . |
| FLORESTA 2 | C | 12 | 14-mar-10 | 1 | *Oecomys* | sp. | OECO | . | M | 0 |
| FLORESTA 2 | C | 5 | 14-mar-10 | 1 | *Oecomys* | sp. | OECO | . |  | 0 |
| FLORESTA 2 | E | 15 | 14-mar-10 | 1 | *Oecomys* | sp. | OECO | 740 | M | 0 |
| FLORESTA 2 | A | 4 | 14-mar-10 | 1 | *Oecomys* | sp. | OECO | 746 | F | 0 |
| FLORESTA 2 | B | 6 | 14-mar-10 | 1 | *Oecomys* | sp. | OECO | 748 | F | 0 |
| FLORESTA 2 | B | 7 | 13-mar-10 | 1 | *Oligoryzomys* | *microtis* | OLMI | . | F | 0 |
| FLORESTA 2 | A | 10 | 14-mar-10 | 1 | *Oligoryzomys* | *microtis* | OLMI | . | F | 0 |
| CANGA 2 | B | 32 | 16-jan-10 | 1 | *Oxymycterus* | *amazonicus* | OXAM | 344 | F | 0 |
| CANGA 2 | D | 3 | 17-jan-10 | 1 | *Oxymycterus* | *amazonicus* | OXAM | . |  | 0 |
| CANGA 2 | B | 32 | 18-jan-10 | 1 | *Oxymycterus* | *amazonicus* | OXAM | . | M | 0 |
| CANGA 2 | C | 30 | 19-jan-10 | 1 | *Oxymycterus* | *amazonicus* | OXAM | 344 | M | 1 |
| CANGA 2 | E | 40 | 19-jan-10 | 1 | *Oxymycterus* | *amazonicus* | OXAM | . | F | 0 |
| CANGA 2 | D | 3 | 20-jan-10 | 1 | *Oxymycterus* | *amazonicus* | OXAM | . |  | 0 |
| CANGA 2 | C | 36 | 21-jan-10 | 1 | *Oxymycterus* | *amazonicus* | OXAM | 245 | M | 1 |
| CANGA 1 | A | 43 | 23-jan-10 | 1 | *Oxymycterus* | *amazonicus* | OXAM | 80 | M | 1 |
| CANGA 1 | B | 2 | 25-jan-10 | 1 | *Oxymycterus* | *amazonicus* | OXAM | . | F | 0 |
| FLORESTA 1 | E | 8 | 1-mar-10 | 1 | *Oxymycterus* | *amazonicus* | OXAM | 610 | M | 0 |
| FLORESTA 2 | C | 6 | 11-mar-10 | 1 | *Oxymycterus* | *amazonicus* | OXAM | 714 | M | 0 |
| FLORESTA 2 | B | 3 | 11-mar-10 | 1 | *Oxymycterus* | *amazonicus* | OXAM | 722 | M | 0 |
| FLORESTA 2 | G | 13 | 11-mar-10 | 1 | *Oxymycterus* | *amazonicus* | OXAM | 728 | F | 0 |
| FLORESTA 2 | B | 15 | 14-mar-10 | 1 | *Oxymycterus* | *amazonicus* | OXAM | 714 | M | 1 |
| CANGA 2 | B | 55 | 16-jan-10 | 1 | *Proechimys* | *roberti* | PRRO | 351 | M | 1 |
| CANGA 2 | E | 25 | 16-jan-10 | 1 | *Proechimys* | *roberti* | PRRO | 373 | F | 1 |
| CANGA 1 | A | 8 | 24-jan-10 | 1 | *Proechimys* | *roberti* | PRRO | . |  | 0 |
| CANGA 1 | D | 33 | 26-jan-10 | 1 | *Proechimys* | *roberti* | PRRO | . | F | 0 |
| CANGA 1 | A | 6 | 27-jan-10 | 1 | *Proechimys* | *roberti* | PRRO | 40 | F | 1 |
| FLORESTA 1 | G | 8 | 08/feb/2010 | 1 | *Proechimys* | *roberti* | PRRO | . | F | 0 |
| FLORESTA 1 | D | 5 | 25-fev-10 | 1 | *Proechimys* | *roberti* | PRRO | 588 | F | 0 |
| FLORESTA 1 | B | 14 | 26-fev-10 | 1 | *Proechimys* | *roberti* | PRRO | 590 | F | 0 |
| FLORESTA 1 | D | 6 | 27-fev-10 | 1 | *Proechimys* | *roberti* | PRRO | 596 | M | 0 |
| FLORESTA 1 | C | 9 | 1-mar-10 | 1 | *Proechimys* | *roberti* | PRRO | 624 | M | 0 |
| FLORESTA 2 | B | 2 | 10-mar-10 | 1 | *Proechimys* | *roberti* | PRRO | 704 | F | 0 |
| CANGA 2 | C | 41 | 18-jan-10 | 1 | *Rhipidomys* | *emiliae* | RHEM | . | F | 0 |
| CANGA 2 | C | 27 | 20-jan-10 | 1 | *Rhipidomys* | *emiliae* | RHEM | . | F | 0 |
| CANGA 1 | E | 2 | 23-jan-10 | 1 | *Rhipidomys* | *emiliae* | RHEM | . |  | 0 |
| FLORESTA 2 | B | 35 | 31-jan-10 | 1 | *Rhipidomys* | *emiliae* | RHEM | . | F | 0 |
| FLORESTA 2 | D | 44 | 02/feb/2010 | 1 | *Rhipidomys* | *emiliae* | RHEM | . | F | 0 |
| FLORESTA 2 | G | 11 | 14-mar-10 | 1 | *Rhipidomys* | *emiliae* | RHEM | 736 | M | 0 |
| FLORESTA 2 | C | 50 | 25-ago-10 | 2 | *Didelphis* | *marsupialis* | DIMA | 4802.48 | F | 0 |
| FLORESTA 2 | C | 15 | 29-ago-10 | 2 | *Didelphis* | *marsupialis* | DIMA | 4804.481 | M | 0 |
| FLORESTA 2 | G | 8 | 13-ago-10 | 2 | *Euryoryzomys* | *emmonsae* | EUEM | . | F | 0 |
| FLORESTA 1 | A | 49 | 20-ago-10 | 2 | *Euryoryzomys* | *emmonsae* | EUEM | . |  | 0 |
| FLORESTA 2 | A | 27 | 26-ago-10 | 2 | *Glironia* | *venusta* | GLVE | . | M | 0 |
| FLORESTA 1 | G | 17 | 19-ago-10 | 2 | *Marmosa* | *murina* | MAMU | . | F | 0 |
| FLORESTA 1 | G | 12 | 20-ago-10 | 2 | *Marmosa* | *murina* | MAMU | . | F | 0 |
| FLORESTA 2 | D | 11 | 25-ago-10 | 2 | *Marmosa* | *murina* | MAMU | 610 | M | 0 |
| FLORESTA 2 | A | 49 | 26-ago-10 | 2 | *Marmosa* | *murina* | MAMU | 612 | M | 0 |
| FLORESTA 2 | G | 26 | 27-ago-10 | 2 | *Marmosa* | *murina* | MAMU | 616 | F | 0 |
| FLORESTA 2 | D | 11 | 28-ago-10 | 2 | *Marmosa* | *murina* | MAMU | . | F | 0 |
| FLORESTA 2 | E | 10 | 28-ago-10 | 2 | *Marmosa* | *murina* | MAMU | 620 | F | 0 |
| CANGA 2 | N INF | . | 31-ago-10 | 2 | *Marmosa* | *murina* | MAMU | 667 | F | 0 |
| CANGA 2 | A | 53 | 01/sep/2010 | 2 | *Marmosa* | *murina* | MAMU | 702 | F | 0 |
| CANGA 2 | D | 27 | 01/sep-2010 | 2 | *Marmosa* | *murina* | MAMU | 722 | M | 0 |
| CANGA 2 | C | 29 | 02/sep-2010 | 2 | *Marmosa* | *murina* | MAMU | 728 | M | 0 |
| CANGA 2 | A | 18 | 02/sep-2010 | 2 | *Marmosa* | *murina* | MAMU | 426 | F | 1 |
| CANGA 2 | E | 11 | 02/sep-2010 | 2 | *Marmosa* | *murina* | MAMU | 355 | F | 1 |
| CANGA 2 | A | 2 | 03/sep-2010 | 2 | *Marmosa* | *murina* | MAMU | 780 | F | 0 |
| CANGA 2 | G | 20 | 03/sep-2010 | 2 | *Marmosa* | *murina* | MAMU | 782 | M | 0 |
| CANGA 2 | B | 49 | 04/sep-2010 | 2 | *Marmosa* | *murina* | MAMU | 786 | F | 0 |
| CANGA 2 | D | 38 | 04/sep-2010 | 2 | *Marmosa* | *murina* | MAMU | 790 | F | 0 |
| CANGA 2 | G | 10 | 04/sep-2010 | 2 | *Marmosa* | *murina* | MAMU | 796 | M | 0 |
| CANGA 2 | G | 8 | 05/sep-2010 | 2 | *Marmosa* | *murina* | MAMU | 816 | F | 0 |
| CANGA 1 | B | 33 | 07/sep-2010 | 2 | *Marmosa* | *murina* | MAMU | 830 | M | 0 |
| CANGA 1 | E | 1 | 08/sep-2010 | 2 | *Marmosa* | *murina* | MAMU | 862 | M | 0 |
| CANGA 1 | A | 20 | 10/sep-2010 | 2 | *Marmosa* | *murina* | MAMU | 898 | M | 0 |
| CANGA 1 | D | 40 | 10/sep-2010 | 2 | *Marmosa* | *murina* | MAMU | 900 | F | 0 |
| CANGA 1 | C | 9 | 12/sep-2010 | 2 | *Marmosa* | *murina* | MAMU | 950 | M | 0 |
| CANGA 1 | D | 3 | 12/sep-2010 | 2 | *Marmosa* | *murina* | MAMU | 956 | M | 0 |
| FLORESTA 2 | D | 6 | 6-ago-10 | 2 | *Marmosops* | *pinheiroi* | MAPI | . | M | 0 |
| FLORESTA 2 | C | 12 | 6-ago-10 | 2 | *Marmosops* | *pinheiroi* | MAPI | . | M | 0 |
| FLORESTA 1 | G | 4 | 7-ago-10 | 2 | *Marmosops* | *pinheiroi* | MAPI | 470 | M | 0 |
| FLORESTA 2 | D | 1 | 7-ago-10 | 2 | *Marmosops* | *pinheiroi* | MAPI | 474 | M | 0 |
| FLORESTA 2 | G | 12 | 7-ago-10 | 2 | *Marmosops* | *pinheiroi* | MAPI | . | M | 0 |
| FLORESTA 2 | D | 8 | 8-ago-10 | 2 | *Marmosops* | *pinheiroi* | MAPI | 476 | M | 0 |
| FLORESTA 1 | C | 5 | 10-ago-10 | 2 | *Marmosops* | *pinheiroi* | MAPI | 482 | F | 0 |
| FLORESTA 2 | G | 1 | 11-ago-10 | 2 | *Marmosops* | *pinheiroi* | MAPI | . | F | 0 |
| FLORESTA 2 | D | 4 | 12-ago-10 | 2 | *Marmosops* | *pinheiroi* | MAPI | 490 | F | 0 |
| FLORESTA 2 | G | 6 | 14-ago-10 | 2 | *Marmosops* | *pinheiroi* | MAPI | 494 | M | 0 |
| FLORESTA 1 | C | 19 | 21-ago-10 | 2 | *Marmosa* | *demerarae* | MADE | . | F | 0 |
| FLORESTA 2 | A | 27 | 27-ago-10 | 2 | *Marmosa* | *demerarae* | MADE | . | M | 0 |
| FLORESTA 2 | G | 12 | 29-ago-10 | 2 | *Marmosa* | *demerarae* | MADE | 624 | M | 0 |
| FLORESTA 2 | G | 6 | 29-ago-10 | 2 | *Marmosa* | *demerarae* | MADE | 622 | F | 0 |
| CANGA 2 | E | 12 | 31-ago-10 | 2 | *Marmosa* | *demerarae* | MADE | 355 | F | 1 |
| CANGA 2 | A | 40 | 3-set-10 | 2 | *Marmosa* | *demerarae* | MADE | 764 | F | 0 |
| CANGA 1 | A | 4 | 8-set-10 | 2 | *Marmosa* | *demerarae* | MADE | 868 | M | 0 |
| CANGA 2 | E | 37 | 31-ago-10 | 2 | *Monodelphis* | *glirina* | MOGL | 658 | M | 0 |
| CANGA 2 | C | 52 | 31-ago-10 | 2 | *Monodelphis* | *glirina* | MOGL | 664 | F | 0 |
| CANGA 2 | B | 39 | 31-ago-10 | 2 | *Monodelphis* | *glirina* | MOGL | 660 | M | 0 |
| CANGA 2 | C | 31 | 31-ago-10 | 2 | *Monodelphis* | *glirina* | MOGL | 666 | M | 0 |
| CANGA 2 | G | 48 | 31-ago-10 | 2 | *Monodelphis* | *glirina* | MOGL | 670 | F | 0 |
| CANGA 2 | G | 44 | 31-ago-10 | 2 | *Monodelphis* | *glirina* | MOGL | 212 | F | 1 |
| CANGA 2 | C | 54 | 31-ago-10 | 2 | *Monodelphis* | *glirina* | MOGL | 672 | M | 0 |
| CANGA 2 | E | 42 | 31-ago-10 | 2 | *Monodelphis* | *glirina* | MOGL | . | F | 0 |
| CANGA 2 | C | 42 | 31-ago-10 | 2 | *Monodelphis* | *glirina* | MOGL | 671 | M | 0 |
| CANGA 2 | G | 27 | 31-ago-10 | 2 | *Monodelphis* | *glirina* | MOGL | 674 | M | 0 |
| CANGA 2 | N INF | . | 1-set-10 | 2 | *Monodelphis* | *glirina* | MOGL | 673 | F | 0 |
| CANGA 2 | E | 10 | 31-ago-10 | 2 | *Monodelphis* | *glirina* | MOGL | 675 | F | 1 |
| CANGA 2 | G | 58 | 31-ago-10 | 2 | *Monodelphis* | *glirina* | MOGL | 359 | M | 1 |
| CANGA 2 | A | 16 | 1-set-10 | 2 | *Monodelphis* | *glirina* | MOGL | 375 | M | 1 |
| CANGA 2 | B | 10 | 1-set-10 | 2 | *Monodelphis* | *glirina* | MOGL | 704 | F | 0 |
| CANGA 2 | E | 13 | 1-set-10 | 2 | *Monodelphis* | *glirina* | MOGL | 706 | F | 0 |
| CANGA 2 | E | 15 | 1-set-10 | 2 | *Monodelphis* | *glirina* | MOGL | 708 | M | 0 |
| CANGA 2 | A | 29 | 1-set-10 | 2 | *Monodelphis* | *glirina* | MOGL | 413 | VER DADOS | 1 |
| CANGA 2 | D | 14 | 1-set-10 | 2 | *Monodelphis* | *glirina* | MOGL | 718 | M | 0 |
| CANGA 2 | D | 35 | 1-set-10 | 2 | *Monodelphis* | *glirina* | MOGL | 720 | M | 0 |
| CANGA 2 | D | 10 | 1-set-10 | 2 | *Monodelphis* | *glirina* | MOGL | 724 | M | 0 |
| CANGA 2 | D | 8 | 1-set-10 | 2 | *Monodelphis* | *glirina* | MOGL | 726 | F | 0 |
| CANGA 2 | C | 30 | 1-set-10 | 2 | *Monodelphis* | *glirina* | MOGL | 730 | F | 0 |
| CANGA 2 | G | 42 | 2-set-10 | 2 | *Monodelphis* | *glirina* | MOGL | 732 | F | 0 |
| CANGA 2 | E | 9 | 2-set-10 | 2 | *Monodelphis* | *glirina* | MOGL | 736 | F | 0 |
| CANGA 2 | D | 14 | 2-set-10 | 2 | *Monodelphis* | *glirina* | MOGL | 396 | M | 1 |
| CANGA 2 | E | 39 | 2-set-10 | 2 | *Monodelphis* | *glirina* | MOGL | 738 | F | 0 |
| CANGA 2 | C | 19 | 2-set-10 | 2 | *Monodelphis* | *glirina* | MOGL | 740 | M | 0 |
| CANGA 2 | D | 10 | 2-set-10 | 2 | *Monodelphis* | *glirina* | MOGL | 744 | M | 0 |
| CANGA 2 | G | 22 | 2-set-10 | 2 | *Monodelphis* | *glirina* | MOGL | 748 | F | 0 |
| CANGA 2 | A | 51 | 2-set-10 | 2 | *Monodelphis* | *glirina* | MOGL | 754 | M | 0 |
| CANGA 2 | D | 34 | 2-set-10 | 2 | *Monodelphis* | *glirina* | MOGL | 405 | F | 1 |
| CANGA 2 | G | 46 | 2-set-10 | 2 | *Monodelphis* | *glirina* | MOGL | 760 | M | 0 |
| CANGA 2 | D | 12 | 3-set-10 | 2 | *Monodelphis* | *glirina* | MOGL | 774 | F | 0 |
| CANGA 2 | G | 39 | 3-set-10 | 2 | *Monodelphis* | *glirina* | MOGL | 776 | M | 0 |
| CANGA 2 | D | 49 | 4-set-10 | 2 | *Monodelphis* | *glirina* | MOGL | 784 | M | 0 |
| CANGA 2 | E | 15 | 4-set-10 | 2 | *Monodelphis* | *glirina* | MOGL | 788 | M | . |
| CANGA 2 | G | 20 | 4-set-10 | 2 | *Monodelphis* | *glirina* | MOGL | 792 | M | 0 |
| CANGA 2 | G | 18 | 4-set-10 | 2 | *Monodelphis* | *glirina* | MOGL | 794 | M | 0 |
| CANGA 2 | C | 25 | 5-set-10 | 2 | *Monodelphis* | *glirina* | MOGL | 810 | M | 0 |
| CANGA 2 | G | 35 | 5-set-10 | 2 | *Monodelphis* | *glirina* | MOGL | 802 | M | 0 |
| CANGA 1 | A | 9 | 7-set-10 | 2 | *Monodelphis* | *glirina* | MOGL | 554 | M | 1 |
| CANGA 1 | D | 16 | 7-set-10 | 2 | *Monodelphis* | *glirina* | MOGL | 439 | M | 1 |
| CANGA 1 | D | 9 | 7-set-10 | 2 | *Monodelphis* | *glirina* | MOGL | 818 | F | 0 |
| CANGA 1 | E | 22 | 7-set-10 | 2 | *Monodelphis* | *glirina* | MOGL | 820 | F | 0 |
| CANGA 1 | B | 12 | 7-set-10 | 2 | *Monodelphis* | *glirina* | MOGL | 822 | M | 0 |
| CANGA 1 | E | 19 | 7-set-10 | 2 | *Monodelphis* | *glirina* | MOGL | 824 | F | 0 |
| CANGA 1 | B | 26 | 7-set-10 | 2 | *Monodelphis* | *glirina* | MOGL | 826 | M | 0 |
| CANGA 1 | D | 1 | 7-set-10 | 2 | *Monodelphis* | *glirina* | MOGL | 828 | M | 0 |
| CANGA 1 | B | 5 | 7-set-10 | 2 | *Monodelphis* | *glirina* | MOGL | 832 | F | 0 |
| CANGA 1 | C | 21 | 7-set-10 | 2 | *Monodelphis* | *glirina* | MOGL | 842 | F | 0 |
| CANGA 1 | C | 18 | 7-set-10 | 2 | *Monodelphis* | *glirina* | MOGL | 844 | M | 0 |
| CANGA 1 | C | 17 | 7-set-10 | 2 | *Monodelphis* | *glirina* | MOGL | 846 | F | 0 |
| CANGA 1 | C | 16 | 7-set-10 | 2 | *Monodelphis* | *glirina* | MOGL | 848 | M | 0 |
| CANGA 1 | C | 12 | 7-set-10 | 2 | *Monodelphis* | *glirina* | MOGL | 850 | F | 0 |
| CANGA 1 | C | 27 | 7-set-10 | 2 | *Monodelphis* | *glirina* | MOGL | . | M | 0 |
| CANGA 1 | B | 21 | 7-set-10 | 2 | *Monodelphis* | *glirina* | MOGL | . | F | 0 |
| CANGA 1 | C | 14 | 7-set-10 | 2 | *Monodelphis* | *glirina* | MOGL | 490 |  | 1 |
| CANGA 1 | D | 24 | 8-set-10 | 2 | *Monodelphis* | *glirina* | MOGL | 852 | M | 0 |
| CANGA 1 | B | 7 | 8-set-10 | 2 | *Monodelphis* | *glirina* | MOGL | 858 | F | 0 |
| CANGA 1 | E | 26 | 8-set-10 | 2 | *Monodelphis* | *glirina* | MOGL | 866 | M | 0 |
| CANGA 1 | D | 5 | 9-set-10 | 2 | *Monodelphis* | *glirina* | MOGL | 874 | M | 0 |
| CANGA 1 | D | 2 | 9-set-10 | 2 | *Monodelphis* | *glirina* | MOGL | 880 | M | 0 |
| CANGA 1 | C | 22 | 9-set-10 | 2 | *Monodelphis* | *glirina* | MOGL | 882 | F | 0 |
| CANGA 1 | A | 10 | 9-set-10 | 2 | *Monodelphis* | *glirina* | MOGL | 554 | M | 1 |
| CANGA 1 | B | 9 | 9-set-10 | 2 | *Monodelphis* | *glirina* | MOGL | 886 | M | 0 |
| CANGA 1 | C | 12 | 9-set-10 | 2 | *Monodelphis* | *glirina* | MOGL | 888 | F | 0 |
| CANGA 1 | E | 23 | 9-set-10 | 2 | *Monodelphis* | *glirina* | MOGL | 890 | F | 0 |
| CANGA 1 | C | 3 | 9-set-10 | 2 | *Monodelphis* | *glirina* | MOGL | 894 | F | 0 |
| CANGA 1 | C | 24 | 9-set-10 | 2 | *Monodelphis* | *glirina* | MOGL | 896 | M | 0 |
| CANGA 1 | C | 29 | 9-set-10 | 2 | *Monodelphis* | *glirina* | MOGL | . | M | 0 |
| CANGA 1 | C | 8 | 9-set-10 | 2 | *Monodelphis* | *glirina* | MOGL | . | F | 0 |
| CANGA 1 | A | 14 | 10-set-10 | 2 | *Monodelphis* | *glirina* | MOGL | 902 | F | 0 |
| CANGA 1 | A | 19 | 10-set-10 | 2 | *Monodelphis* | *glirina* | MOGL | 904 | F | 0 |
| CANGA 1 | B | 22 | 10-set-10 | 2 | *Monodelphis* | *glirina* | MOGL | 906 | F | 0 |
| CANGA 1 | B | 15 | 11-set-10 | 2 | *Monodelphis* | *glirina* | MOGL | 914 | M | 0 |
| CANGA 1 | E | 23 | 11-set-10 | 2 | *Monodelphis* | *glirina* | MOGL | 916 | F | 0 |
| CANGA 1 | E | 5 | 11-set-10 | 2 | *Monodelphis* | *glirina* | MOGL | 918 | M | 0 |
| CANGA 1 | D | 3 | 11-set-10 | 2 | *Monodelphis* | *glirina* | MOGL | 920 | M | 0 |
| CANGA 1 | A | 20 | 11-set-10 | 2 | *Monodelphis* | *glirina* | MOGL | 922 | F | 0 |
| CANGA 1 | C | 24 | 11-set-10 | 2 | *Monodelphis* | *glirina* | MOGL | 926 | F | 0 |
| CANGA 1 | A | 13 | 11-set-10 | 2 | *Monodelphis* | *glirina* | MOGL | 928 | M | 0 |
| CANGA 1 | B | 16 | 11-set-10 | 2 | *Monodelphis* | *glirina* | MOGL | 930 | F | 0 |
| CANGA 1 | D | 3 | 11-set-10 | 2 | *Monodelphis* | *glirina* | MOGL | 932 | F | 0 |
| CANGA 1 | C | 7 | 11-set-10 | 2 | *Monodelphis* | *glirina* | MOGL | . | M | 0 |
| CANGA 1 | B | 5 | 12-set-10 | 2 | *Monodelphis* | *glirina* | MOGL | 936 |  | 0 |
| CANGA 1 | A | 9 | 12-set-10 | 2 | *Monodelphis* | *glirina* | MOGL | 938 | F | 0 |
| CANGA 1 | E | 12 | 12-set-10 | 2 | *Monodelphis* | *glirina* | MOGL | 940 | M | 0 |
| CANGA 1 | A | 22 | 12-set-10 | 2 | *Monodelphis* | *glirina* | MOGL | 942 | F | 0 |
| CANGA 1 | D | 2 | 12-set-10 | 2 | *Monodelphis* | *glirina* | MOGL | 944 | F | 0 |
| CANGA 1 | D | 26 | 12-set-10 | 2 | *Monodelphis* | *glirina* | MOGL | 948 | M | 0 |
| FLORESTA 1 | B | 8 | 7-ago-10 | 2 | *Monodelphis* | *sp. D* | MOsp. | . | F | 0 |
| FLORESTA 1 | D | 2 | 7-ago-10 | 2 | *Monodelphis* | *sp. D* | MOsp. | 468 | F | 0 |
| FLORESTA 1 | D | 11 | 7-ago-10 | 2 | *Monodelphis* | *sp. D* | MOsp. | 466 | F | 0 |
| FLORESTA 1 | D | 10 | 7-ago-10 | 2 | *Monodelphis* | *sp. D* | MOsp. | . | M | 0 |
| FLORESTA 1 | B | 7 | 8-ago-10 | 2 | *Monodelphis* | *sp. D* | MOsp. | 478 | F | 0 |
| FLORESTA 1 | A | 13 | 8-ago-10 | 2 | *Monodelphis* | *sp. D* | MOsp. | 480 | M | 0 |
| FLORESTA 1 | C | 2 | 11-ago-10 | 2 | *Monodelphis* | *sp. D* | MOsp. | 484 | M | 0 |
| FLORESTA 1 | E | 10 | 11-ago-10 | 2 | *Monodelphis* | *sp. D* | MOsp. | 486 | F | 0 |
| FLORESTA 2 | G | 15 | 11-ago-10 | 2 | *Monodelphis* | *sp. D* | MOsp. | 488 | M | 0 |
| FLORESTA 1 | A | 10 | 13-ago-10 | 2 | *Monodelphis* | *sp. D* | MOsp. | 492 | F | 0 |
| FLORESTA 1 | A | 3 | 17-ago-10 | 2 | *Monodelphis* | *sp. D* | MOsp. | 496 | F | 0 |
| FLORESTA 1 | B | 52 | 18-ago-10 | 2 | *Monodelphis* | *sp. D* | MOsp. | 498 | M | 0 |
| FLORESTA 1 | B | 32 | 20-ago-10 | 2 | *Monodelphis* | *sp. D* | MOsp. | . | F | 0 |
| FLORESTA 1 | B | 19 | 21-ago-10 | 2 | *Monodelphis* | *sp. D* | MOsp. | 500 | M | 0 |
| FLORESTA 1 | A | 31 | 22-ago-10 | 2 | *Monodelphis* | *sp. D* | MOsp. | . |  | 0 |
| FLORESTA 2 | G | 27 | 24-ago-10 | 2 | *Monodelphis* | *sp. D* | MOsp. | 606 | F | 0 |
| FLORESTA 2 | G | 16 | 24-ago-10 | 2 | *Monodelphis* | *sp. D* | MOsp. | 608 | F | 0 |
| FLORESTA 2 | A | 28 | 26-ago-10 | 2 | *Monodelphis* | *sp. D* | MOsp. | 614 | F | 0 |
| FLORESTA 2 | G | 27 | 27-ago-10 | 2 | *Monodelphis* | *sp. D* | MOsp. | 618 | F | 0 |
| FLORESTA 2 | G | 15 | 6-ago-10 | 2 | *Neacomys* | *cf. paracou* | NEPA | . | M | 0 |
| FLORESTA 1 | D | 10 | 8-ago-10 | 2 | *Oecomys* | sp. | OECO | . | F | 0 |
| FLORESTA 2 | B | 15 | 8-ago-10 | 2 | *Oecomys* | sp. | OECO | . | M | 0 |
| FLORESTA 1 | D | 8 | 11-ago-10 | 2 | *Oecomys* | sp. | OECO | . | F | 0 |
| FLORESTA 1 | E | 14 | 13-ago-10 | 2 | *Oecomys* | sp. | OECO | . | M | 0 |
| FLORESTA 1 | G | 6 | 13-ago-10 | 2 | *Oecomys* | sp. | OECO | . | F | 0 |
| FLORESTA 1 | C | 14 | 18-ago-10 | 2 | *Oecomys* | sp. | OECO | . | F | 0 |
| FLORESTA 1 | A | 45 | 22-ago-10 | 2 | *Oecomys* | sp. | OECO | . |  | 0 |
| FLORESTA 2 | B | 50 | 26-ago-10 | 2 | *Oecomys* | sp. | OECO | . |  | 0 |
| CANGA 2 | C | 26 | 31-ago-10 | 2 | *Oxymycterus* | *amazonicus* | OXAM | 654 | F | 0 |
| CANGA 2 | C | 16 | 31-ago-10 | 2 | *Oxymycterus* | *amazonicus* | OXAM | . | M | 0 |
| CANGA 2 | B | 15 | 31-ago-10 | 2 | *Oxymycterus* | *amazonicus* | OXAM | . | M | 0 |
| CANGA 2 | C | 30 | 31-ago-10 | 2 | *Oxymycterus* | *amazonicus* | OXAM | 656 | M | 0 |
| CANGA 2 | C | 35 | 31-ago-10 | 2 | *Oxymycterus* | *amazonicus* | OXAM | 345 | F | 1 |
| CANGA 2 | E | 30 | 1-set-10 | 2 | *Oxymycterus* | *amazonicus* | OXAM | 710 | F | 0 |
| CANGA 2 | G | 34 | 1-set-10 | 2 | *Oxymycterus* | *amazonicus* | OXAM | 714 | M | 0 |
| CANGA 2 | E | 39 | 1-set-10 | 2 | *Oxymycterus* | *amazonicus* | OXAM | 712 | M | 0 |
| CANGA 2 | G | 44 | 1-set-10 | 2 | *Oxymycterus* | *amazonicus* | OXAM | 716 | F | 0 |
| CANGA 2 | C | 19 | 1-set-10 | 2 | *Oxymycterus* | *amazonicus* | OXAM | . | M | 0 |
| CANGA 2 | D | 35 | 2-set-10 | 2 | *Oxymycterus* | *amazonicus* | OXAM | 734 | M | 0 |
| CANGA 2 | C | 6 | 2-set-10 | 2 | *Oxymycterus* | *amazonicus* | OXAM | 742 | F | 0 |
| CANGA 2 | D | 16 | 2-set-10 | 2 | *Oxymycterus* | *amazonicus* | OXAM | 746 | F | 0 |
| CANGA 2 | B | 13 | 2-set-10 | 2 | *Oxymycterus* | *amazonicus* | OXAM | 750 | M | 0 |
| CANGA 2 | B | 35 | 2-set-10 | 2 | *Oxymycterus* | *amazonicus* | OXAM | 752 | M | 0 |
| CANGA 2 | C | 32 | 2-set-10 | 2 | *Oxymycterus* | *amazonicus* | OXAM | 756 | M | 0 |
| CANGA 2 | B | 9 | 2-set-10 | 2 | *Oxymycterus* | *amazonicus* | OXAM | 758 | M | 0 |
| CANGA 2 | G | 55 | 3-set-10 | 2 | *Oxymycterus* | *amazonicus* | OXAM | 768 | F | 0 |
| CANGA 2 | B | 35 | 3-set-10 | 2 | *Oxymycterus* | *amazonicus* | OXAM | 766 | F | 1 |
| CANGA 2 | D | 16 | 3-set-10 | 2 | *Oxymycterus* | *amazonicus* | OXAM | 770 | M | 0 |
| CANGA 2 | D | 19 | 3-set-10 | 2 | *Oxymycterus* | *amazonicus* | OXAM | 762 | F | 0 |
| CANGA 2 | B | 4 | 3-set-10 | 2 | *Oxymycterus* | *amazonicus* | OXAM | 772 | F | 0 |
| CANGA 2 | C | 16 | 3-set-10 | 2 | *Oxymycterus* | *amazonicus* | OXAM | 778 | M | . |
| CANGA 2 | D | 16 | 4-set-10 | 2 | *Oxymycterus* | *amazonicus* | OXAM | 798 | M | 0 |
| CANGA 2 | A | 26 | 4-set-10 | 2 | *Oxymycterus* | *amazonicus* | OXAM | 800 | M | 0 |
| CANGA 2 | D | 3 | 4-set-10 | 2 | *Oxymycterus* | *amazonicus* | OXAM | . | F | 0 |
| CANGA 2 | D | 32 | 5-set-10 | 2 | *Oxymycterus* | *amazonicus* | OXAM | 814 | F | 0 |
| CANGA 2 | C | 10 | 5-set-10 | 2 | *Oxymycterus* | *amazonicus* | OXAM | 812 | M | 0 |
| CANGA 2 | B | 4 | 5-set-10 | 2 | *Oxymycterus* | *amazonicus* | OXAM | 804 | F | 0 |
| CANGA 2 | C | 26 | 5-set-10 | 2 | *Oxymycterus* | *amazonicus* | OXAM | . | M | 0 |
| CANGA 2 | G | 25 | 5-set-10 | 2 | *Oxymycterus* | *amazonicus* | OXAM | 808 | M | 0 |
| CANGA 1 | B | 1 | 7-set-10 | 2 | *Oxymycterus* | *amazonicus* | OXAM | 834 | M | 0 |
| CANGA 1 | B | 15 | 7-set-10 | 2 | *Oxymycterus* | *amazonicus* | OXAM | 836 | F | 0 |
| CANGA 1 | D | 2 | 7-set-10 | 2 | *Oxymycterus* | *amazonicus* | OXAM | 840 | M | 0 |
| CANGA 1 | E | 13 | 7-set-10 | 2 | *Oxymycterus* | *amazonicus* | OXAM | 838 | M | 0 |
| CANGA 1 | E | 3 | 7-set-10 | 2 | *Oxymycterus* | *amazonicus* | OXAM | . | F | 0 |
| CANGA 1 | C | 8 | 7-set-10 | 2 | *Oxymycterus* | *amazonicus* | OXAM | . | M | 0 |
| CANGA 2 | A | 18 | 5-set-10 | 2 | *Oxymycterus* | *amazonicus* | OXAM | . | M | 0 |
| CANGA 2 | C | 4 | 5-set-10 | 2 | *Oxymycterus* | *amazonicus* | OXAM | . | F | 0 |
| CANGA 2 | D | 26 | 5-set-10 | 2 | *Oxymycterus* | *amazonicus* | OXAM | . | M | 0 |
| CANGA 2 | D | 19 | 5-set-10 | 2 | *Oxymycterus* | *amazonicus* | OXAM | . | F | 0 |
| CANGA 1 | A | 42 | 8-set-10 | 2 | *Oxymycterus* | *amazonicus* | OXAM | 854 | M | 0 |
| CANGA 1 | E | 16 | 8-set-10 | 2 | *Oxymycterus* | *amazonicus* | OXAM | 856 | M | 0 |
| CANGA 1 | E | 9 | 8-set-10 | 2 | *Oxymycterus* | *amazonicus* | OXAM | 860 | F | 0 |
| CANGA 1 | E | 17 | 8-set-10 | 2 | *Oxymycterus* | *amazonicus* | OXAM | 864 | F | 0 |
| CANGA 1 | B | 28 | 8-set-10 | 2 | *Oxymycterus* | *amazonicus* | OXAM | 870 | M | 0 |
| CANGA 1 | D | 17 | 9-set-10 | 2 | *Oxymycterus* | *amazonicus* | OXAM | 872 | M | 0 |
| CANGA 1 | E | 9 | 9-set-10 | 2 | *Oxymycterus* | *amazonicus* | OXAM | 876 | M | 0 |
| CANGA 1 | A | 12 | 9-set-10 | 2 | *Oxymycterus* | *amazonicus* | OXAM | 878 | F | 0 |
| CANGA 1 | E | . | 9-set-10 | 2 | *Oxymycterus* | *amazonicus* | OXAM | 884 | F | 0 |
| CANGA 1 | A | 44 | 9-set-10 | 2 | *Oxymycterus* | *amazonicus* | OXAM | 892 | M | 0 |
| CANGA 1 | A | 8 | 10-set-10 | 2 | *Oxymycterus* | *amazonicus* | OXAM | 908 | M | 0 |
| CANGA 1 | B | 15 | 10-set-10 | 2 | *Oxymycterus* | *amazonicus* | OXAM | 910 | F | 0 |
| CANGA 1 | C | 9 | 11-set-10 | 2 | *Oxymycterus* | *amazonicus* | OXAM | 924 | M | 0 |
| CANGA 1 | E | 5 | 12-set-10 | 2 | *Oxymycterus* | *amazonicus* | OXAM | 958 | M | 0 |
| CANGA 1 | B | 20 | 12-set-10 | 2 | *Oxymycterus* | *amazonicus* | OXAM | 962 | M | 0 |
| FLORESTA 1 | B | 37 | 22-ago-10 | 2 | *Philander* | *opossum* | PHOP | 604 | M | 0 |
| FLORESTA 1 | B | 14 | 7-ago-10 | 2 | *Proechimys* | *roberti* | PRRO | 472 | F | 0 |
| FLORESTA 1 | D | 38 | 18-ago-10 | 2 | *Proechimys* | *roberti* | PRRO | . |  | 0 |
| FLORESTA 2 | D | 19 | 29-ago-10 | 2 | *Proechimys* | *roberti* | PRRO | 652 | F | 0 |
| CANGA 2 | A | 26 | 3-set-10 | 2 | *Proechimys* | *roberti* | PRRO | . | F | 0 |
| CANGA 2 | C | 50 | 5-set-10 | 2 | *Rhipidomys* | *emiliae* | RHEM | . | F | 0 |
| Floresta 2 | D | 28 | 20-jan-11 | 3 | *Proechimys* | *roberti* | PRRO |  |  | 0 |
| Floresta 2 | B | 13 | 20-jan-11 | 3 | *Monodelphis* | *glirina* | MOGL | 618 |  | 0 |
| Floresta 2 | D | 51 | 20-jan-11 | 3 | *Proechimys* | *roberti* | PRRO |  | F | 1 |
| Floresta 2 | B | 25 | 20-jan-11 | 3 | *Didelphis* | *marsupialis* | DIMA |  | M | 0 |
| Floresta 2 | B | 29 | 22-jan-11 | 3 | *Monodelphis* | *glirina* | MOGL |  | F | 0 |
| Floresta 2 | A | 20 | 22-jan-11 | 3 | *Monodelphis* | *sp. D* | MOsp. |  | F | 0 |
| Floresta 2 | C | 16 | 23-jan-11 | 3 | *Metachirus* | *nudicaudatus* | MENU |  | M | 0 |
| Floresta 2 | G | 28 | 23-jan-11 | 3 | *Marmosa* | *murina* | MAMU |  | F | 0 |
| Floresta 2 | C | 33 | 23-jan-11 | 3 | *Monodelphis* | *glirina* | MOGL | 618 | F | 0 |
| Floresta 2 | C | 50 | 23-jan-11 | 3 | *Philander* | *opossum* | PHOP |  | F | 1 |
| Floresta 2 | A | 2 | 23-jan-11 | 3 | *Caluromys* | *philander* | CAPH |  | F | 0 |
| Floresta 2 | C | 6 | 23-jan-11 | 3 | *Marmosa* | *murina* | MAMU |  | F | 0 |
| Floresta 2 | D | 4 | 24-jan-11 | 3 | *Oecomys* | sp. | OECO |  | M | 0 |
| Floresta 2 | B | 14 | 25-jan-11 | 3 | *Monodelphis* | *aff. kunsi* | MOKU |  | F | 0 |
| Floresta 2 | E | 4 | 25-jan-11 | 3 | *Marmosa* | *murina* | MAMU |  | M | 0 |
| Floresta 2 | A | 6 | 25-jan-11 | 3 | *Proechimys* | *roberti* | PRRO |  |  |  |
| Floresta 2 | G | 6 | 25-jan-11 | 3 | *Marmosa* | *demerarae* | MADE |  | F | 0 |
| Floresta 2 | B | 6 | 25-jan-11 | 3 | *Monodelphis* | *glirina* | MOGL |  | M | 0 |
| Floresta 2 | B | 1 | 25-jan-11 | 3 | *Monodelphis* | *glirina* | MOGL |  | F | 0 |
| Floresta 2 | C | 35 | 25-jan-11 | 3 | *Marmosa* | *demerarae* | MADE | 801 | F | 0 |
| Floresta 2 | C | 21 | 25-jan-11 | 3 | *Oxymycterus* | *amazonicus* | OXAM |  | F | 0 |
| Floresta 2 | C | 2 | 25-jan-11 | 3 | *Rhipidomys* | *emiliae* | RHEM |  |  |  |
| Canga 1 | C | 9 | 27-jan-11 | 3 | *Monodelphis* | *glirina* | MOGL |  | M | 0 |
| Canga 1 | B | 11 | 27-jan-11 | 3 | *Monodelphis* | *glirina* | MOGL |  | M | 0 |
| Canga 1 | B | 14 | 27-jan-11 | 3 | *Monodelphis* | *glirina* | MOGL | 803 | M |  |
| Canga 1 | C | 2 | 27-jan-11 | 3 | *Monodelphis* | *glirina* | MOGL | 805 | F | 0 |
| Canga 1 | E | 9 | 27-jan-11 | 3 | *Monodelphis* | *glirina* | MOGL | 807 | F | 0 |
| Canga 1 | B | 6 | 27-jan-11 | 3 | *Monodelphis* | *glirina* | MOGL | 809 | F | 0 |
| Canga 1 | C | 2 | 27-jan-11 | 3 | *Monodelphis* | *glirina* | MOGL | 942 | M | 0 |
| Canga 1 | C | 11 | 27-jan-11 | 3 | *Monodelphis* | *glirina* | MOGL | 811 |  | 1 |
| Canga 1 | E | 11 | 27-jan-11 | 3 | *Monodelphis* | *glirina* | MOGL | 904 | M | 0 |
| Canga 1 | D | 9 | 27-jan-11 | 3 | *Monodelphis* | *glirina* | MOGL | 813 | F | 1 |
| Canga 1 | G | 8 | 27-jan-11 | 3 | *Marmosa* | *demerarae* | MADE | 815 | F | 0 |
| Canga 1 | E | 13 | 27-jan-11 | 3 | *Monodelphis* | *glirina* | MOGL | 817 | M | 0 |
| Canga 1 | E | 2 | 27-jan-11 | 3 | *Monodelphis* | *glirina* | MOGL |  | M | 0 |
| Canga 1 | G | 7 | 27-jan-11 | 3 | *Monodelphis* | *glirina* | MOGL |  |  | 0 |
| Canga 1 | G | 7 | 27-jan-11 | 3 | *Monodelphis* | *glirina* | MOGL | 819 | F | 0 |
| Canga 1 | G | 2 | 27-jan-11 | 3 | *Proechimys* | *roberti* | PRRO | 821 | F | 0 |
| Canga 1 | G | 2 | 27-jan-11 | 3 | *Monodelphis* | *glirina* | MOGL | 823 | F | 0 |
| Canga 1 | C | 9 | 27-jan-11 | 3 | *Monodelphis* | *glirina* | MOGL |  | F | 0 |
| Canga 1 | E | 3 | 27-jan-11 | 3 | *Monodelphis* | *glirina* | MOGL | 825 |  | 0 |
| Canga 1 | G | 12 | 27-jan-11 | 3 | *Monodelphis* | *glirina* | MOGL | 827 | M | 0 |
| Canga 1 | B | 7 | 27-jan-11 | 3 | *Oxymycterus* | *amazonicus* | OXAM | 829 | M | 0 |
| Canga 1 | B | 6 | 27-jan-11 | 3 | *Monodelphis* | *glirina* | MOGL | 831 | F | 0 |
| Canga 1 | C | 1 | 27-jan-11 | 3 | *Monodelphis* | *glirina* | MOGL |  | F | 0 |
| Canga 1 | G | 6 | 27-jan-11 | 3 | *Oxymycterus* | *amazonicus* | OXAM | 880 |  | 0 |
| Canga 1 | C | 5 | 28-jan-11 | 3 | *Monodelphis* | *glirina* | MOGL | 833 | M | 1 |
| Canga 1 | G | 9 | 28-jan-11 | 3 | *Monodelphis* | *glirina* | MOGL |  | F | 0 |
| Canga 1 | C | 3 | 28-jan-11 | 3 | *Monodelphis* | *glirina* | MOGL | 837 |  | 0 |
| Canga 1 | A | 5 | 28-jan-11 | 3 | *Oxymycterus* | *amazonicus* | OXAM | 863 |  | 0 |
| Canga 1 | D | 5 | 28-jan-11 | 3 | *Oxymycterus* | *amazonicus* | OXAM | 847 | F | 0 |
| Canga 1 | G | 12 | 28-jan-11 | 3 | *Monodelphis* | *glirina* | MOGL | 859 | M | 0 |
| Canga 1 | G | 11 | 28-jan-11 | 3 | *Monodelphis* | *glirina* | MOGL | 853 | F | 0 |
| Canga 1 | G | 12 | 28-jan-11 | 3 | *Monodelphis* | *glirina* | MOGL | 114 | M | 0 |
| Canga 1 | G | 12 | 28-jan-11 | 3 | *Oxymycterus* | *amazonicus* | OXAM | 855 | M | 1 |
| Canga 1 | B | 11 | 28-jan-11 | 3 | *Monodelphis* | *glirina* | MOGL | 839 | F | 0 |
| Canga 1 | A | 6 | 28-jan-11 | 3 | *Monodelphis* | *glirina* | MOGL |  | M | 0 |
| Canga 1 | G | 14 | 28-jan-11 | 3 | *Monodelphis* | *glirina* | MOGL | 857 | M | 0 |
| Canga 1 | A | 3 | 28-jan-11 | 3 | *Monodelphis* | *glirina* | MOGL | 851 | F | 0 |
| Canga 1 | A | 6 | 28-jan-11 | 3 | *Monodelphis* | *glirina* | MOGL | 849 | F | 0 |
| Canga 1 | C | 14 | 28-jan-11 | 3 | *Monodelphis* | *glirina* | MOGL | 835 | F | 0 |
| Canga 1 | A | 6 | 28-jan-11 | 3 | *Monodelphis* | *glirina* | MOGL | 841 | M | 0 |
| Canga 1 | A | 6 | 28-jan-11 | 3 | *Oxymycterus* | *amazonicus* | OXAM | 845 | F | 0 |
| Canga 1 | G | 2 | 28-jan-11 | 3 | *Proechimys* | *roberti* | PRRO | 843 | F | 0 |
| Canga 1 | E | 12 | 28-jan-11 | 3 | *Monodelphis* | *glirina* | MOGL |  | M | 0 |
| Canga 1 | D | 14 | 29-jan-11 | 3 | *Monodelphis* | *glirina* | MOGL |  | F | 0 |
| Canga 1 | B | 4 | 29-jan-11 | 3 | *Monodelphis* | *glirina* | MOGL | 886 | M | 0 |
| Canga 1 | C | 6 | 29-jan-11 | 3 | *Necromys* | *lasiurus* | NELA | 803 | M | 1 |
| Canga 1 | D | 14 | 29-jan-11 | 3 | *Monodelphis* | *glirina* | MOGL | 865 | F | 1 |
| Canga 1 | D | 11 | 29-jan-11 | 3 | *Monodelphis* | *glirina* | MOGL | 867 | F | 0 |
| Canga 1 | E | 15 | 29-jan-11 | 3 | *Monodelphis* | *glirina* | MOGL | 869 | M | 0 |
| Canga 1 | G | 12 | 29-jan-11 | 3 | *Monodelphis* | *glirina* | MOGL | 871 | M | 0 |
| Canga 1 | G | 6 | 29-jan-11 | 3 | *Monodelphis* | *glirina* | MOGL | 873 | M | 0 |
| Canga 1 | D | 9 | 29-jan-11 | 3 | *Monodelphis* | *glirina* | MOGL | 831 | M | 0 |
| Canga 1 | G | 14 | 29-jan-11 | 3 | *Monodelphis* | *glirina* | MOGL | 875 | F | 1 |
| Canga 1 | E | 14 | 29-jan-11 | 3 | *Necromys* | *lasiurus* | NELA | 877 | F | 0 |
| Canga 1 | D | 2 | 29-jan-11 | 3 | *Marmosa* | *murina* | MAMU | 843 |  | 0 |
| Canga 1 | D | 38 | 29-jan-11 | 3 | *Monodelphis* | *glirina* | MOGL | 879 | M | 1 |
| Canga 1 | G | 27 | 29-jan-11 | 3 | *Monodelphis* | *glirina* | MOGL |  | M | 0 |
| Canga 1 | E | 31 | 29-jan-11 | 3 | *Necromys* | *lasiurus* | NELA | 880 | F | 0 |
| Canga 1 | A | 17 | 29-jan-11 | 3 | *Monodelphis* | *glirina* | MOGL | 918 | M | 1 |
| Floresta 1 | B | 33 | 20-fev-11 | 3 | *Oecomys* | sp. | OECO |  | M | 1 |
| Floresta 1 | E | 8 | 20-fev-11 | 3 | *Oecomys* | sp. | OECO | 928 | M | 0 |
| Floresta 1 | B | 14 | 20-fev-11 | 3 | *Oecomys* | sp. | OECO |  | M | 1 |
| Floresta 1 | A | 34 | 20-fev-11 | 3 | *Oecomys* | sp. | OECO | 45 |  | 0 |
| Floresta 1 | G | 41 | 20-fev-11 | 3 | *Akodon* | *aff. cursor* | AKCU | 46 | F | 0 |
| Floresta 1 | A | 35 | 20-fev-11 | 3 | *Euryoryzomys* | *emmonsae* | EUEM | 44 | M | 0 |
| Floresta 1 | D | 3 | 20-fev-11 | 3 | *Oecomys* | sp. | OECO |  | M | 0 |
| Floresta 1 | D | 38 | 20-fev-11 | 3 | *Oecomys* | sp. | OECO |  |  | 0 |
| Floresta 1 | D | 28 | 20-fev-11 | 3 | *Oecomys* | sp. | OECO | 29 |  | 0 |
| Floresta 1 | G | 44 | 20-fev-11 | 3 | *Monodelphis* | *sp. D* | MOsp. | 30 | M | 0 |
| Floresta 1 | B | 38 | 20-fev-11 | 3 | *Oecomys* | sp. | OECO | 27 | M | 0 |
| Floresta 1 | B | 45 | 20-fev-11 | 3 | *Neacomys* | *cf. paracou* | NEPA | 28 | F | 0 |
| Floresta 1 | C | 51 | 20-fev-11 | 3 | *Akodon* | *aff. cursor* | AKCU | 25 | M | 0 |
| Floresta 2 | B | 52 | 20-fev-11 | 3 | *Hylaeamys* | *megacephalus* | HYME | 26 | F | 0 |
| Floresta 2 | G | 59 | 20-fev-11 | 3 | *Proechimys* | *roberti* | PRRO | 49 | F | 0 |
| Floresta 2 | B | 6 | 20-fev-11 | 3 | *Marmosops* | *pinheiroi* | MAPI |  | M | 0 |
| Floresta 2 | E | 11 | 20-fev-11 | 3 | *Monodelphis* | *glirina* | MOGL | 587 |  | 0 |
| Floresta 2 | A | 26 | 20-fev-11 | 3 | *Oecomys* | sp. | OECO |  |  | 1 |
| Floresta 2 | B | 45 | 20-fev-11 | 3 | *Euryoryzomys* | *emmonsae* | EUEM | 801 |  |  |
| Floresta 2 | E | 7 | 20-fev-11 | 3 | *Monodelphis* | *sp. D* | MOsp. | 18 | F |  |
| Floresta 2 | G | 8 | 20-fev-11 | 3 | *Monodelphis* | *glirina* | MOGL |  | F | 0 |
| Floresta 2 | E | 12 | 20-fev-11 | 3 | *Oecomys* | sp. | OECO | 17 | F | 0 |
| Floresta 2 | D | 7 | 20-fev-11 | 3 | *Oecomys* | sp. | OECO | 19 | F | 1 |
| Floresta 2 | D | 10 | 20-fev-11 | 3 | *Euryoryzomys* | *emmonsae* | EUEM | 20 | F | 0 |
| Floresta 2 | E | 6 | 20-fev-11 | 3 | *Marmosops* | *pinheiroi* | MAPI | 15 | M | 0 |
| Floresta 2 | B | 13 | 20-fev-11 | 3 | *Oecomys* | sp. | OECO | 16 | M | 0 |
| Floresta 2 | G | 2 | 20-fev-11 | 3 | *Neacomys* | *cf. paracou* | NEPA |  | M | 0 |
| Floresta 2 | B | 6 | 20-fev-11 | 3 | *Oecomys* | sp. | OECO |  |  |  |
| Floresta 2 | D | 12 | 20-fev-11 | 3 | *Monodelphis* | *sp. D* | MOsp. | 13 |  |  |
| Floresta 2 | A | 4 | 20-fev-11 | 3 | *Euryoryzomys* | *emmonsae* | EUEM | 14 | F | 0 |
| Floresta 2 | E | 10 | 20-fev-11 | 3 | *Euryoryzomys* | *emmonsae* | EUEM | 24 | M | 0 |
| Floresta 2 | D | 15 | 20-fev-11 | 3 | *Neacomys* | *cf. paracou* | NEPA | 12 | F | 0 |
| Floresta 2 | A | 13 | 20-fev-11 | 3 | *Neacomys* | *cf. paracou* | NEPA | 23 |  | 0 |
| Floresta 1 | D | 15 | 21-fev-11 | 3 | *Oecomys* | sp. | OECO |  | M | 0 |
| Floresta 1 | D | 6 | 21-fev-11 | 3 | *Oecomys* | sp. | OECO | 21 |  |  |
| Floresta 1 | G | 9 | 21-fev-11 | 3 | *Oecomys* | sp. | OECO |  | F | 0 |
| Floresta 1 | E | 15 | 21-fev-11 | 3 | *Oxymycterus* | *amazonicus* | OXAM |  | F |  |
| Floresta 1 | G | 2 | 21-fev-11 | 3 | *Akodon* | *aff. cursor* | AKCU |  |  | 0 |
| Floresta 1 |  | . | 21-fev-11 | 3 | *Oxymycterus* | *amazonicus* | OXAM |  |  | 0 |
| Floresta 1 | E | 15 | 21-fev-11 | 3 | *Neacomys* | *cf. paracou* | NEPA |  |  | 0 |
| Floresta 1 | B | 6 | 21-fev-11 | 3 | *Akodon* | *aff. cursor* | AKCU | 50 |  | 0 |
| Floresta 1 | A | 3 | 21-fev-11 | 3 | *Akodon* | *aff. cursor* | AKCU | 51 | M | 0 |
| Floresta 1 | E | 10 | 21-fev-11 | 3 | *Oxymycterus* | *amazonicus* | OXAM | 52 | M | 0 |
| Floresta 1 | B | 1 | 21-fev-11 | 3 | *Monodelphis* | *sp. D* | MOsp. | 53 | M | 0 |
| Floresta 1 | E | 13 | 21-fev-11 | 3 | *Monodelphis* | *sp. D* | MOsp. | 55 | M | 0 |
| Floresta 1 | G | 27 | 21-fev-11 | 3 | *Oecomys* | sp. | OECO | 54 | F | 0 |
| Floresta 1 | G | 10 | 21-fev-11 | 3 | *Neacomys* | *cf. paracou* | NEPA | 56 | M | 0 |
| Floresta 1 | D | 59 | 21-fev-11 | 3 | *Didelphis* | *marsupialis* | DIMA | 57 | M | 0 |
| Floresta 1 | G | 9 | 21-fev-11 | 3 | *Oecomys* | sp. | OECO |  | M |  |
| Floresta 1 | G | 27 | 21-fev-11 | 3 | *Neacomys* | *cf. paracou* | NEPA | 59 | M | 0 |
| Floresta 1 | G | 14 | 21-fev-11 | 3 | *Oecomys* | sp. | OECO |  | M | 0 |
| Floresta 1 | G | 28 | 21-fev-11 | 3 | *Oecomys* | sp. | OECO | 44 |  |  |
| Floresta 1 | B | 17 | 21-fev-11 | 3 | *Marmosops* | *pinheiroi* | MAPI | 60 |  | 1 |
| Floresta 1 | G | 11 | 21-fev-11 | 3 | *Oecomys* | sp. | OECO | 61 | M | 0 |
| Floresta 2 | A | 27 | 21-fev-11 | 3 | *Oecomys* | sp. | OECO | 62 | F | 0 |
| Floresta 2 | B | 2 | 21-fev-11 | 3 | *Euryoryzomys* | *emmonsae* | EUEM | 63 | F | 0 |
| Floresta 2 | C | 23 | 21-fev-11 | 3 | *Euryoryzomys* | *emmonsae* | EUEM | 65 | M | 0 |
| Floresta 2 | A | 26 | 21-fev-11 | 3 | *Euryoryzomys* | *emmonsae* | EUEM |  | F | 0 |
| Floresta 2 | A | 22 | 21-fev-11 | 3 | *Euryoryzomys* | *emmonsae* | EUEM |  |  |  |
| Floresta 2 | A | 10 | 21-fev-11 | 3 | *Euryoryzomys* | *emmonsae* | EUEM |  |  |  |
| Floresta 2 | A | 17 | 21-fev-11 | 3 | *Euryoryzomys* | *emmonsae* | EUEM |  |  |  |
| Floresta 2 | A | 23 | 21-fev-11 | 3 | *Oecomys* | sp. | OECO |  |  |  |
| Floresta 2 | C | 5 | 21-fev-11 | 3 | *Euryoryzomys* | *emmonsae* | EUEM |  |  |  |
| Floresta 2 | A | 20 | 21-fev-11 | 3 | *Euryoryzomys* | *emmonsae* | EUEM |  |  |  |
| Floresta 2 | A | 32 | 21-fev-11 | 3 | *Euryoryzomys* | *emmonsae* | EUEM |  |  |  |
| Floresta 2 | B | 31 | 21-fev-11 | 3 | *Oxymycterus* | *amazonicus* | OXAM |  |  |  |
| Floresta 2 | B | 20 | 21-fev-11 | 3 | *Marmosops* | *pinheiroi* | MAPI |  |  |  |
| Floresta 2 | B | 24 | 21-fev-11 | 3 | *Oecomys* | sp. | OECO |  |  |  |
| Floresta 1 | E | 24 | 21-fev-11 | 3 | *Marmosops* | *pinheiroi* | MAPI |  |  |  |
| Floresta 1 | E | 23 | 21-fev-11 | 3 | *Monodelphis* | *sp. D* | MOsp. |  |  |  |
| Floresta 1 | E | 18 | 21-fev-11 | 3 | *Oecomys* | sp. | OECO |  |  |  |
| Floresta 1 | E | 9 | 21-fev-11 | 3 | *Neacomys* | *cf. paracou* | NEPA |  |  |  |
| Floresta 1 | D | 5 | 21-fev-11 | 3 | *Oecomys* | sp. | OECO | 64 |  |  |
| Floresta 1 | A | 30 | 21-fev-11 | 3 | *Oecomys* | sp. | OECO | 66 | M | 0 |
| Floresta 1 | A | 21 | 21-fev-11 | 3 | *Oecomys* | sp. | OECO | 67 | F | 0 |
| Floresta 1 | D | 21 | 21-fev-11 | 3 | *Oecomys* | sp. | OECO | 68 | F | 0 |
| Floresta 1 | B | 16 | 21-fev-11 | 3 | *Oecomys* | sp. | OECO | 69 | F | 0 |
| Floresta 2 | A | 34 | 21-fev-11 | 3 | *Oecomys* | sp. | OECO | 70 | M | 0 |
| Floresta 2 | C | 3 | 21-fev-11 | 3 | *Euryoryzomys* | *emmonsae* | EUEM | 71 | F | 0 |
| Floresta 1 | C | 21 | 21-fev-11 | 3 | *Oecomys* | sp. | OECO | 72 | F | 0 |
| Floresta 2 | D | 30 | 21-fev-11 | 3 | *Oecomys* | sp. | OECO | 73 | M |  |
| Floresta 1 | C | 27 | 21-fev-11 | 3 | *Oecomys* | sp. | OECO | 74 | M | 0 |
| Floresta 2 | C | 31 | 21-fev-11 | 3 | *Euryoryzomys* | *emmonsae* | EUEM | 76 | F | 0 |
| Floresta 1 | C | 30 | 21-fev-11 | 3 | *Proechimys* | *roberti* | PRRO |  | M | 0 |
| Floresta 1 | E | 21 | 21-fev-11 | 3 | *Oecomys* | sp. | OECO | 77 | F |  |
| Floresta 1 | C | 11 | 21-fev-11 | 3 | *Oecomys* | sp. | OECO | 78 | M | 0 |
| Floresta 2 | B | 8 | 21-fev-11 | 3 | *Euryoryzomys* | *emmonsae* | EUEM | 79 | M | 0 |
| Floresta 2 | C | 21 | 21-fev-11 | 3 | *Oecomys* | sp. | OECO | 80 | M | 0 |
| Floresta 2 | A | 10 | 21-fev-11 | 3 | *Oecomys* | sp. | OECO | 81 | F | 0 |
| Floresta 2 | E | 24 | 21-fev-11 | 3 | *Euryoryzomys* | *emmonsae* | EUEM | 82 | M | 0 |
| Floresta 2 | E | 25 | 21-fev-11 | 3 | *Akodon* | *aff. cursor* | AKCU | 83 | F | 0 |
| Floresta 2 | E | 20 | 21-fev-11 | 3 | *Oecomys* | sp. | OECO |  | M | 0 |
| Floresta 2 | D | 5 | 21-fev-11 | 3 | *Oxymycterus* | *amazonicus* | OXAM | 84 | M | 0 |
| Floresta 2 | E | 3 | 21-fev-11 | 3 | *Oxymycterus* | *amazonicus* | OXAM | 85 | F | 0 |
| Floresta 2 | C | 3 | 21-fev-11 | 3 | *Oxymycterus* | *amazonicus* | OXAM | 86 | F | 0 |
| Floresta 2 | D | 11 | 21-fev-11 | 3 | *Marmosa* | *demerarae* | MADE | 23 | M | 0 |
| Floresta 2 | C | 19 | 21-fev-11 | 3 | *Euryoryzomys* | *emmonsae* | EUEM | 87 | M | 1 |
| Floresta 2 | C | 5 | 21-fev-11 | 3 | *Oecomys* | sp. | OECO | 88 | M | 0 |
| Floresta 2 | B | 36 | 21-fev-11 | 3 | *Marmosops* | *pinheiroi* | MAPI | 89 | M | 0 |
| Floresta 2 | A | 29 | 21-fev-11 | 3 | *Monodelphis* | *glirina* | MOGL | 90 | M | 0 |
| Floresta 2 | C | 11 | 21-fev-11 | 3 | *Marmosops* | *pinheiroi* | MAPI | 91 | F | 0 |
| Floresta 2 | D | 18 | 21-fev-11 | 3 | *Marmosops* | *pinheiroi* | MAPI | 92 | M | 0 |
| Floresta 2 | D | 17 | 21-fev-11 | 3 | *Monodelphis* | *sp. D* | MOsp. | 93 | M | 0 |
| Floresta 2 | B | 1 | 22-fev-11 | 3 | *Oecomys* | sp. | OECO | 94 |  | 0 |
| Floresta 2 | D | 17 | 22-fev-11 | 3 | *Marmosops* | *pinheiroi* | MAPI | 95 | M | 0 |
| Floresta 2 | E | 19 | 22-fev-11 | 3 | *Euryoryzomys* | *emmonsae* | EUEM | 96 | F | 0 |
| Floresta 2 | E | 21 | 22-fev-11 | 3 | *Euryoryzomys* | *emmonsae* | EUEM | 97 | M | 0 |
| Floresta 2 | E | 7 | 22-fev-11 | 3 | *Euryoryzomys* | *emmonsae* | EUEM | 98 | M | 0 |
| Floresta 1 | C | 11 | 22-fev-11 | 3 | *Oecomys* | sp. | OECO | 99 | M | 0 |
| Floresta 1 | A | 14 | 22-fev-11 | 3 | *Euryoryzomys* | *emmonsae* | EUEM | 99 | F | 0 |
| Floresta 1 | A | 14 | 22-fev-11 | 3 | *Oecomys* | sp. | OECO |  | M | 0 |
| Floresta 2 | C | 11 | 22-fev-11 | 3 | *Oecomys* | sp. | OECO |  |  |  |
| Floresta 1 | G | 34 | 22-fev-11 | 3 | *Oecomys* | sp. | OECO |  |  |  |
| Floresta 2 | E | 8 | 22-fev-11 | 3 | *Oecomys* | sp. | OECO |  |  |  |
| Floresta 2 | G | 12 | 22-fev-11 | 3 | *Euryoryzomys* | *emmonsae* | EUEM |  |  |  |
| Floresta 1 | E | 47 | 22-fev-11 | 3 | *Oecomys* | sp. | OECO |  |  |  |
| Floresta 2 | E | 2 | 22-fev-11 | 3 | *Oecomys* | sp. | OECO |  |  |  |
| Floresta 2 | E | 43 | 22-fev-11 | 3 | *Marmosops* | *pinheiroi* | MAPI |  |  |  |
| Floresta 1 | G | 17 | 22-fev-11 | 3 | *Oecomys* | sp. | OECO |  |  |  |
| Floresta 2 | C | 48 | 22-fev-11 | 3 | *Euryoryzomys* | *emmonsae* | EUEM |  |  |  |
| Floresta 1 | E | 32 | 22-fev-11 | 3 | *Oecomys* | sp. | OECO |  |  |  |
| Floresta 2 | D | 27 | 22-fev-11 | 3 | *Euryoryzomys* | *emmonsae* | EUEM |  |  |  |
| Floresta 1 | E | 6 | 22-fev-11 | 3 | *Oxymycterus* | *amazonicus* | OXAM |  |  |  |
| Floresta 2 | A | 7 | 22-fev-11 | 3 | *Oecomys* | sp. | OECO |  |  |  |
| Floresta 1 | D | 50 | 22-fev-11 | 3 | *Monodelphis* | *sp. D* | MOsp. | 100 |  |  |
| Floresta 2 | C | 20 | 22-fev-11 | 3 | *Oecomys* | sp. | OECO |  | F | 0 |
| Floresta 1 | G | 36 | 22-fev-11 | 3 | *Proechimys* | *roberti* | PRRO | 102 | F | 0 |
| Floresta 2 | A | 42 | 22-fev-11 | 3 | *Rhipidomys* | *emiliae* | RHEM | 103 | M | 0 |
| Floresta 1 | B | 23 | 22-fev-11 | 3 | *Oecomys* | sp. | OECO | 104 | F | 0 |
| Floresta 2 | D | 32 | 22-fev-11 | 3 | *Oecomys* | sp. | OECO | 105 | M | 0 |
| Floresta 1 | C | 5 | 22-fev-11 | 3 | *Oecomys* | sp. | OECO | 106 | M | 0 |
| Floreta 2 | B | 50 | 22-fev-11 | 3 | *Oecomys* | sp. | OECO | 51 | M | 0 |
| FLORESTA 1 | A | 36 | 22-fev-11 | 3 | *Oxymycterus* | *amazonicus* | OXAM |  |  | 1 |
| Floresta 2 | G | 22 | 22-fev-11 | 3 | *Oxymycterus* | *amazonicus* | OXAM | 990 | M | 0 |
| Floresta 1 | A | 39 | 22-fev-11 | 3 | *Akodon* | *aff. cursor* | AKCU |  | F | 0 |
| Floresta 2 | G | 57 | 22-fev-11 | 3 | *Marmosops* | *pinheiroi* | MAPI | 108 |  |  |
| Floresta 1 | D | 36 | 22-fev-11 | 3 | *Oecomys* | sp. | OECO | 109 | F | 0 |
| Floresta 2 | G | 26 | 22-fev-11 | 3 | *Oecomys* | sp. | OECO | 110 | F | 0 |
| Floresta 1 | A | 21 | 22-fev-11 | 3 | *Monodelphis* | *sp. D* | MOsp. | 107 | F | 0 |
| Floresta 2 | C | 20 | 22-fev-11 | 3 | *Monodelphis* | *glirina* | MOGL | 111 | F | 0 |
| Floresta 1 | G | 57 | 22-fev-11 | 3 | *Monodelphis* | *sp. D* | MOsp. | 112 | F | 0 |
| ? | G | 56 | 22-fev-11 | 3 | *Oecomys* | sp. | OECO | 113 | F | 0 |
| Floresta 2 | E | 39 | 22-fev-11 | 3 | *Oecomys* | sp. | OECO | 114 | F | 0 |
| Floresta 2 | E | 38 | 22-fev-11 | 3 | *Euryoryzomys* | *emmonsae* | EUEM | 115 | F | 0 |
| Floresta 2 | E | 5 | 22-fev-11 | 3 | *Oecomys* | sp. | OECO |  | F | 0 |
| Floresta 2 | E | 10 | 22-fev-11 | 3 | *Didelphis* | *marsupialis* | DIMA | 117 | M | 0 |
| Floresta 1 | C | 50 | 22-fev-11 | 3 | *Makalata* | *didelphoides* | MADI | 116 | M | 0 |
| Floresta 1 | C | 54 | 23-fev-11 | 3 | *Oecomys* | sp. | OECO | 994 | F | 0 |
| Floresta 1 | C | 36 | 23-fev-11 | 3 | *Monodelphis* | *sp. D* | MOsp. | 118 | F | 1 |
| Floresta 1 | G | 42 | 23-fev-11 | 3 | *Oecomys* | sp. | OECO | 119 | M | 0 |
| Floresta 1 | E | 47 | 23-fev-11 | 3 | *Neacomys* | *cf. paracou* | NEPA | 120 | M | 0 |
| Floresta 1 | E | 37 | 23-fev-11 | 3 | *Neacomys* | *cf. paracou* | NEPA | 121 | M |  |
| Floresta 1 | A | 25 | 23-fev-11 | 3 | *Oecomys* | sp. | OECO | 122 | F | 0 |
| Floresta 2 | G | 28 | 23-fev-11 | 3 | *Oecomys* | sp. | OECO | 123 | F | 0 |
| Floresta 2 | C | 5 | 23-fev-11 | 3 | *Oecomys* | sp. | OECO | 4806/4878 | M | 0 |
| Floresta 2 | B | 51 | 23-fev-11 | 3 | *Euryoryzomys* | *emmonsae* | EUEM |  | F | 0 |
| Floresta 2 | E | 4 | 23-fev-11 | 3 | *Euryoryzomys* | *emmonsae* | EUEM | 124 | F | 0 |
| Floresta 2 | G | 34 | 23-fev-11 | 3 | *Euryoryzomys* | *emmonsae* | EUEM | 125 | F | 0 |
| Floresta 2 | E | 10 | 23-fev-11 | 3 | *Neacomys* | *cf. paracou* | NEPA | 126 | M | 0 |
| Floresta 2 | A | 1 | 23-fev-11 | 3 | *Marmosops* | *pinheiroi* | MAPI | 127 | F |  |
| Floresta 2 | A | 1 | 23-fev-11 | 3 | *Oecomys* | sp. | OECO | 128 | F | 0 |
| Floresta 2 | A | 2 | 23-fev-11 | 3 | *Euryoryzomys* | *emmonsae* | EUEM | 71 | M | 0 |
| Floresta 2 | G | 15 | 23-fev-11 | 3 | *Euryoryzomys* | *emmonsae* | EUEM |  |  |  |
| Floresta 2 | D | 4 | 23-fev-11 | 3 | *Marmosops* | *pinheiroi* | MAPI | 130 | M | 0 |
| Floresta 1 | A | 1 | 23-fev-11 | 3 | *Monodelphis* | *glirina* | MOGL | 129 | F | 0 |
| Floresta 1 | D | 11 | 24-fev-11 | 3 | *Oecomys* | sp. | OECO | 131 | M | 0 |
| Floresta 1 | C | 12 | 24-fev-11 | 3 | *Oecomys* | sp. | OECO | 132 | F |  |
| Floresta 1 | G | 14 | 24-fev-11 | 3 | *Oecomys* | sp. | OECO | 133 | F | 0 |
| Floresta 1 | E | 15 | 24-fev-11 | 3 | *Oecomys* | sp. | OECO |  | F | 0 |
| Floresta 1 | A | 6 | 24-fev-11 | 3 | *Oecomys* | sp. | OECO | 134 |  |  |
| Floresta 1 | B | 4 | 24-fev-11 | 3 | *Neacomys* | *cf. paracou* | NEPA | 135 | M | 0 |
| Floresta 1 | A | 31 | 24-fev-11 | 3 | *Neacomys* | *cf. paracou* | NEPA | 136 | F | 0 |
| Floresta 1 | G | 5 | 24-fev-11 | 3 | *Oecomys* | sp. | OECO | 137 | F | 0 |
| Floresta 1 | G | 16 | 24-fev-11 | 3 | *Neacomys* | *cf. paracou* | NEPA | 96 | M | 0 |
| Floresta 2 | B | 2 | 24-fev-11 | 3 | *Oecomys* | sp. | OECO |  |  | 1 |
| Floresta 2 | G | 13 | 24-fev-11 | 3 | *Oecomys* | sp. | OECO |  |  | 0 |
| Floresta 2 | E | 1 | 24-fev-11 | 3 | *Oecomys* | sp. | OECO |  | M | 0 |
| Floresta 2 | C | 12 | 24-fev-11 | 3 | *Oecomys* | sp. | OECO |  |  | 0 |
| Floresta 2 | C | 15 | 24-fev-11 | 3 | *Euryoryzomys* | *emmonsae* | EUEM | 126 |  | 0 |
| Floresta 2 | D | 11 | 24-fev-11 | 3 | *Euryoryzomys* | *emmonsae* | EUEM | 128 |  | 1 |
| Floresta 1 | C | 6 | 24-fev-11 | 3 | *Neacomys* | *cf. paracou* | NEPA |  |  | 1 |
| Floresta 1 | D | 11 | 24-fev-11 | 3 | *Oecomys* | sp. | OECO |  |  | 0 |
| Floresta 1 | C | 5 | 24-fev-11 | 3 | *Oecomys* | sp. | OECO |  |  | 0 |
| Floresta 1 | B | 6 | 24-fev-11 | 3 | *Monodelphis* | *glirina* | MOGL |  |  |  |
| Floresta 1 | A | 4 | 24-fev-11 | 3 | *Oxymycterus* | *amazonicus* | OXAM |  |  |  |
| Floresta 2 | B | 8 | 24-fev-11 | 3 | *Euryoryzomys* | *emmonsae* | EUEM |  |  | 0 |
| Floresta 2 | D | 14 | 24-fev-11 | 3 | *Oecomys* | sp. | OECO | 116 |  | 0 |
| Floresta 2 | C | 8 | 24-fev-11 | 3 | *Oecomys* | sp. | OECO | 106 |  | 1 |
| Floresta 2 | D | 14 | 24-fev-11 | 3 | *Monodelphis* | *sp. D* | MOsp. | 129 |  | 1 |
| Floresta 2 | G | 11 | 24-fev-11 | 3 | *Euryoryzomys* | *emmonsae* | EUEM | 138 |  | 1 |
| Floresta 2 | D | 14 | 24-fev-11 | 3 | *Oecomys* | sp. | OECO | 139 | M | 1 |
| Floresta 2 | E | 11 | 24-fev-11 | 3 | *Euryoryzomys* | *emmonsae* | EUEM | 140 | M | 0 |
| Floresta 2 | G | 1 | 24-fev-11 | 3 | *Monodelphis* | *sp. D* | MOsp. | 141 | F | 0 |
| Floresta 2 | B | 8 | 24-fev-11 | 3 | *Marmosops* | *pinheiroi* | MAPI | 51 | M | 0 |
| Floresta 2 | D | 6 | 24-fev-11 | 3 | *Neacomys* | *cf. paracou* | NEPA | 135 |  | 1 |
| Floresta 2 | G | 11 | 24-fev-11 | 3 | *Euryoryzomys* | *emmonsae* | EUEM | 142 |  | 1 |
| Floresta 2 | G | 11 | 24-fev-11 | 3 | *Euryoryzomys* | *emmonsae* | EUEM | 143 | F | 0 |
| Floresta 2 | C | 2 | 24-fev-11 | 3 | *Metachirus* | *nudicaudatus* | MENU | 144 | M | 0 |
| Floresta 2 | A | 12 | 24-fev-11 | 3 | *Neacomys* | *cf. paracou* | NEPA | 145 | M | 0 |
| Floresta 2 | A | 11 | 24-fev-11 | 3 | *Marmosops* | *pinheiroi* | MAPI | 146 | M | 0 |
| Floresta 2 | G | 7 | 24-fev-11 | 3 |  | *sp.* | NEPA | 147 | F | 0 |
| Floresta 2 | C | 11 | 24-fev-11 | 3 | *Oecomys* | sp. | OECO | 19 | M | 0 |
| Floresta 2 | A | 3 | 24-fev-11 | 3 | *Euryoryzomys* | *emmonsae* | EUEM | 148 | F | 1 |
| Floresta 2 | C | 9 | 24-fev-11 | 3 | *Monodelphis* | *glirina* | MOGL | 149 | M | 0 |
| Floresta 2 | A | 6 | 24-fev-11 | 3 | *Euryoryzomys* | *emmonsae* | EUEM | 151 | M | 0 |
| Floresta 1 | A | 1 | 25-fev-11 | 3 | *Monodelphis* | *glirina* | MOGL | 152 | M | 0 |
| Floresta 1 | B | 4 | 25-fev-11 | 3 | *Monodelphis* | *sp. D* | MOsp. |  | M | 0 |
| Floresta 1 | C | 10 | 25-fev-11 | 3 | *Akodon* | *aff. cursor* | AKCU | 153 | M | 0 |
| Floresta 2 | D | 6 | 25-fev-11 | 3 | *Oecomys* | sp. | OECO | 145 | M | 0 |
| Floresta 1 | G | 7 | 25-fev-11 | 3 | *Euryoryzomys* | *emmonsae* | EUEM |  | F | 0 |
| Floresta 1 | G | 4 | 25-fev-11 | 3 | *Oecomys* | sp. | OECO | 155 |  | 0 |
| Floresta 1 | G | 13 | 25-fev-11 | 3 | *Oecomys* | sp. | OECO | 156 | M | 0 |
| Floresta 1 | G | 7 | 25-fev-11 | 3 | *Oxymycterus* | *amazonicus* | OXAM | 157 | M | 0 |
| Floresta 1 | G | 7 | 25-fev-11 | 3 | *Oecomys* | sp. | OECO | 158 | M | 0 |
| Floresta 1 | D | 9 | 25-fev-11 | 3 | *Neacomys* | *cf. paracou* | NEPA | 96 | M | 0 |
| Floresta 1 | G | 7 | 25-fev-11 | 3 | *Oecomys* | sp. | OECO | 119 |  | 1 |
| Floresta 1 | B | 8 | 25-fev-11 | 3 | *Oecomys* | sp. | OECO | 53 |  | 1 |
| Floresta 1 | E | 15 | 25-fev-11 | 3 | *Monodelphis* | *sp. D* | MOsp. |  |  | 1 |
| Floresta 1 | C | 12 | 25-fev-11 | 3 | *Neacomys* | *cf. paracou* | NEPA | 130 |  |  |
| Floresta 1 | B | 8 | 25-fev-11 | 3 | *Oecomys* | sp. | OECO |  |  | 1 |
| Floresta 1 | A | 6 | 25-fev-11 | 3 | *Oecomys* | sp. | OECO | 159 |  | 0 |
| Floresta 1 | P | 1 | 25-fev-11 | 3 | *Oecomys* | sp. | OECO | 160 | M | 0 |
| Floresta 1 | E | 3 | 25-fev-11 | 3 | *Oecomys* | sp. | OECO | 161 | F | 0 |
| Floresta 1 | G | 6 | 25-fev-11 | 3 | *Oecomys* | sp. | OECO | 162 | M | 0 |
| Floresta 1 | A | 19 | 25-fev-11 | 3 | *Oecomys* | sp. | OECO |  | M | 0 |
| Floresta 1 | A | 31 | 25-fev-11 | 3 | *Oecomys* | sp. | OECO | 163 |  | 0 |
| Floresta 1 | E | 12 | 25-fev-11 | 3 | *Oecomys* | sp. | OECO | 164 | F | 0 |
| Floresta 2 | A | 32 | 25-fev-11 | 3 | *Hylaeamys* | *megacephalus* | HYME | 43 | F | 0 |
| Floresta 2 | D | 13 | 25-fev-11 | 3 | *Oecomys* | sp. | OECO |  | M | 1 |
| Floresta 2 | A | 22 | 25-fev-11 | 3 | *Oecomys* | sp. | OECO | 165 | F | 0 |
| Floresta 2 | E | 11 | 25-fev-11 | 3 | *Monodelphis* | *sp. D* | MOsp. | 166 | F | 0 |
| Floresta 2 | A | 30 | 25-fev-11 | 3 | *Euryoryzomys* | *emmonsae* | EUEM | 167 | F | 0 |
| Floresta 2 | B | 26 | 25-fev-11 | 3 | *Oecomys* | sp. | OECO | 168 | M | 0 |
| Floresta 2 | D | 1 | 25-fev-11 | 3 | *Oecomys* | sp. | OECO | 169 | M | 0 |
| Floresta 2 | A | 21 | 25-fev-11 | 3 | *Oecomys* | sp. | OECO | 170 | M | 0 |
| Floresta 2 | D | 59 | 25-fev-11 | 3 | *Oecomys* | sp. | OECO | 171 | F | 0 |
| Floresta 2 | D | 14 | 25-fev-11 | 3 | *Oecomys* | sp. | OECO | 172 | M | 0 |
| Floresta 2 | A | 2 | 25-fev-11 | 3 | *Oecomys* | sp. | OECO |  | M | 0 |
| Floresta 2 | G | 7 | 25-fev-11 | 3 | *Oecomys* | sp. | OECO | 173 |  |  |
| Floresta 2 | D | 9 | 25-fev-11 | 3 | *Euryoryzomys* | *emmonsae* | EUEM | 155 | M | 0 |
| Floresta 2 | D | 4 | 25-fev-11 | 3 | *Oecomys* | sp. | OECO | 174 | M | 1 |
| Floresta 2 | D | 15 | 25-fev-11 | 3 | *Marmosops* | *pinheiroi* | MAPI | 175 | F | 0 |
| Floresta 2 | G | 11 | 25-fev-11 | 3 | *Oecomys* | sp. | OECO | 176 | M | 0 |
| Floresta 2 | B | 1 | 25-fev-11 | 3 | *Marmosa* | *demerarae* | MADE | 177 | M | 0 |
| Floresta 2 | C | 1 | 25-fev-11 | 3 | *Proechimys* | *roberti* | PRRO | 178 | F | 0 |
| Floresta 2 | C | 3 | 25-fev-11 | 3 | *Euryoryzomys* | *emmonsae* | EUEM | 179 | M | 0 |
| Floresta 2 | G | 6 | 25-fev-11 | 3 | *Monodelphis* | *sp. D* | MOsp. | 173 | F | 0 |
| Floresta 1 | D | 12 | 26-fev-11 | 3 | *Oecomys* | sp. | OECO | 180 | M | 0 |
| Floresta 1 | G | 7 | 26-fev-11 | 3 | *Oecomys* | sp. | OECO | 181 | F | 0 |
| Floresta 2 | G | 3 | 26-fev-11 | 3 | *Oecomys* | sp. | OECO | 23 | F | 0 |
| Floresta 2 | D | 4 | 26-fev-11 | 3 | *Oecomys* | sp. | OECO | 182 | M | 1 |
| Floresta 2 | G | 4 | 26-fev-11 | 3 | *Euryoryzomys* | *emmonsae* | EUEM | 183 | F | 0 |
| Floresta 1 | E | 13 | 27-fev-11 | 3 | *Oecomys* | sp. | OECO | 184 | M | 0 |
| Floresta 1 | E | 2 | 27-fev-11 | 3 | *Monodelphis* | *sp. D* | MOsp. | 185 | M | 0 |
| Floresta 1 | D | 11 | 27-fev-11 | 3 | *Monodelphis* | *glirina* | MOGL | 186 | F | 0 |
| Floresta 1 | D | 9 | 27-fev-11 | 3 | *Monodelphis* | *sp. D* | MOsp. | 187 | F | 0 |
| Floresta 1 | D | 7 | 27-fev-11 | 3 | *Akodon* | *aff. cursor* | AKCU | 12 | M | 0 |
| Floresta 2 | G | 6 | 27-fev-11 | 3 | *Euryoryzomys* | *emmonsae* | EUEM | 139 | F | 1 |
| Floresta 2 | G | 6 | 27-fev-11 | 3 | *Euryoryzomys* | *emmonsae* | EUEM | 188 |  | 1 |
| Floresta 2 | D | 3 | 27-fev-11 | 3 | *Euryoryzomys* | *emmonsae* | EUEM | 189 | F | 0 |
| Floresta 2 | G | 8 | 27-fev-11 | 3 | *Marmosops* | *pinheiroi* | MAPI | 190 | M | 0 |
| Floresta 2 | C | 14 | 27-fev-11 | 3 | *Oecomys* | sp. | OECO | 152 | F | 0 |
| Floresta 2 | C | 14 | 27-fev-11 | 3 | *Oxymycterus* | *amazonicus* | OXAM | 194 |  | 1 |
| Floresta 1 | B | 5 | 27-fev-11 | 3 | *Euryoryzomys* | *emmonsae* | EUEM | 28 | F | 0 |
| Floresta 1 | B | 2 | 27-fev-11 | 3 | *Oecomys* | sp. | OECO | 195 |  | 1 |
| Floresta 1 | E | 13 | 28-fev-11 | 3 | *Oecomys* | sp. | OECO | 196 | M | 0 |
| Floresta 1 | E | 12 | 28-fev-11 | 3 | *Neacomys* | *cf. paracou* | NEPA | 197 | F | 0 |
| Floresta 1 | G | 14 | 28-fev-11 | 3 | *Oecomys* | sp. | OECO | 198 | F | 0 |
| Floresta 1 | B | 2 | 28-fev-11 | 3 | *Monodelphis* | *glirina* | MOGL | 199 | F | 0 |
| Floresta 1 | B | 3 | 28-fev-11 | 3 | *Rhipidomys* | *emiliae* | RHEM | 201 | F | 0 |
| Floresta 1 | B | 9 | 28-fev-11 | 3 | *Oecomys* | sp. | OECO | 202 | F | 0 |
| Floresta 1 | E | 7 | 28-fev-11 | 3 | *Oecomys* | sp. | OECO | 203 | M | 0 |
| Floresta 2 | C | 5 | 28-fev-11 | 3 |  | *sp.* | OECO | 204 | F | 0 |
| Floresta 1 | E | 4 | 28-fev-11 | 3 | *Oecomys* | sp. | OECO |  | F | 0 |
| Floresta 1 | C | 15 | 28-fev-11 | 3 | *Oecomys* | *sp.* | OECO | 30 |  |  |
| Floresta 1 | D | 11 | 28-fev-11 | 3 | *Oecomys* | sp. | OECO | 205 |  | 1 |
| Floresta 1 | E | 7 | 28-fev-11 | 3 | *Oecomys* | sp. | OECO |  | F | 0 |
| Floresta 1 | E | 7 | 1-mar-11 | 3 | *Neacomys* | *cf. paracou* | NEPA | 207 |  |  |
| Floresta 1 | G | 3 | 1-mar-11 | 3 | *Neacomys* | *cf. paracou* | NEPA | 96 | F | 0 |
| Floresta 1 | G | 7 | 1-mar-11 | 3 | *Monodelphis* | *sp. D* | MOsp. | 47 | M | 1 |
| Floresta 1 | A | 4 | 1-mar-11 | 3 | *Oecomys* | sp. | OECO | 206 | F | 1 |
| Floresta 1 | E | 12 | 1-mar-11 | 3 |  | *sp.* | OECO | 208 | F | 0 |
| Floresta 1 | D | 11 | 1-mar-11 | 3 | *Oecomys* | sp. | OECO |  | F | 0 |
| Floresta 1 | D | 11 | 1-mar-11 | 3 | *Oecomys* | sp. | OECO | 210 |  | 0 |
| Floresta 1 | E | 4 | 1-mar-11 | 3 | *Monodelphis* | *sp. D* | MOsp. |  | M | 0 |
| Floresta 1 | B | 12 | 1-mar-11 | 3 | *Neacomys* | *cf. paracou* | NEPA | 211 |  | 0 |
| Floresta 1 | D | 7 | 1-mar-11 | 3 | *Oecomys* | sp. | OECO |  | F | 0 |
| Floresta 1 | G | 15 | 1-mar-11 | 3 | *Oecomys* | sp. | OECO |  |  |  |
| Floresta 1 | G | 5 | 1-mar-11 | 3 | *Oecomys* | sp. | OECO | 212 | M |  |
| Floresta 1 | C | 4 | 1-mar-11 | 3 | *Oecomys* | sp. | OECO | 213 | M | 0 |
| FLORESTA 1 | G | 6 | 3-jul-11 | 4 | *Neacomys* | *cf. paracou* | NEPA | 214 | M | 0 |
| FLORESTA 2 | G | 14 | 3-jul-11 | 4 | *Marmosops* | *pinheiroi* | MAPI | 219 | F | 0 |
| FLORESTA 2 | D | 12 | 3-jul-11 | 4 | *Marmosa* | *murina* | MAMU | 61 | M | 0 |
| FLORESTA 2 | E | 3 | 3-jul-11 | 4 | *Monodelphis* | *glirina* | MOGL | 215 |  | 1 |
| FLORESTA 1 | G | 13 | 5-jul-11 | 4 | *Monodelphis* | *sp. D* | MOsp. | 994 | M | 0 |
| FLORESTA 2 | C | 7 | 6-jul-11 | 4 | *Marmosops* | *pinheiroi* | MAPI | 216 | F | 1 |
| FLORESTA 1 | C | 8 | 8-jul-11 | 4 | *Monodelphis* | *sp. D* | MOsp. | 217 | M | 0 |
| FLORESTA 2 | C | 1 | 9-jul-11 | 4 | *Oecomys* | sp. | OECO | 169 | F | 0 |
| FLORESTA 1 | C | 5 | 9-jul-11 | 4 | *Neacomys* | *cf. paracou* | NEPA | 194 |  | 1 |
| FLORESTA 2 | D | 12 | 9-jul-11 | 4 | *Oecomys* | sp. | OECO | 918 |  | 1 |
| FLORESTA 1 | G | 9 | 9-jul-11 | 4 | *Oligoryzomys* | *microtis* | OLMI |  |  | 0 |
| FLORESTA 2 | D | 10 | 9-jul-11 | 4 | *Oecomys* | sp. | OECO |  |  | 0 |
| FLORESTA 2 | G | 7 | 9-jul-11 | 4 | *Oecomys* | sp. | OECO |  |  | 0 |
| FLORESTA 1 | C | 9 | 9-jul-11 | 4 | *Oecomys* | sp. | OECO |  |  | 0 |
| FLORESTA 1 | G | 14 | 9-jul-11 | 4 | *Neacomys* | *cf. paracou* | NEPA | 196 |  | 0 |
| FLORESTA 2 | E | 6 | 9-jul-11 | 4 | *Oecomys* | sp. | OECO |  |  | 1 |
| FLORESTA 2 | E | 3 | 9-jul-11 | 4 | *Oecomys* | sp. | OECO |  |  | 0 |
| FLORESTA 2 | A | 2 | 9-jul-11 | 4 | *Monodelphis* | *sp. D* | MOsp. |  | M | 0 |
| FLORESTA 1 | E | 12 | 9-jul-11 | 4 | *Monodelphis* | *sp. D* | MOsp. |  |  | 0 |
| FLORESTA 1 | D | 2 | 9-jul-11 | 4 | *Monodelphis* | *sp. D* | MOsp. |  |  | 0 |
| FLORESTA 2 | G | 2 | 9-jul-11 | 4 | *Marmosops* | *pinheiroi* | MAPI |  |  | 0 |
| FLORESTA 1 | D | 2 | 10-jul-11 | 4 | *Monodelphis* | *sp. D* | MOsp. |  |  | 0 |
| FLORESTA 1 | G | 12 | 10-jul-11 | 4 | *Neacomys* | *cf. paracou* | NEPA |  |  | 0 |
| FLORESTA 1 | G | 7 | 10-jul-11 | 4 | *Oecomys* | sp. | OECO |  |  | 0 |
| FLORESTA 2 | G | 2 | 11-jul-11 | 4 | *Marmosops* | *pinheiroi* | MAPI |  |  | 0 |
| Canga 2 | C | 3 | 13-jul-11 | 4 | *Necromys* | *lasiurus* | NELA |  |  | 0 |
| Canga 2 | G | 5 | 13-jul-11 | 4 | *Necromys* | *lasiurus* | NELA |  |  | 0 |
| Canga 2 | D | 15 | 13-jul-11 | 4 | *Monodelphis* | *glirina* | MOGL | 220 |  | 0 |
| Canga 2 | E | 4 | 13-jul-11 | 4 | *Marmosa* | *murina* | MAMU |  | M | 0 |
| Canga 2 | E | 4 | 13-jul-11 | 4 | *Oxymycterus* | *amazonicus* | OXAM |  | M | 0 |
| Canga 2 | D | 7 | 13-jul-11 | 4 | *Marmosa* | *demerarae* | MADE |  |  | 0 |
| Canga 2 | G | 4 | 13-jul-11 | 4 | *Monodelphis* | *glirina* | MOGL | 221 | F | 0 |
| Canga 2 | G | 3 | 13-jul-11 | 4 | *Monodelphis* | *glirina* | MOGL |  | M | 0 |
| Canga 2 | A | 2 | 13-jul-11 | 4 | *Necromys* | *lasiurus* | NELA |  |  |  |
| Canga 2 | E | 3 | 13-jul-11 | 4 | *Necromys* | *lasiurus* | NELA |  |  |  |
| Canga 2 | A | 12 | 13-jul-11 | 4 | *Monodelphis* | *glirina* | MOGL |  | M |  |
| Canga 2 | E | 11 | 13-jul-11 | 4 | *Marmosa* | *demerarae* | MADE |  |  |  |
| Canga 2 | E | 4 | 13-jul-11 | 4 | *Necromys* | *lasiurus* | NELA | 222 |  |  |
| Canga 2 | E | 4 | 13-jul-11 | 4 | *Monodelphis* | *sp. D* | MOsp. | 997 | M |  |
| Canga 2 | C | 12 | 13-jul-11 | 4 | *Proechimys* | *roberti* | PRRO |  | M | 1 |
| Canga 2 | E | 11 | 13-jul-11 | 4 | *Monodelphis* | *glirina* | MOGL |  |  |  |
| Canga 2 | G | 15 | 13-jul-11 | 4 | *Monodelphis* | *glirina* | MOGL | 224 | F |  |
| Canga 2 | G | 1 | 13-jul-11 | 4 | *Necromys* | *lasiurus* | NELA | 226 | M |  |
| Canga 2 | G | 1 | 14-jul-11 | 4 | *Monodelphis* | *glirina* | MOGL |  | M |  |
| Canga 2 | E | 5 | 14-jul-11 | 4 | *Marmosa* | *demerarae* | MADE |  |  |  |
| Canga 2 | C | 6 | 14-jul-11 | 4 | *Monodelphis* | *glirina* | MOGL | 228 |  |  |
| Canga 2 | G | 4 | 14-jul-11 | 4 | *Necromys* | *lasiurus* | NELA | 667 | F |  |
| CANGA 2 | C | 14 | 14-jul-11 | 4 | *Oxymycterus* | *amazonicus* | OXAM |  |  |  |
| CANGA 2 | G | 3 | 14-jul-11 | 4 | *Monodelphis* | *glirina* | MOGL | 230 |  |  |
| CANGA 2 | E | 3 | 14-jul-11 | 4 | *Monodelphis* | *glirina* | MOGL |  |  |  |
| CANGA 2 | E | 5 | 14-jul-11 | 4 | *Monodelphis* | *glirina* | MOGL | 232 |  |  |
| CANGA 2 | C | 7 | 14-jul-11 | 4 | *Monodelphis* | *glirina* | MOGL | 234 |  |  |
| CANGA 2 | G | 2 | 14-jul-11 | 4 | *Marmosa* | *demerarae* | MADE |  | F |  |
| CANGA 2 | A | 9 | 14-jul-11 | 4 | *Monodelphis* | *glirina* | MOGL |  |  |  |
| CANGA 2 | D | 13 | 14-jul-11 | 4 | *Monodelphis* | *glirina* | MOGL |  |  |  |
| CANGA 2 | D | 9 | 14-jul-11 | 4 | *Monodelphis* | *glirina* | MOGL |  |  |  |
| CANGA 2 | E | 3 | 14-jul-11 | 4 | *Monodelphis* | *glirina* | MOGL |  |  |  |
| CANGA 2 | C | 9 | 14-jul-11 | 4 | *Monodelphis* | *glirina* | MOGL |  |  |  |
| CANGA 2 | E | 13 | 14-jul-11 | 4 | *Necromys* | *lasiurus* | NELA |  |  |  |
| CANGA 2 | A | 12 | 14-jul-11 | 4 | *Necromys* | *lasiurus* | NELA |  |  |  |
| Canga 2 | E | 15 | 15-jul-11 | 4 | *Monodelphis* | *glirina* | MOGL |  |  |  |
| CANGA 2 | G | 3 | 15-jul-11 | 4 | *Necromys* | *lasiurus* | NELA | 236 |  |  |
| CANGA 2 | D | 15 | 15-jul-11 | 4 | *Monodelphis* | *glirina* | MOGL | 238 | M | 0 |
| Canga 2 | A | 7 | 15-jul-11 | 4 | *Monodelphis* | *glirina* | MOGL | 240 |  | 0 |
| Canga 2 | G | 1 | 15-jul-11 | 4 | *Necromys* | *lasiurus* | NELA | 242 | M | 0 |
| Canga 2 | E | 2 | 15-jul-11 | 4 | *Monodelphis* | *glirina* | MOGL | 244 | M | 0 |
| Canga 2 | E | 5 | 15-jul-11 | 4 | *Monodelphis* | *glirina* | MOGL | 246 | F | 0 |
| Canga 2 | D | 15 | 15-jul-11 | 4 | *Monodelphis* | *glirina* | MOGL | 248 | M | 0 |
| Canga 2 | G | 6 | 15-jul-11 | 4 | *Marmosa* | *demerarae* | MADE | 250 | M | 0 |
| Canga 2 | G | 6 | 15-jul-11 | 4 | *Marmosa* | *demerarae* | MADE | 223 | M | 0 |
| Canga 2 | G | 4 | 15-jul-11 | 4 | *Oxymycterus* | *amazonicus* | OXAM | 225 | M | 0 |
| Canga 2 | G | 1 | 16-jul-11 | 4 | *Oxymycterus* | *amazonicus* | OXAM | 656 | F | 0 |
| Canga 2 | B | 3 | 16-jul-11 | 4 | *Oxymycterus* | *amazonicus* | OXAM | 234 |  | 1 |
| Canga 2 | A | 8 | 16-jul-11 | 4 | *Necromys* | *lasiurus* | NELA | 227 |  | 1 |
| Canga 2 | A | 5 | 16-jul-11 | 4 | *Proechimys* | *roberti* | PRRO | 229 | F | 0 |
| CANGA 2 | A | 4 | 16-jul-11 | 4 | *Oxymycterus* | *amazonicus* | OXAM | 222 | F | 0 |
| CANGA 2 | E | 10 | 16-jul-11 | 4 | *Oxymycterus* | *amazonicus* | OXAM | 231 | M | 1 |
| CANGA 2 | B | 2 | 16-jul-11 | 4 | *Marmosa* | *demerarae* | MADE | 233 | F | 0 |
| CANGA 2 | A | 2 | 16-jul-11 | 4 | *Marmosa* | *demerarae* | MADE |  | M | 0 |
| CANGA 2 | C | 12 | 16-jul-11 | 4 |  |  |  |  |  | 0 |
| CANGA 2 | D | 14 | 16-jul-11 | 4 | *Marmosa* | *murina* | MAMU |  |  | 0 |
| CANGA 2 | G | 15 | 16-jul-11 | 4 | *Monodelphis* | *glirina* | MOGL |  |  | 0 |
| Canga 2 | C | 6 | 16-jul-11 | 4 | *Necromys* | *lasiurus* | NELA |  |  | 0 |
| Canga 2 | E | 13 | 16-jul-11 | 4 | *Monodelphis* | *glirina* | MOGL |  |  | 0 |
| Canga 2 | D | 9 | 16-jul-11 | 4 | *Monodelphis* | *glirina* | MOGL |  |  | 0 |
| Canga 2 | E | 6 | 16-jul-11 | 4 | *Monodelphis* | *glirina* | MOGL |  |  | 0 |
| Canga 2 | E | 12 | 16-jul-11 | 4 | *Necromys* | *lasiurus* | NELA |  |  | 0 |
| Canga 2 | B | 15 | 16-jul-11 | 4 | *Monodelphis* | *glirina* | MOGL |  |  | 0 |
| Canga 2 | D | 14 | 16-jul-11 | 4 | *Monodelphis* | *glirina* | MOGL |  |  | 0 |
| Canga 2 | B | 2 | 16-jul-11 | 4 | *Monodelphis* | *glirina* | MOGL |  |  | 0 |
| Canga 2 | G | 2 | 16-jul-11 | 4 | *Necromys* | *lasiurus* | NELA |  |  | 0 |
| Canga 2 | G | 13 | 16-jul-11 | 4 | *Necromys* | *lasiurus* | NELA |  |  | 0 |
| Canga 2 | B | 7 | 16-jul-11 | 4 | *Necromys* | *lasiurus* | NELA | 235 |  | 1 |
| Canga 2 | C | 7 | 16-jul-11 | 4 | *Necromys* | *lasiurus* | NELA |  | M | 0 |
| Canga 2 | B | 5 | 17-jul-11 | 4 | *Oxymycterus* | *amazonicus* | OXAM |  |  |  |
| Canga 2 | D | 28 | 17-jul-11 | 4 | *Oxymycterus* | *amazonicus* | OXAM | 237 | F |  |
| Canga 2 | E | 22 | 17-jul-11 | 4 | *Oxymycterus* | *amazonicus* | OXAM | 239 | F |  |
| Canga 2 | E | 10 | 17-jul-11 | 4 | *Monodelphis* | *glirina* | MOGL | 241 | M | 0 |
| Canga 2 | E | 20 | 17-jul-11 | 4 | *Monodelphis* | *glirina* | MOGL | 243 | F | 0 |
| Canga 2 | E | 7 | 17-jul-11 | 4 | *Monodelphis* | *glirina* | MOGL | 245 | M | 0 |
| Canga 2 | E | 6 | 17-jul-11 | 4 | *Monodelphis* | *glirina* | MOGL | 247 | M | 0 |
| Canga 2 | E | 10 | 17-jul-11 | 4 | *Monodelphis* | *glirina* | MOGL | 249 | F | 0 |
| Canga 2 | A | 26 | 17-jul-11 | 4 | *Monodelphis* | *glirina* | MOGL | 251 | F | 0 |
| Canga 2 | A | 32 | 17-jul-11 | 4 | *Monodelphis* | *glirina* | MOGL | 252 | M | 0 |
| Canga 2 | G | 3 | 17-jul-11 | 4 | *Monodelphis* | *glirina* | MOGL |  | F | 0 |
| Canga 2 | D | 8 | 17-jul-11 | 4 | *Monodelphis* | *glirina* | MOGL |  |  |  |
| Canga 2 | D | 4 | 17-jul-11 | 4 | *Necromys* | *lasiurus* | NELA |  |  |  |
| Canga 2 | B | 5 | 17-jul-11 | 4 | *Necromys* | *lasiurus* | NELA | 230 |  |  |
| Canga 2 | G | 4 | 17-jul-11 | 4 | *Necromys* | *lasiurus* | NELA | 236 |  | 1 |
| Canga 2 | G | 2 | 17-jul-11 | 4 | *Necromys* | *lasiurus* | NELA | 253 |  | 1 |
| Canga 2 | D | 13 | 17-jul-11 | 4 | *Necromys* | *lasiurus* | NELA | 254 | F | 0 |
| Canga 2 | E | 3 | 17-jul-11 | 4 | *Necromys* | *lasiurus* | NELA | 255 | M | 0 |
| Canga 2 | D | 3 | 17-jul-11 | 4 | *Monodelphis* | *glirina* | MOGL | 256 | F | 0 |
| Canga 2 | A | 6 | 17-jul-11 | 4 | *Monodelphis* | *glirina* | MOGL | 977 | F | 0 |
| CANGA 2 | E | 5 | 18-jul-11 | 4 | *Oxymycterus* | *amazonicus* | OXAM | 257 | M | 1 |
| CANGA 2 | A | 10 | 18-jul-11 | 4 | *Marmosa* | *demerarae* | MADE | 258 | M | 0 |
| CANGA 2 | E | 14 | 18-jul-11 | 4 | *Necromys* | *lasiurus* | NELA | 259 | M | 0 |
| CANGA 2 | B | 27 | 18-jul-11 | 4 | *Proechimys* | *roberti* | PRRO | 260 | M | 0 |
| CANGA 2 | D | 45 | 18-jul-11 | 4 | *Monodelphis* | *glirina* | MOGL | 261 | F | 0 |
| CANGA 2 | B | 43 | 18-jul-11 | 4 | *Oecomys* | sp. | OECO | 262 | M | 0 |
| CANGA 2 | E | 47 | 18-jul-11 | 4 | *Necromys* | *lasiurus* | NELA | 263 | M | 0 |
| CANGA 2 | B | 46 | 18-jul-11 | 4 | *Marmosa* | *demerarae* | MADE | 264 | M | 0 |
| CANGA 2 | C | 52 | 18-jul-11 | 4 | *Oxymycterus* | *amazonicus* | OXAM | 248 | M | 0 |
| CANGA 2 | E | 20 | 18-jul-11 | 4 | *Monodelphis* | *glirina* | MOGL | 224 |  | 1 |
| CANGA 2 | A | 8 | 18-jul-11 | 4 | *Monodelphis* | *glirina* | MOGL |  |  | 1 |
| CANGA 2 | B | 54 | 18-jul-11 | 4 | *Monodelphis* | *glirina* | MOGL | 238 |  | 0 |
| CANGA 2 | D | 10 | 18-jul-11 | 4 | *Monodelphis* | *glirina* | MOGL |  |  | 1 |
| CANGA 2 | E | 12 | 18-jul-11 | 4 | *Monodelphis* | *glirina* | MOGL |  |  |  |
| CANGA 2 | C | 7 | 18-jul-11 | 4 | *Necromys* | *lasiurus* | NELA |  |  |  |
| CANGA 2 | D | 4 | 18-jul-11 | 4 | *Monodelphis* | *glirina* | MOGL |  |  |  |
| CANGA 2 | C | 7 | 18-jul-11 | 4 | *Euryoryzomys* | *emmonsae* | EUEM |  |  |  |
| CANGA 2 | B | 8 | 18-jul-11 | 4 | *Monodelphis* | *glirina* | MOGL |  |  |  |
| CANGA 2 | G | 8 | 18-jul-11 | 4 | *Necromys* | *lasiurus* | NELA |  |  |  |
| CANGA 2 | E | 33 | 18-jul-11 | 4 | *Necromys* | *lasiurus* | NELA | 265 |  |  |
| CANGA 2 | G | 11 | 18-jul-11 | 4 | *Monodelphis* | *glirina* | MOGL | 266 | M | 0 |
| CANGA 2 | C | 7 | 18-jul-11 | 4 | *Necromys* | *lasiurus* | NELA | 267 | M | 0 |
| CANGA 2 | A | 4 | 18-jul-11 | 4 | *Monodelphis* | *glirina* | MOGL | 268 | M | 0 |
| CANGA 2 | A | 49 | 18-jul-11 | 4 | *Necromys* | *lasiurus* | NELA | 231 | M | 0 |
| CANGA 2 | D | 40 | 18-jul-11 | 4 | *Necromys* | *lasiurus* | NELA | 223 |  | 1 |
| CANGA 2 | G | 4 | 18-jul-11 | 4 | *Monodelphis* | *glirina* | MOGL | 269 |  | 1 |
|  | C | 26 | 18-jul-11 | 4 |  | *sp.* | EUEM |  |  | 0 |
| CANGA 2 | E | 4 | 18-jul-11 | 4 | *Monodelphis* | *glirina* | MOGL | 270 |  |  |
| CANGA 2 | E | 10 | 18-jul-11 | 4 | *Monodelphis* | *glirina* | MOGL | 271 | M | 0 |
| CANGA 2 | B | 40 | 18-jul-11 | 4 | *Monodelphis* | *glirina* | MOGL | 272 | F | 0 |
| CANGA 2 | B | 27 | 18-jul-11 | 4 | *Necromys* | *lasiurus* | NELA | 273 | M | 0 |
| FLORESTA 1 | D | 19 | 21-jul-11 | 4 | *Marmosa* | *demerarae* | MADE | 274 | M | 0 |
| FLORESTA 1 | G | 45 | 22-jul-11 | 4 | *Monodelphis* | *sp. D* | MOsp. | 244 | M | 0 |
| FLORESTA 1 | C | 39 | 24-jul-11 | 4 | *Monodelphis* | *glirina* | MOGL | 276 | M | 1 |
| FLORESTA 1 | C | 11 | 25-jul-11 | 4 | *Euryoryzomys* | *emmonsae* | EUEM | 277 | F | 0 |
| Canga 1 | E | 26 | 26-jul-11 | 4 | *Necromys* | *lasiurus* | NELA | 278 | F | 0 |
| Canga 1 | G | 44 | 27-jul-11 | 4 | *Monodelphis* | *glirina* | MOGL |  | F | 0 |
| Canga 1 | E | 49 | 27-jul-11 | 4 | *Monodelphis* | *glirina* | MOGL | 233 |  |  |
| Canga 1 | A | 39 | 27-jul-11 | 4 | *Monodelphis* | *glirina* | MOGL | 227 |  | 1 |
| Canga 1 | G | 43 | 27-jul-11 | 4 | *Monodelphis* | *glirina* | MOGL | 248 |  | 1 |
| Canga 1 | C | 11 | 27-jul-11 | 4 | *Monodelphis* | *glirina* | MOGL | 249 |  | 1 |
| Canga 1 | C | 16 | 27-jul-11 | 4 | *Monodelphis* | *glirina* | MOGL |  |  | 1 |
| Canga 1 | B | 26 | 27-jul-11 | 4 | *Monodelphis* | *glirina* | MOGL | 279 | M | 0 |
| Canga 1 | G | 11 | 27-jul-11 | 4 | *Monodelphis* | *glirina* | MOGL | 280 | M | 0 |
| Canga 1 | B | 4 | 27-jul-11 | 4 | *Monodelphis* | *glirina* | MOGL |  | F | 0 |
| Canga 1 | D | 7 | 27-jul-11 | 4 | *Monodelphis* | *glirina* | MOGL |  |  | 0 |
| Canga 1 | G | 6 | 27-jul-11 | 4 | *Necromys* | *lasiurus* | NELA | 281 | M |  |
| Canga 1 | D | 10 | 27-jul-11 | 4 | *Oxymycterus* | *amazonicus* | OXAM | 282 | M | 0 |
| Canga 1 | G | 44 | 27-jul-11 | 4 | *Oxymycterus* | *amazonicus* | OXAM | 871 | F | 0 |
| Canga 1 | B | 23 | 28-jul-11 | 4 | *Necromys* | *lasiurus* | NELA | 283 | M | 1 |
| Canga 1 | E | 53 | 28-jul-11 | 4 | *Necromys* | *lasiurus* | NELA | 284 | F | 0 |
| Canga 1 | D | 2 | 28-jul-11 | 4 | *Oxymycterus* | *amazonicus* | OXAM | 285 | F | 0 |
| Canga 1 | E | 26 | 28-jul-11 | 4 | *Monodelphis* | *glirina* | MOGL | 881 | F | 0 |
| Canga 1 | E | 48 | 28-jul-11 | 4 | *Marmosa* | *demerarae* | MADE | 286 | F | 1 |
| Canga 1 | C | 47 | 28-jul-11 | 4 | *Monodelphis* | *glirina* | MOGL | 287 | M | 0 |
| Canga 1 | G | 55 | 28-jul-11 | 4 | *Monodelphis* | *glirina* | MOGL | 288 | F | 0 |
| Canga 1 | D | 29 | 28-jul-11 | 4 | *Necromys* | *lasiurus* | NELA | 289 | F | 0 |
| Canga 1 | E | 45 | 28-jul-11 | 4 | *Monodelphis* | *glirina* | MOGL | 291 | M | 0 |
| Canga 1 | D | 41 | 28-jul-11 | 4 | *Oxymycterus* | *amazonicus* | OXAM | 290 | F | 0 |
| Canga 1 | A | 36 | 28-jul-11 | 4 | *Necromys* | *lasiurus* | NELA | 859 | F | 0 |
| Canga 1 | G | 41 | 28-jul-11 | 4 | *Necromys* | *lasiurus* | NELA |  | F | 1 |
| Canga 1 | B | 40 | 28-jul-11 | 4 | *Monodelphis* | *glirina* | MOGL |  | M | 0 |
| Canga 1 | E | 46 | 28-jul-11 | 4 | *Necromys* | *lasiurus* | NELA |  | F | 0 |
| Canga 1 | C | 29 | 28-jul-11 | 4 | *Necromys* | *lasiurus* | NELA |  | F | 0 |
| Canga 1 | A | 6 | 28-jul-11 | 4 | *Necromys* | *lasiurus* | NELA | 292 | F | 0 |
| Canga 1 | B | 32 | 29-jul-11 | 4 | *Monodelphis* | *glirina* | MOGL | 293 | M | 0 |
| Canga 1 | B | 46 | 29-jul-11 | 4 | *Oxymycterus* | *amazonicus* | OXAM | 294 | F | 0 |
| Canga 1 | D | 22 | 29-jul-11 | 4 | *Monodelphis* | *glirina* | MOGL | 295 | M | 0 |
| Canga 1 | E | 42 | 29-jul-11 | 4 | *Monodelphis* | *glirina* | MOGL | 296 | F | 0 |
| Canga 1 | A | 17 | 29-jul-11 | 4 | *Monodelphis* | *glirina* | MOGL | 297 | F | 0 |
| Canga 1 | B | 1 | 29-jul-11 | 4 | *Oxymycterus* | *amazonicus* | OXAM | 298 | F | 0 |
| Canga 1 | A | 31 | 29-jul-11 | 4 | *Monodelphis* | *glirina* | MOGL | 299 | M | 0 |
| Canga 1 | D | 45 |  | 4 | *Monodelphis* | *glirina* | MOGL | 300 | F | 0 |
| Canga 1 | E | 45 | 29-jul-11 | 4 | *Monodelphis* | *glirina* | MOGL | 301 | F | 0 |
| Canga 1 | C | 20 | 29-jul-11 | 4 | *Monodelphis* | *glirina* | MOGL | 812 | M | 0 |
| Canga 1 | D | 12 | 29-jul-11 | 4 | *Monodelphis* | *glirina* | MOGL | 288 | M | 1 |
| Canga 1 | D | 42 | 29-jul-11 | 4 | *Monodelphis* | *glirina* | MOGL |  | F | 1 |
| Canga 1 | G | 48 | 29-jul-11 | 4 | *Oxymycterus* | *amazonicus* | OXAM |  | M | 0 |
| Canga 1 | E | 13 | 29-jul-11 | 4 | *Oxymycterus* | *amazonicus* | OXAM |  |  | 0 |
| Canga 1 | D | 31 | 29-jul-11 | 4 | *Necromys* | *lasiurus* | NELA | 893 | F | 0 |
| Canga 1 | D | 13 | 30-jul-11 | 4 | *Necromys* | *lasiurus* | NELA | 291 | F | 1 |
| Canga 1 | A | 3 | 30-jul-11 | 4 | *Necromys* | *lasiurus* | NELA | 287 | F | 1 |
| Canga 1 | C | 27 | 30-jul-11 | 4 | *Oxymycterus* | *amazonicus* | OXAM | 302 | F | 1 |
| Canga 1 | B | 32 | 30-jul-11 | 4 | *Rhipidomys* | *emiliae* | RHEM | 303 | F | 0 |
| Canga 1 | A | 3 | 30-jul-11 | 4 | *Necromys* | *lasiurus* | NELA | 304 | M | 0 |
| Canga 1 | B | 36 | 30-jul-11 | 4 | *Oxymycterus* | *amazonicus* | OXAM | 305 | F | 0 |
| Canga 1 | G | 59 | 30-jul-11 | 4 | *Monodelphis* | *glirina* | MOGL | 306 | F | 0 |
| Canga 1 | E | 13 | 30-jul-11 | 4 | *Monodelphis* | *glirina* | MOGL | 307 | F | 0 |
| Canga 1 | C | 4 | 30-jul-11 | 4 | *Monodelphis* | *glirina* | MOGL | 296 | F | 0 |
| Canga 1 | A | 4 | 30-jul-11 | 4 | *Monodelphis* | *glirina* | MOGL | 911 | F | 1 |
| Canga 1 | G | 41 | 30-jul-11 | 4 | *Monodelphis* | *glirina* | MOGL |  | F | 1 |
| Canga 1 | C | 37 | 30-jul-11 | 4 | *Necromys* | *lasiurus* | NELA |  |  |  |
| Canga 1 | D | 41 | 30-jul-11 | 4 | *Necromys* | *lasiurus* | NELA |  |  |  |
| Canga 1 | E | . | 30-jul-11 | 4 | *Necromys* | *lasiurus* | NELA |  |  |  |
| Canga 1 | D | 46 | 30-jul-11 | 4 | *Monodelphis* | *glirina* | MOGL |  | F |  |
| Canga 1 | A | 17 | 31-jul-11 | 4 | *Monodelphis* | *glirina* | MOGL | 308 |  |  |
| Canga 1 | E | 13 | 31-jul-11 | 4 | *Oxymycterus* | *amazonicus* | OXAM | 309 | F | 0 |
| Canga 1 | B | 9 | 31-jul-11 | 4 | *Necromys* | *lasiurus* | NELA | 310 | F | 0 |
| Canga 1 | D | 45 | 31-jul-11 | 4 | *Necromys* | *lasiurus* | NELA | 311 | M | 0 |
| Canga 1 | C | 23 | 31-jul-11 | 4 | *Oxymycterus* | *amazonicus* | OXAM | 312 | M | 0 |
| Canga 1 | A | 23 | 31-jul-11 | 4 | *Oxymycterus* | *amazonicus* | OXAM | 313 | F | 0 |
| Canga 1 | A | 10 | 31-jul-11 | 4 | *Necromys* | *lasiurus* | NELA | 294 | F | 0 |
| Canga 1 | E | 24 | 31-jul-11 | 4 | *Oxymycterus* | *amazonicus* | OXAM | 314 |  | 1 |
| Canga 1 | C | 21 | 31-jul-11 | 4 | *Monodelphis* | *glirina* | MOGL | 315 | M | 0 |
| Canga 1 | C | 33 | 31-jul-11 | 4 | *Monodelphis* | *glirina* | MOGL | 285 | M | 0 |
| Canga 1 | D | 17 | 31-jul-11 | 4 | *Monodelphis* | *glirina* | MOGL | 287 |  | 1 |
| Canga 1 | B | 35 | 31-jul-11 | 4 | *Monodelphis* | *glirina* | MOGL | 308 |  | 1 |
| Canga 1 | A | 13 | 31-jul-11 | 4 | *Monodelphis* | *glirina* | MOGL |  |  | 1 |
| Canga 1 | E | 21 | 1-ago-11 | 4 | *Necromys* | *lasiurus* | NELA |  |  |  |
| Canga 1 | D | 1 | 1-ago-11 | 4 | *Necromys* | *lasiurus* | NELA | 296 |  |  |
| Canga 1 | C | 29 | 1-ago-11 | 4 | *Necromys* | *lasiurus* | NELA |  |  | 1 |
| Canga 1 | C | 5 | 1-ago-11 | 4 | *Necromys* | *lasiurus* | NELA | 989 |  |  |
| Canga 1 | E | 20 | 1-ago-11 | 4 | *Necromys* | *lasiurus* | NELA |  |  | 1 |
| Canga 1 | C | 21 | 1-ago-11 | 4 | *Monodelphis* | *glirina* | MOGL | 316 |  |  |
| Canga 1 | D | 22 | 1-ago-11 | 4 | *Monodelphis* | *glirina* | MOGL | 317 | M | 0 |
| Canga 1 | B | 31 | 1-ago-11 | 4 | *Monodelphis* | *glirina* | MOGL | 318 | M | 0 |
| Canga 1 | A | 30 | 1-ago-11 | 4 | *Monodelphis* | *glirina* | MOGL | 319 | M | 0 |
| Canga 1 | C | 22 | 1-ago-11 | 4 | *Oxymycterus* | *amazonicus* | OXAM | 320 | M | 0 |
| FLORESTA 2 | A | 2 | 5-ago-11 | 4 | *Proechimys* | *roberti* | PRRO | 298 | M | 0 |
| FLORESTA 2 | A | 23 | 5-ago-11 | 4 | *Rhipidomys* | *emiliae* | RHEM |  |  | 1 |
| FLORESTA 2 | B | 15 | 5-ago-11 | 4 | *Euryoryzomys* | *emmonsae* | EUEM | 321/325 |  |  |
| FLORESTA 2 | E | 7 | 5-ago-11 | 4 | *Euryoryzomys* | *emmonsae* | EUEM | 322 | M | 0 |
| FLORESTA 2 | D | 25 | 5-ago-11 | 4 | *Marmosa* | *demerarae* | MADE | 891 | F | 0 |
| FLORESTA 2 | B | 10 | 5-ago-11 | 4 | *Euryoryzomys* | *emmonsae* | EUEM | 323 | M | 1 |
| FLORESTA 2 | E | 22 | 6-ago-11 | 4 | *Marmosa* | *demerarae* | MADE | 287 | M | 0 |
| FLORESTA 2 | A | 2 | 6-ago-11 | 4 | *Monodelphis* | *glirina* | MOGL | 293 | M | 1 |
| FLORESTA 2 | B | 45 | 7-ago-11 | 4 | *Oxymycterus* | *amazonicus* | OXAM | 302 | M | 1 |
| FLORESTA 2 | C | 5 | 7-ago-11 | 4 | *Marmosa* | *demerarae* | MADE |  | M | 1 |
| FLORESTA 2 | A | 39 | 7-ago-11 | 4 | *Monodelphis* | *sp. D* | MOsp. | 326 | M |  |
| FLORESTA 2 | B | 27 | 8-ago-11 | 4 | *Marmosa* | *demerarae* | MADE |  | M |  |
| FLORESTA 2 | B | 45 | 8-ago-11 | 4 | *Metachirus* | *nudicaudatus* | MENU |  | M |  |
| FLORESTA 2 | D | 25 | 8-ago-11 | 4 | *Philander* | *opossum* | PHOP |  | M |  |
